# Extracellular matrix protein-1 secretory isoform promotes ovarian cancer through increasing alternative mRNA splicing and stemness

**DOI:** 10.1038/s41467-021-24315-1

**Published:** 2021-07-09

**Authors:** Huijing Yin, Jingshu Wang, Hui Li, Yinjue Yu, Xiaoling Wang, Lili Lu, Cuiting Lv, Bin Chang, Wei Jin, Wenwen Guo, Chunxia Ren, Gong Yang

**Affiliations:** 1 Cancer Institute, Fudan University Shanghai Cancer Center, Shanghai, China; 2 Department of Oncology, Shanghai Medical School, Fudan University, Shanghai, China; 3 Central Laboratory, The Fifth People’s Hospital of Shanghai, Fudan University, Shanghai, China; 4 Center for Reproductive Medicine, Shuguang Hospital Affiliated to Shanghai University of Traditional Chinese Medicine, Shanghai, China; 5 Department of Pathology, Fudan University Shanghai Cancer Center, Shanghai, China; 6 Department of Pathology, The People’s Hospital of Guangxi Zhuang Autonomous Region, Guangxi, China; 7 Department of Pancreatic Surgery, Fudan University Shanghai Cancer Center, Shanghai, China

**Keywords:** Cancer stem cells, Cancer therapeutic resistance, Ovarian cancer, Cell signalling

## Abstract

Extracellular matrix protein-1 (ECM1) promotes tumorigenesis in multiple organs but the mechanisms associated to ECM1 isoform subtypes have yet to be clarified. We report in this study that the secretory ECM1a isoform induces tumorigenesis through the GPR motif binding to integrin αXβ2 and the activation of AKT/FAK/Rho/cytoskeleton signaling. The ATP binding cassette subfamily G member 1 (ABCG1) transduces the ECM1a-integrin αXβ2 interactive signaling to facilitate the phosphorylation of AKT/FAK/Rho/cytoskeletal molecules and to confer cancer cell cisplatin resistance through up-regulation of the CD326-mediated cell stemness. On the contrary, the non-secretory ECM1b isoform binds myosin and blocks its phosphorylation, impairing cytoskeleton-mediated signaling and tumorigenesis. Moreover, ECM1a induces the expression of the heterogeneous nuclear ribonucleoprotein L like (hnRNPLL) protein to favor the alternative mRNA splicing generating ECM1a. ECM1a, αXβ2, ABCG1 and hnRNPLL higher expression associates with poor survival, while ECM1b higher expression associates with good survival. These results highlight ECM1a, integrin αXβ2, hnRNPLL and ABCG1 as potential targets for treating cancers associated with ECM1-activated signaling.

## Abstract (web summary)

Extracellular matrix protein 1 (ECM1) has been associated with cancer but the underlying molecular mechanisms are not clear. Here, the authors show that while ECM1b isoform is a tumour suppressor, the secreted isoform ECM1a promotes tumourigenesis and chemoresistance through increasing stemness and alternative mRNA splicing in ovarian cancer.

## Introduction

The extracellular matrix (ECM) may contain molecules that are suitable markers or targets for cancer diagnosis and treatment. Recent findings suggest that the ECM participates in tumorigenesis, cancer metastasis, chemoresistance, and tumor microenvironment remodeling^1,2^. Among ECM components, ECM protein-1 (ECM1) is of particular interest. Lee et al. reported that ECM1 promotes trastuzumab resistance and the PKM2-mediated Warburg effect through activation of epidermal growth factor^3,4^. ECM1 controls cancer stem cell-like properties and epithelial-to-mesenchymal transition through stabilization of β-catenin expression^5^. A recent study has shown that ECM1 may regulate gastric cancer cell metastasis and glucose metabolism through ITB4/FAK/SOX2/HIF-1α signaling^6^. More interestingly, ECM1 may regulate the actin cytoskeletal architecture, leading to metastasis of aggressive breast cancer cells^7^. However, the detailed molecular mechanism associated with ECM1-mediated signaling in cancer cells is not quite clear. On the other hand, although many studies have shown that *ECM1* is an oncogene, one study has reported that *ECM1* is a tumor suppressor gene regulated through promoter hypermethylation in human hepatocellular carcinoma^8^, which is consistent with a recent report^9^. Therefore, whether ECM1 is tumorigenic still needs to be comprehensively studied.

The human *ECM1* gene encodes four subtypes that are generated from splicing variants: ECM1a, ECM1b, ECM1c, and ECM1d^10,11^. ECM1a, which is composed of 540 amino acids (aa), is expressed mainly in basal keratinocytes, dermal blood vessels, and the adnexal epithelium. ECM1b, which consists of 415 aa and lacks the aa encoded by the seventh exon of ECM1a and ECM1c, is expressed largely in the epithelial spiny and granular layers. ECM1c, whose cysteine-free domain contains 19 aa more than that of ECM1a, is expressed primarily in the epithelial basal layer. ECM1d contains only 57 aa from the N-termini of ECM1a, ECM1b, and ECM1c and has unclear functions^12,13^. While ECM1a has been identified to be oncogenic in multiple cancers, ECM1b was recently reported to localized to the endoplasmic reticulum and to function as a tumor suppressor in esophageal squamous cell carcinoma (ESCC) through regulation of MTORC2/MYC/MTORC1 signaling^9^. ECM1c may interact with the protein perlecan to regulate bone formation and angiogenesis^14^. Although the primary structure of ECM1, starting at the N-terminus, is known to consist of a signal peptide (19 aa), a cysteine-free domain, ECM1 repeat 1, ECM1 repeat 2, and a C-terminal domain^15^, the location of the ECM1 functional domain has not yet been reported. However, most mutations in ECM1 are located in exons 6 and 7 and thus may affect the functions of the three ECM1 isoforms in lipoid proteinosis^16^. Nevertheless, the detailed functions of ECM1 subtypes and the putative receptors that may interact with ECM1 and mediate ECM1-associated tumorigenic signaling have not yet been identified.

In the present study, we show that ECM1a activates AKT/FAK/Paxillin/Rac/cytoskeletal signaling and upregulates CD326 expression to control tumorigenesis and cisplatin resistance through Gly-Pro-Arg (GPR) motif-mediated interactions with integrin αXβ2, heterogeneous nuclear ribonucleoprotein L-like (hnRNPLL)-mediated alternative mRNA splicing, and ATP binding cassette subfamily G member 1 (ABCG1)-induced upregulation of cancer cell stemness.

## Results

### ECM1a, but not ECM1b, induces tumorigenesis

**Fig. 1:**
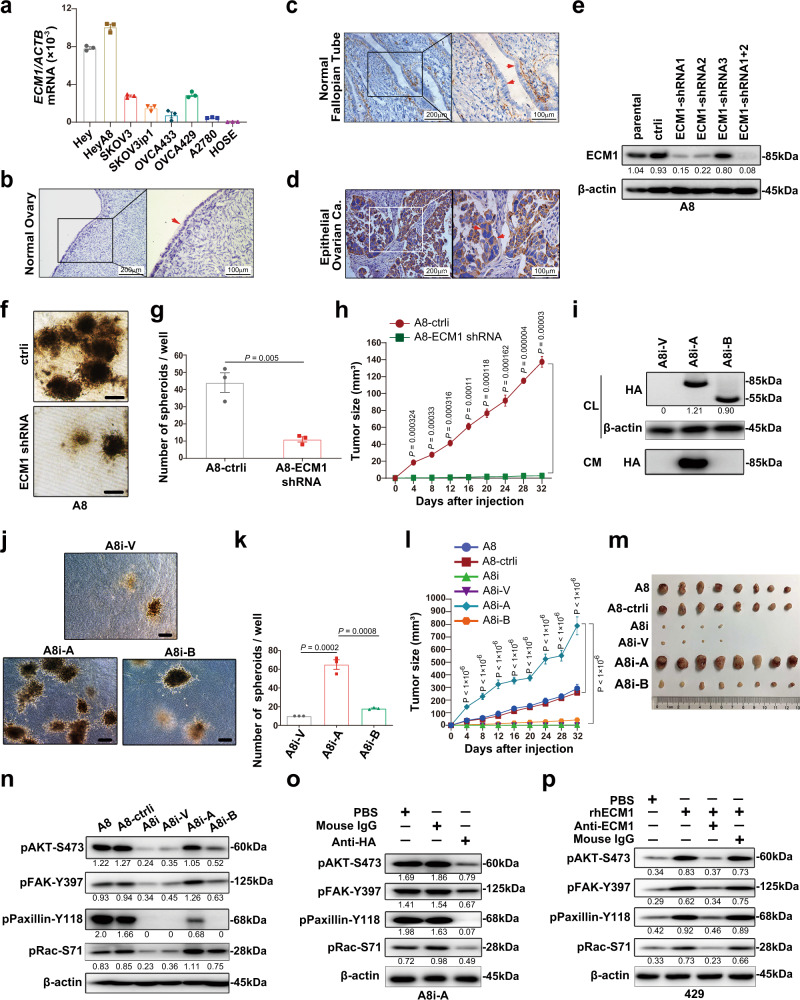
Inverse tumorigenicity of ECM1 subtypes. **a** Detection of ECM1 mRNA levels in CLs of ovarian cancer cell lines (Hey, HeyA8, SKOV3, SKOV3ip1, OVCA433, OVCA429, and A2780) and a normal cell line (HOSE) by qRT-PCR. *ACTB* (encoding β-actin) was used to normalize the expression of ECM1. Data are presented as mean ± SD. *n* = 3 biologically independent repeats. Representative images of ECM1 staining performed by IHC in normal ovary (**b**), normal fallopian tube (**c**), and ovarian carcinoma (OC) (**d**) tissues. The arrows indicate typical tissues (normal or cancer epithelium) stained with ECM1. Bars with 200 or 100 μm indicate the magnification of images. Silencing efficiency of ECM1 with specific shRNAs detected by WB (**e**), 3D culture image showing spheroid formation (**f**, bars = 400 µm) and numbers (**g**, data are presented as mean ± SD, *n* = 3 biologically independent repeats, two-tailed *t*-test), and xenograft tumor growth (**h**, data are presented as mean ± SD, *n* = 8v8 mice, two-tailed *t*-test) in eight mice generated from cells expressing ECM1 shRNA or control shRNA. Functional examination of ECM1 subtypes in HeyA8-ECM1 shRNA cells (A8i) after transfection of ECM1a (A8i-A), ECM1b (A8i-B), or empty vector (A8i-V), as confirmed by WB analysis (**i**); 3D culture images (**j**, bars = 400 µm) and spheroid number (**k**, data are presented as mean ± SD, *n* = 3 biologically independent repeats, two-tailed *t*-test); and assessment of xenograft tumor growth (**l**, data are presented as mean ± SD, *n* = 8v8 mice, two-tailed *t*-test) and tumor tissues (**m**) in eight mice injected with cells expressing ECM1a or ECM1b. **n** Alterations in signaling molecules associated with AKT/FAK/Paxillin/Rac after silencing of ECM1 and overexpression of ECM1a and ECM1b. Detection of the expression of phosphorylated proteins in cells treated or not treated with an HA antibody (**o**, A8i-A cells) or treated/not treated with bioactive ECM1 (ECM1a) (**p**, OVCA429 cells). PBS and mouse IgG were used as controls. β-actin was used as a loading control for WB analysis.

To investigate how ECM1 is regulated in cancer cells, we first examined ECM1 protein expression by quantitative reverse transcription (RT)-PCR (qRT-PCR) in seven human ovarian cancer cell lines and one normal human ovarian surface epithelial (HOSE) cell line. Low, high, and moderate ECM1 mRNA levels were detected in one (A2780), two (Hey and HeyA8), and four (SKOV3, SKOV3ip1, OVCA429 [429], and OVCA433 [433]) cancer cell lines, respectively, whereas no ECM1 mRNA was detected in HOSE cells (Fig. 1a). We also randomly selected three normal ovarian or fallopian tube tissues and ten high-grade serous epithelial ovarian carcinoma (OC) tissues from different patients and examined ECM1 expression by immunohistochemistry (IHC) with a commercial ECM1 antibody recognizing ECM1 isoforms a, b, and c. As shown in the representative images, ECM1 was not detected in normal ovarian or fallopian tube epithelia (FTE) but was strongly detected in OC samples by IHC (Fig. 1b–d). To identify the expression patterns of ECM1 isoforms in cell lines and tissues, we further performed semiquantitative RT-PCR, western blot (WB) analysis, and IHC using specific primers and custom antibodies to test each of the isoforms. We found that ECM1a mRNA was strongly expressed in the Hey and HeyA8 cell lines but weakly expressed in the other cancer cell lines and an immortalized FTE cell line, while it was undetectable in HOSE cells. In contrast, ECM1b and ECM1c mRNA expression was weakly detected in only the Hey and HeyA8 cell lines (Supplementary Fig. 1a, b). Only ECM1a protein was detected in both cell lysate (CL) and cell culture conditioned medium (CM) tested with antibodies specific to ECM1a and/or ECM1b/ECM1c proteins (Supplementary Fig. 1c). Although ECM1a and ECM1b proteins were detected by IHC in cancer tissues (Supplementary Fig. 1d, e), ECM1c was undetectable in all tested normal and cancer cell lines/tissues (Supplementary Fig. 1c, f). Thus, we focused mainly on the functions of ECM1a and ECM1b in the following studies. Nevertheless, the above data suggest that high expression of ECM1 may be associated with tumorigenesis.

To test the primary function of ECM1, we first silenced the expression of total ECM1 using short hairpin RNAs (shRNAs) specific for the transcripts encoding ECM1a, ECM1b, and ECM1c but not ECM1d (for rationale, see the “Materials and methods” section) (Fig. 1e) and analyzed the in vitro and in vivo tumor growth of the resulting cell lines. The silencing effects on the ECM1 isoforms were confirmed by semiquantitative RT-PCR and WB analysis using the specific primers and antibodies described above (Supplementary Fig. 1g–i). In three-dimensional (3D) culture experiments and animal assays, we found that the numbers of spheroids and growth of tumors were substantially lower for *ECM1*-silenced HeyA8 cells (HeyA8-ECM1i cells; hereafter labeled “A8i”, where “i” means interfering RNA, as in other labels) than for control cells (HeyA8-ctrli) (Fig. 1f–h).

To determine which subtype of ECM1 contributes to tumor growth, we delivered cDNAs encoding hemagglutinin (HA)-tagged ECM1a or ECM1b into A8i cells and established the A8i-A and A8i-B cell lines, respectively. We found that enforced overexpression of ECM1a, but not ECM1b, induced secretion, in vitro 3D growth and in vivo tumorigenicity (Fig. 1i–m and Supplementary Fig. 1j–l). Because ECM1 has been reported to regulate cytoskeletal signaling through activation of AKT/FAK/RHO signaling^7,17,18^, we investigated this pathway. We found that silencing of total ECM1 expression attenuated the phosphorylation of AKT (S473), FAK (Y397), Paxillin (Y118), and RAC (S71) (Fig. 1n) and that overexpression of ECM1a, but not ECM1b, markedly stimulated the expression of pAKT (S473), pFAK (Y397), pPaxillin (Y118), and pRac (S71) (Fig. 1n). Because ECM1 might function by being secreted and binding to putative cell surface receptors through autocrine signaling, we first blocked this signaling by treating A8i-A cells expressing HA-tagged ECM1a with an HA antibody to neutralize the secretion and function of ECM1a and found that the associated signaling molecules were strongly inhibited (Fig. 1o). Further tests after addition of bioactive recombinant ECM1 (ECM1a) into OVCA429 cells (low ECM1 expression) also proved that ECM1a induces the phosphorylation of AKT/FAK/Paxillin/Rac signaling molecules, most likely through ligand-receptor interactions (Fig. 1p).

To validate the above findings, we overexpressed ECM1a in the weakly tumorigenic OVCA429 and OVCA433 and highly tumorigenic SKOV3 ovarian cancer cell lines and in the immortalized ovarian surface epithelial cell line T29 and overexpressed ECM1b in SKOV3 and T29 cells. We found that ECM1a promoted spheroid formation in 3D culture, tumor growth in animals, and/or expression of phosphorylated AKT, FAK, Paxillin and Rac in SKOV3, OVCA429/OVCA433, and T29 cells (Supplementary Fig. 2). Similar results were found in the ovarian clear cell carcinoma cell line ES-2 and in an immortalized FTE cell line (Supplementary Fig. 3). All these data suggest that ECM1a, but not ECM1b, induces ovarian tumor growth, most likely through the AKT/FAK/Paxillin/Rac signaling axis.

### Identification of ECM1-associated molecules by RNA sequencing (RNA-seq)

To identify the molecules associated with ECM1-induced signaling and tumorigenesis, we performed RNA-seq. The RNA-seq data are presented as volcano plots, Gene Ontology (GO) enrichment results, Kyoto Encyclopedia of Genes and Genomes (KEGG) enrichment results, and heatmaps of gene expression values. There were 357, 277, and 177 significantly downregulated genes (green) and 404, 595, 343 significantly upregulated (red) genes in A8 vs. A8i cells, A8i-A vs. A8i cells, and A8i-A vs. A8i-B cells, respectively, as shown by volcano plots (Supplementary Fig. 4a). The GO enrichment analysis showed that these genes were significantly enriched for the receptor activity, ECM, and cell migration/proliferation/adhesion terms (Supplementary Fig. 4b). In addition, KEGG enrichment analysis revealed that the functions of these genes were associated with ovarian steroidogenesis, ECM-receptor interaction, cell adhesion molecules, and ATP binding cassette (ABC) transporters (Supplementary Fig. 4c and Supplementary Data 1–3). Subsequent analyses showed that integrins, heterogeneous nuclear ribonucleoproteins (hnRNPs), and ABC transporters were highly altered by ECM1 silencing and ECM1a overexpression (Supplementary Fig. 5a, c, e). Further tests by real-time RT-PCR revealed that the mRNA levels of integrins αX and β2, hnRNPLL, and ABCG1 were significantly reduced in ECM1-silenced cells and ECM1b-overexpressing cells but were high in parental and ECM1a-overexpressing cells (Supplementary Fig. 5b, d, f). Therefore, we focused mainly on the functions of these molecules in subsequent studies.

### Interaction between integrin αXβ2 and the GPR motif of ECM1a determines tumorigenesis

**Fig. 2:**
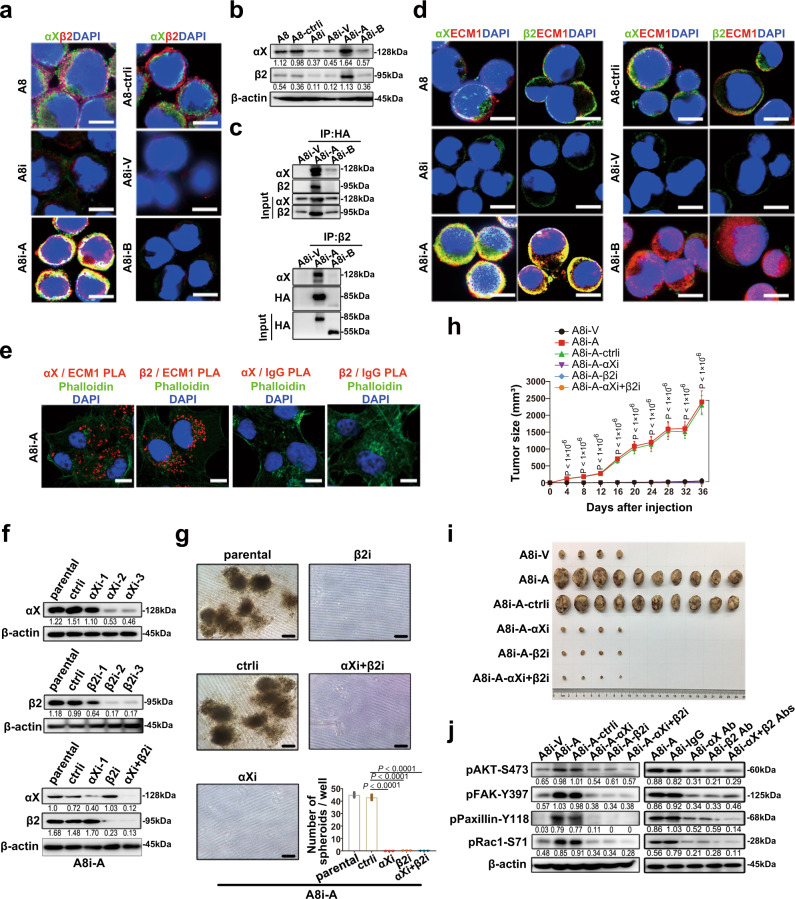
Interaction of ECM1a with integrin αXβ2. Protein colocalization or expression of integrins αX and β2 in A8, A8-ctrli, A8i, A8i-V, A8i-A, and A8i-B cells as detected by IF (**a**, bars = 10 µm) or WB analysis (**b**). Interactive binding of ECM1a or ECM1b with integrin αX and/or integrin β2 as detected by co-IP (**c**), IF (**d**), and PLA (**e**). Bars = 10 µm. Silencing of integrin *αX* and/or integrin *β2* with specific shRNAs (**f**) altered in vitro cell 3D growth (**g**, bars = 400 μm, quantification data are presented as mean ± SD, *n* = 3 biologically independent repeats, two-tailed *t*-test), tumor growth (**h**, data are presented as mean ± SD, *n* = 10v10 mice, two-tailed *t*-test) and tissues (**i**) in ten mice. **j** Detection of cellular signaling molecules associated with ECM1 in cells expressing shRNAs against integrin αX, β2, or αXβ2 (left panel) or in cells pretreated with antibodies against integrin αX, β2, or αXβ2 (right panel).

Based on the RNA-seq analysis, we further tested integrin αXβ2 expression and localization and found that integrin αXβ2 expression was downregulated when ECM1 was silenced or when ECM1b was overexpressed but upregulated in ECM1a-overexpressing cells (Fig. 2a, b). Because integrins are potential ECM receptors, we performed coimmunoprecipitation (co-IP) and immunofluorescence (IF) assays and found that both integrins αX and β2, bound to ECM1a but not to ECM1b (Fig. 2c, d), whereas ECM1b was localized in the cytoplasm (Fig. 2d). A proximity ligation assay (PLA) also showed that only ECM1a bound to integrins αX and β2 (Fig. 2e). When integrin αX, integrin β2, or integrin αX/β2 was stably silenced (Fig. 2f) in ECM1a-overexpressing cells, in vitro cell growth in 3D culture and in vivo tumor growth in animals were strongly inhibited (Fig. 2g–i). The levels of the signaling molecules pAKT, pFAK, pPaxillin, and pRac were also reduced by silencing of integrin αX, integrin β2, or integrin αX/β2 (Fig. 2j, left panel) or by treatment of the cells (A8i-A) with antibodies against integrin αX, integrin β2, or integrin αX/β2 (Fig. 2j, right panel). Moreover, we performed co-IP assays using cell culture supernatants collected from HeyA8 cells overexpressing ECM1a or ECM1b (with HA-tag) and found that ECM1a, but not ECM1b, was detectable in the CM; in addition, the binding of ECM1a (HA-tag), but not ECM1b, with integrin αX or β2 was clearly shown (Supplementary Fig. 6a, b). These data suggest that integrin αXβ2 functions as a putative receptor to mediate mainly ECM1-associated signaling and that the functional interaction of ECM1 with integrin αXβ2 depends on the ECM1a subtype rather than the ECM1b subtype.

**Fig. 3:**
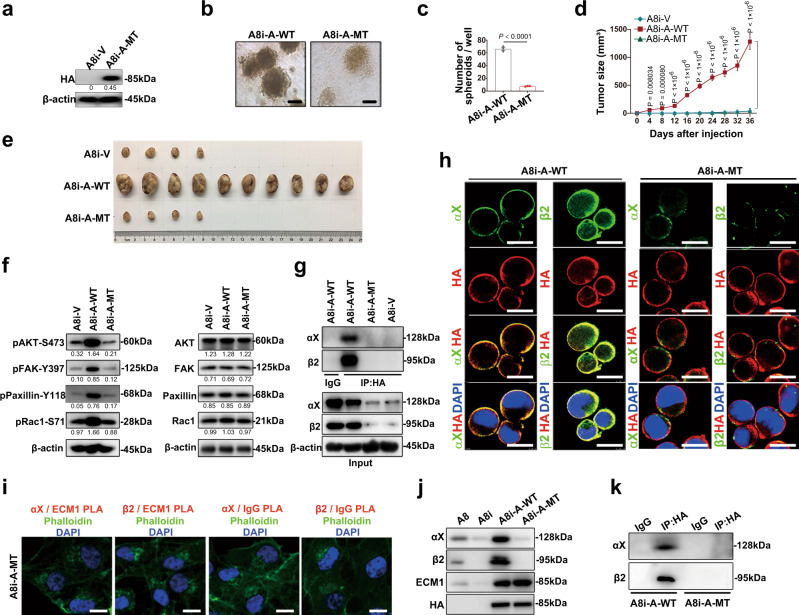
The ECM1a GPR motif determines signaling and tumorigenesis. **a** Detection of HA-tagged ECM1a-MT expression in an established cell line by WB analysis. 3D cell growth (**b**, bars = 400 µm) and quantification (**c**, data are presented as mean ± SD, *n* = 3 biologically independent repeats, two-tailed *t*-test) induced by ECM1a-MT or ECM1a-WT. Xenograft tumor growth (**d**, data are presented as mean ± SD, *n* = 10v10 mice, two-tailed *t*-test) and tissues (**e**) in ten mice and cellular signaling molecules (**f**, the actin loading control in **f** left panel was also used for the same samples in Fig. 4g because these tests were parallelly performed) were inhibited by ECM1a-MT. **g** Loss of direct binding of integrin αX or β2 to ECM1a-MT, in contrast with the binding to ECM1a-WT, as tested by co-IP. Cellular colocalization of integrin αX or β2 with ECM1a-WT or ECM1a-MT as detected by IF (**h**, bars = 10 µm) and PLA (**i**, bars = 10 µm). Detection of integrins αX and β2 and of WT and MT ECM1 by WB analysis of cell membrane extracts (**j**) and detection of integrins αX and β2 (**k**) by co-IP/WB analysis after cell surface labeling with biotinylation reagents.

Because a study has reported that a GPR domain identified in the α-chain of fibrinogen may bind to integrin αXβ2^19^ and because the GPR motif is also found in ECM1, we tested whether the GPR motif in ECM1a plays a key role in the interaction between ECM1a and integrin αXβ2. We constructed an ECM1a mutant (MT) with the VAQ sequence (mutated from ggtccccga <GPR> to gttgcccaa <VAQ>), and we found that cells transfected with ECM1a-MT had lower growth in 3D culture and xenograft animal models (Fig. 3a–e) and lower expression of phosphorylated AKT, FAK, Paxillin, and Rac than cells transfected with ECM1a-WT (Fig. 3f). In addition, in ECM1a-MT-expressing cells, the binding of ECM1a-MT to integrin αXβ2 was lost, as determined by co-IP/WB (Fig. 3g), IF (Fig. 3h), PLA (Fig. 3i), and cell surface biotinylation (Fig. 3j, k) assays, although ECM1a-MT still bound to the cell surface membrane (Fig. 3h, j). These data imply that the GPR motif of ECM1a is functionally necessary for the signaling and tumor growth induced by the interaction between ECM1a and integrin αXβ2, whereas integrins αX and β2 are not the only molecules on the cell surface that bind to ECM1a. To further investigate other potential cell surface molecules that may bind to ECM1a-WT and ECM1a-MT, we performed co-IP assays using an HA antibody and the extracts of total CL or membrane fractions from ECM1a-WT- or ECM1a-MT-expressing cells or parental HeyA8 cells treated with the CM of ECM1a-WT- or MT-expressing cells (Supplementary Fig. 6c–e). The data analyzed from the selected samples by mass spectrometry (MS) and co-IP assays showed that integrin β1, JUP, annexin A2, calnexin, and S100A9 might also bind to ECM1a-WT (Supplementary Fig. 6f–j and Supplementary Table 1), whereas plakophilin-1 and S100A8 could bind to ECM1a-MT (Supplementary Fig. 6k, l). These data indicate that the binding of integrin αXβ2 to ECM1a may not cover all ECM1 binding sites to prevent access by other cell surface molecules, including integrin β1, JUP, annexin A2, calnexin, and S100A9, although it may competitively inhibit the binding of ECM1a to plakophilin-1 and S100A8.

Loike et al. reported that CD11c/CD18 (integrin αXβ2) on tumor necrosis factor-stimulated neutrophils may function as a fibrinogen receptor by binding with the GPR sequence in the fibrinogen α chain (FGA)^19^, whereas the fibrinogen γ chain (FGG) may also be recognized by integrin αXβ2 on leukocytes^20,21^. Two different studies have found that ICAM-1 may be recognized by αX or β2 of αXβ2^22,23^, and iC3b has been reported to bind with the αX subunit of integrin αXβ2 on erythrocytes^24^, on neutrophils and monocytes^25^ and on alveolar macrophages^26^. However, additional studies have reported that the αX I-domain of αXβ2 may also bind to plasminogen (PLG)^27^, CD90 (Thy-1)^28^, or heparin/PF4^29^. Therefore, we first analyzed the expression levels of these ligands in A8 (high ECM1 and αXβ2), A8i (low ECM1 and αXβ2), and A8i-A (high ECM1a and αXβ2) cells by WB analysis based on the available RNA-seq data (Supplementary Table 2). We found that the expression of FGG, ICAM1, PLG, and CD90 appeared lower in A8i cells than in A8 and A8i-A cells. The expression of FGA and iC3b in A8i cells was higher than that in A8 cells but lower than that in A8i-A cells, whereas the expression of heparin/PF4 in A8i cells was higher than that in both A8 and A8i-A cells. These findings suggest that the interaction between ECM1 and αXβ2 may facilitate the expression of FGG, ICAM1, PLG, and CD90 but may not be associated with the expression of FGA and iC3b. The findings also suggest that the expression of PF4 is inversely correlated with the expression of ECM1 and αXβ2 (Supplementary Fig. 7a).

Next, we performed co-IP with antibodies against αX or β2 and found that αX could bind to all of the ligands, whereas β2 bound to FGG only (Supplementary Fig. 7b). The degrees of binding of FGA, FGG, PLG, iC3b, and CD90 to αX were correlated with the expression levels of these ligands detected in the corresponding CLs, but the degrees of binding of ICAM1 and heparin/PF to αX were not correlated with the expression levels of these molecules in cell lines (Supplementary Fig. 7b, left panel). In addition, the degree of binding of FGG to β2 was different from FGG expression in cell lines (Supplementary Fig. 7b, right panel). However, the data obtained from the reverse co-IP products using antibodies against the ligands showed that the degrees of binding of αX to FGA, FGG, ICAM1, PLG, and CD90 were higher in A8i cells than in A8 and A8i-A cells and that the degrees of binding of αX to iC3b and PF4 were consistent with those of iC3b and PF4 to αX. The degree of binding of β2 to FGG was inversely correlated with that of FGG to β2 (Supplementary Fig. 7b and Supplementary Fig. 7c). These data suggest that the binding of ECM1a to αXβ2 may differentially regulate the functions of FGA, FGG, ICAM1, PLG, iC3b, CD90, and PF4 and the abilities of these ligands to bind to integrin αXβ2. However, more detailed investigations are needed for each of these ligands because they may also functionally bind to molecules other than αX and β2.

### Nonsecretory ECM1b inhibits tumorigenesis by blocking myosin phosphorylation

**Fig. 4:**
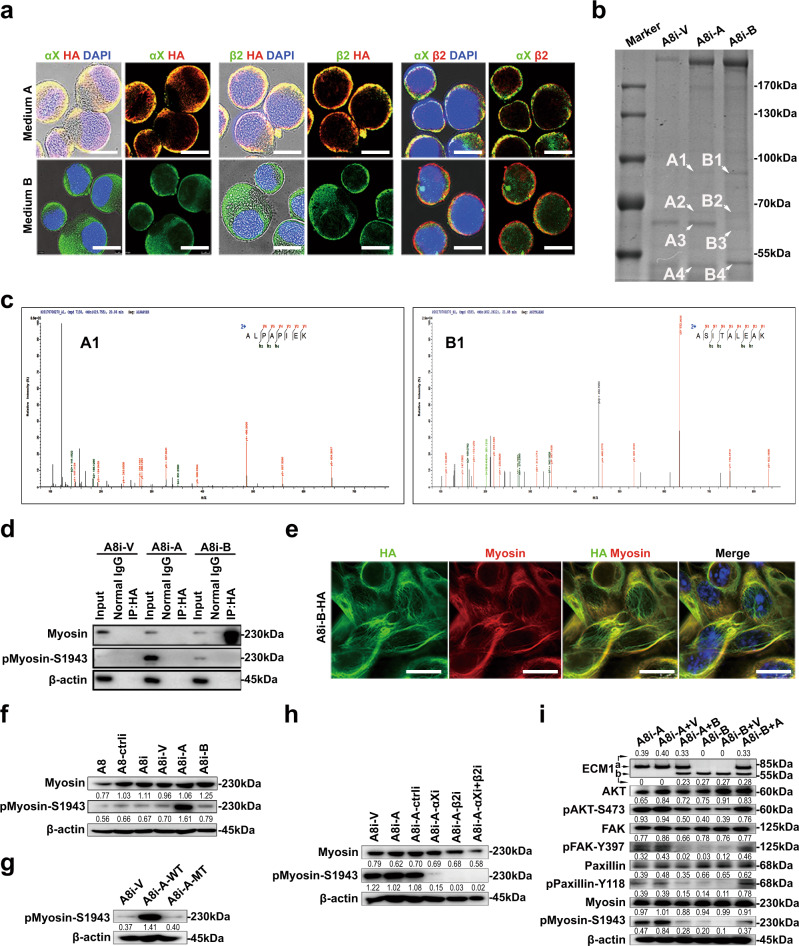
Nonsecretory ECM1b inhibits myosin phosphorylation and tumor growth. **a** Colocalization of integrin αX or β2 with HA (ECM1a/1b) in HeyA8 cells treated with medium A and B collected from A8i-A and A8i-B cells, respectively (bars = 10 µm). **b** Coomassie Brilliant Blue (R-250) staining of co-IP products prepared with cell lysates (CLs) of A8i-A and A8i-B cells and an anti-HA antibody after separation by SDS-PAGE; the arrows indicate the protein bands analyzed by mass spectrum (MS). **c** Results of secondary ion MS analyses of the most abundant proteins in A1 and B1 samples derived from ECM1a and ECM1b, respectively. **d** Direct binding of ECM1a or ECM1b to nonphosphorylated and phosphorylated myosin IIa detected by co-IP and WB analysis using corresponding antibodies. **e** Colocalization of ECM1b with nonphosphorylated myosin IIa detected by IF in cells (bar = 10 µm). Expression of myosin IIa and phosphorylated myosin IIa detected by WB analysis in ECM1-silenced cells and ECM1a- and ECM1b-overexpressing cells (**f**), in WT and MT ECM1-overexpressing cells (**g**, the actin loading control in **g** was also used for the same samples in Fig. 3f left panel because these tests were parallelly performed), in integrin αX- or integrin β2-knockdown cells (**h**), and in ECM1a cells (A8i-A) transfected with ECM1b (A8i-A + B) or ECM1b cells (A8i-B) transfected with ECM1a (A8i-B + A) (**i**).

Based on the finding that HA-ECM1b was localized to the cytoplasm (Fig. 2d) and undetectable in the cell culture medium, unlike HA-ECM1a (Fig. 1i, lower panel), we further treated HeyA8 cells with medium collected from cells expressing ECM1a or ECM1b. We failed to detect the colocalization of integrin αX or β2 with HA-tagged ECM1b, in contrast to the findings obtained with HA-ECM1a (Fig. 4a). These results suggest that there may be factors in the cytoplasm that interact with and retain ECM1b to prevent its secretion from cells.

To assess this possibility, we performed co-IP assays with an HA-specific antibody and lysates from ECM1a- and ECM1b-expressing cells. Gel analysis showed the presence of ECM1a- and ECM1b-specific bands after co-IP of the various HA-tagged proteins (Fig. 4b). Analysis of the abundance of these proteins by MS showed that myosin was one of the predominantly enriched proteins in ECM1b samples compared with ECM1a samples (Fig. 4c and Supplementary Data 4–5). By co-IP and immunoblotting, we further showed that ECM1b interacted only with nonphosphorylated myosin (Fig. 4d), which was confirmed by IF (Fig. 4e). Subsequently, we found that the phosphorylation of myosin (S1943) was highly activated only by ECM1a-WT; it was repressed by ECM1b or ECM1a-MT overexpression and by silencing of integrin *αX* and/or integrin *β2* (Fig. 4f–h). Because the above data reveal that ECM1a and ECM1b have opposite effects on tumorigenesis, we further delivered ECM1b into parental HeyA8 cells highly expressing ECM1a. The data showed that ECM1b inhibited the phosphorylation of AKT/FAK/Paxillin/Rac and the tumor growth of cells in animals (Supplementary Fig. 8a–c). On the other hand, we found that silencing the expression of ECM1a and ECM1c with specific shRNAs enhanced the expression of ECM1b at both the mRNA and protein levels but blocked the tumor growth of cells (Supplementary Fig. 8d–h). We further transfected ECM1b or ECM1a into ECM1a- or ECM1b-overexpressing cells. Compared with parental cells or vector-transfected control cells, cells transfected with ECM1b or ECM1a exhibited reduced or enhanced phosphorylated AKT/FAK/Paxillin/Myosin protein levels and xenograft tumor development, respectively (Fig. 4i and Supplementary Fig. 8i, j). These data imply that the phosphorylation of AKT/FAK/Paxillin/Myosin is essential for the signaling and tumorigenesis induced by the ECM1a-integrin αXβ2 interaction.

### hnRNPLL regulates ECM1 mRNA splicing

**Fig. 5:**
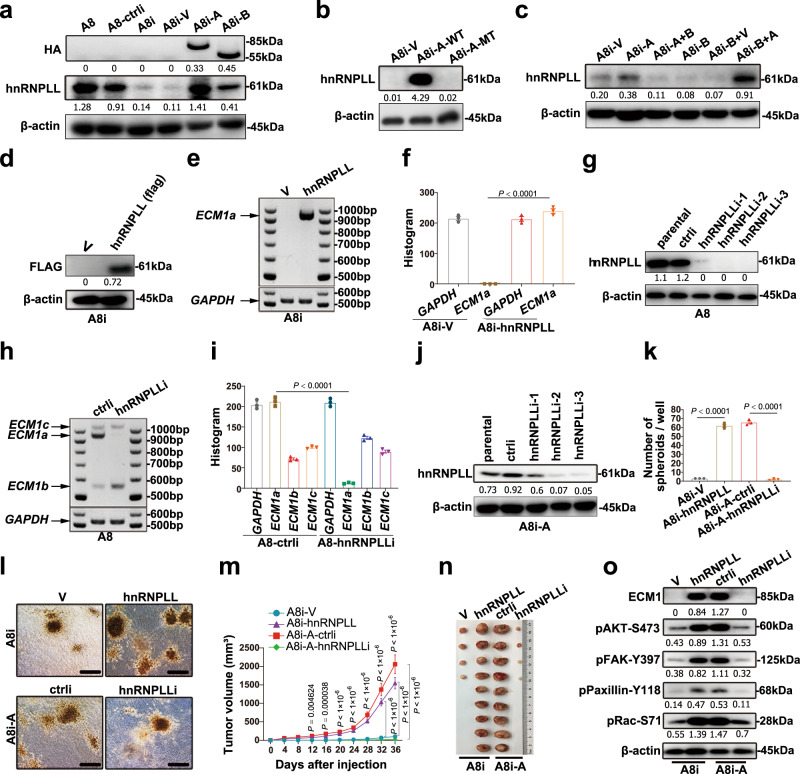
hnRNPLL determines ECM1a mRNA splicing and ECM1a-associated signaling and tumor growth. *hnRNPLL* protein expression in ECM1-silenced and ECM1a-overexpressing cells (**a**), in ECM1a-WT/ECM1a-MT cells (**b**), and in ECM1a- and ECM1b-cross-transfected cells (**c**). Overexpression of hnRNPLL in A8i cells (**d**) promotes ECM1a mRNA splicing, as tested by semiquantitative RT-PCR (**e**) and quantification (**f**, data are presented as mean ± SD, *n* = 3 biologically independent repeats, two-tailed *t*-test). Silencing of hnRNPLL in A8 cells with specific shRNAs (**g**) reduces ECM1a mRNA production but upregulates ECM1b mRNA production, as detected by semiquantitative RT-PCR (**h**) and quantification (**i**, data are presented as mean ± SD, *n* = 3 biologically independent repeats, two-tailed *t*-test). hnRNPLL silencing (**j**) or overexpression inhibits or enhances 3D cell growth as shown by spheroid quantification (**k**, data are presented as mean ± SD, *n* = 3 biologically independent repeats, two-tailed *t*-test) and formation (**l**, bars = 400 μm), xenograft tumor growth (**m**, data are presented as mean ± SD, *n* = 9v9 mice, two-tailed *t*-test) and tissues (**n**) in nine mice, and activation of ECM1-associated signaling molecules (**o**), respectively.

Based on the RNA-seq data and qRT-PCR results (Supplementary Fig. 5c, d), we further found that the protein expression of *hnRNPLL* was strongly induced by ECM1a but not by ECM1b (Fig. 5a). Mutation of ECM1a and transfection of ECM1b or ECM1a into ECM1a- or ECM1b-overexpressing cells also resulted in decreased or increased expression of hnRNPLL (Fig. 5b, c), suggesting that hnRNPLL is regulated by ECM1a.

We next overexpressed hnRNPLL in A8i, T29, and ES-2 cells with *hnRNPLL* cDNA and silenced *hnRNPLL* in A8 and A8i-A cells with specific shRNAs against *hnRNPLL*. Semiquantitative RT-PCR revealed that overexpression of hnRNPLL favored *ECM1a* over *ECM1b* and *ECM1c* mRNA production (Fig. 5d–f and Supplementary Fig. 9a–f), whereas silencing of hnRNPLL expression diminished the generation of *ECM1a* mRNA (Fig. 5g–i). Delivery of an *hnRNPLL*-specific shRNA into A8i-A cells or transfection of *hnRNPLL* cDNA into A8i cells repressed or enhanced 3D culture growth, tumor growth, and ECM1a, pAKT, pFAK, pPaxillin and pRac expression, respectively (Fig. 5j–o). These data suggest that ECM1a positively regulates hnRNPLL, whereas hnRNPLL, in return, strengthens ECM1a protein expression by favoring ECM1a mRNA splicing to facilitate cytoskeletal signaling.

Because hnRNPs usually act as splicing repressors, we further tested the mRNA and protein expression levels of *hnRNPL*, *hnRNPM*, and *hnRNPU* in ECM1-silenced and ECM1a- or ECM1b-overexpressing cells based on RNA-seq data (Supplementary Fig. 5c). The experiments showed that ECM1a enhanced the expression of these genes, whereas ECM1b did not (Supplementary Fig. 10a–d). However, silencing of these genes with siRNAs failed to induce altered splicing of ECM1 isoforms (Supplementary Fig. 10e–m). To test whether hnRNPLL directly regulates the mRNA stability of ECM1 isoforms, total RNA was isolated from HeyA8 cells transfected with either control shRNA or hnRNPLL shRNA at 35% (12 h), 50% (24 h), 75% (36 h), or 90% (48 h) confluence and used to perform semiquantitative RT-PCR assays. The results showed that the mRNA level of each isoform was highly stable without regard to cell differentiation, suggesting that hnRNPLL regulates the expression of ECM1 isoforms most likely through alternative mRNA splicing rather than through regulation of mRNA stability (Supplementary Fig. 11a, b). We then analyzed the expression of Serine/Arginine Splicing Factors (SRSFs), the counterfactors of hnRNPs, which function as splicing activators. The data showed that the levels of SRSF1 and SRSF6 were elevated in ECM1b- and ECM1a-overexpressing cells, respectively (Supplementary Fig. 11c–f) and that overexpression of SRSF1 enhanced ECM1b mRNA splicing, whereas silencing of SRSF6 reduced ECM1a splicing (Supplementary Fig. 11g–l). These results suggest that hnRNPLL, SRSF1, and SRSF6 actively participate in the alternative mRNA splicing of ECM1 isoforms.

### ABCG1 mediates ECM1a-associated signaling and tumorigenesis

**Fig. 6:**
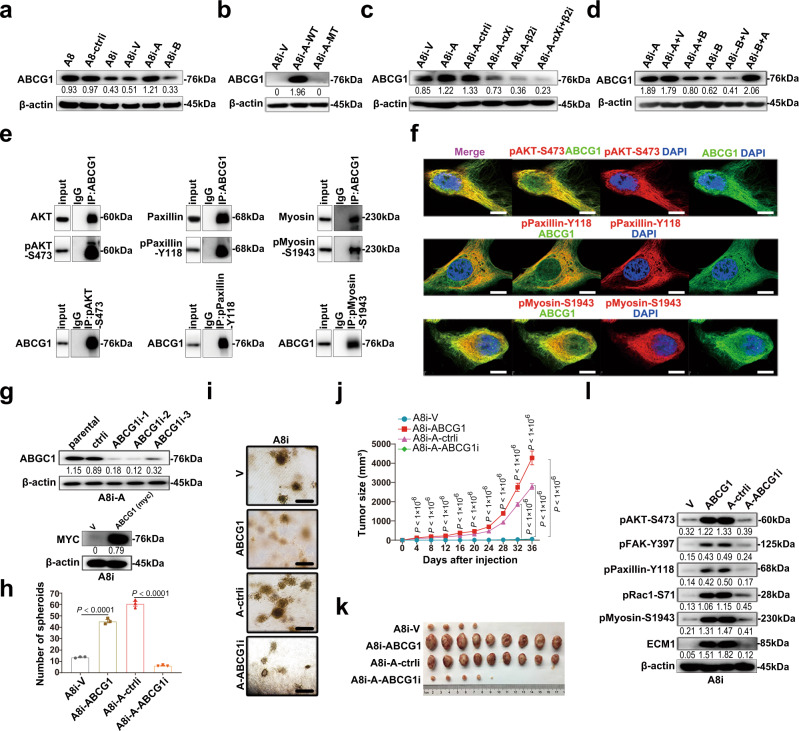
ABCG1 mediates ECM1a-induced cellular signaling and tumorigenesis. Detection of *ABCG1* protein expression by WB analysis in ECM1-silenced/overexpressing cells (**a**), in ECM1a-WT/ECM1a-MT-expressing cells (**b**), in integrin αXβ2-silenced cells (**c**), and in ECM1b- or ECM1a-cross-transfected cells (**d**). Direct binding and colocalization between ABCG1 and AKT/pAKT, paxillin/pPaxillin, or myosin/pMyosin as detected by co-IP/WB analysis (**e**) and IF (**f**, bars = 10 μm). Stable silencing or overexpression of ABCG1 (**g**) inhibited or promoted the number (**h**, data are presented as mean ± SD, *n* = 3 biologically independent repeats, two-tailed *t*-test) and growth (**i**, bars = 400 μm) of 3D culture spheroids, xenograft tumor growth (**j**, data are presented as mean ± SD, *n* = 9v9 mice, two-tailed *t*-test) and tumor tissues (**k**) in nine mice injected with related cells, and cellular signaling molecules associated with ECM1a and integrin αXβ2 (**l**).

Based on the RNA-seq and qRT-PCR analyses in Supplementary Fig. 5e, f, we examined the expression of ABCG1 in various cell lines and found that ABCG1 expression was higher in parental and ECM1a-overexpressing cells (A8i-A) than in ECM1-silenced (A8i) or ECM1b-overexpressing cells (A8i-B) (Fig. 6a). ABCG1 was reduced by ECM1a mutation (A8i-A-MT) (Fig. 6b) or by silencing of integrin αX and/or integrin β2 (Fig. 6c). ABCG1 expression was attenuated by delivery of ECM1b into ECM1a-overexpressing cells and enhanced by transfection of ECM1a into ECM1b-overexpressing cells (Fig. 6d).

Because the above data showed that ECM1-induced cellular signaling is associated mainly with AKT/FAK/Paxillin/myosin, we tested whether ABCG1 directly interacts with these proteins. Using co-IP and IF, we observed a strong interaction between ABCG1 and phosphorylated or nonphosphorylated AKT, Paxillin, and myosin (Fig. 6e, f). To test the role of ABCG1 in ECM1- and integrin αXβ2-induced in vitro 3D growth and in vivo tumor growth, we silenced or overexpressed *ABCG1* expression in A8i-A or A8i cells, respectively (Fig. 6g), and found that the number of spheroids in 3D culture, the growth of tumors, and the levels of phosphorylated AKT, FAK, Paxillin, Rac, and Myosin were markedly reduced (Fig. 6h–l). These data indicate that ABCG1 may mediate the ECM1a-integrin αXβ2 interactive signaling to control tumorigenesis through regulation of AKT/FAK/Paxillin/Rac/Myosin protein phosphorylation.

To investigate ABCG1-mediated phosphorylation signaling, we first performed an in vitro kinase assay using ABCG1 as a putative kinase and AKT2 as a substrate, although no evidence suggests that ABCG1 is a kinase. Surprisingly, our data demonstrated that ABCG1 indeed phosphorylated AKT2-WT at S474 (corresponding to AKT1 S473) and that this phosphorylation was suppressed by MK-2206, a specific inhibitor of AKT serine phosphorylation (Supplementary Fig. 12a, b). Since protein phosphorylation is also regulated by protein phosphatases and phosphatase inhibitors, we analyzed the expression of serine/threonine-protein phosphatase and inhibitor genes by qRT-PCR and WB analysis based on the RNA-seq data. As shown in Supplementary Fig. 12c–e, protein phosphatase 2A catalytic subunit β (PPP2CB) expression was downregulated in ECM1a-overexpressing cells but was upregulated in ECM1b-overexpressing cells, whereas protein phosphatase 1 regulatory inhibitor subunit 14C (PPP1R14C) expression was increased in ECM1a-overexpressing cells but was reduced in ECM1b-overexpressing cells. Treatment of A8i cells with okadaic acid, a specific inhibitor of protein phosphatases 1 and 2A, apparently stimulated the protein phosphorylation of AKT/Rac/Myosin (Supplementary Fig. 12f, left panel), whereas treatment of A8i-A cells with ceramide, an activator of protein phosphatases 1 and 2A, suppressed the phosphorylation of AKT/Rac/Myosin proteins (Supplementary Fig. 12f, right panel). These findings suggest that the ECM1-induced phosphorylation of AKT/Rac/Myosin may be directly regulated by ABCG1 and/or by serine/threonine-protein phosphatases and inhibitors such as PPP2CB and PP1R14C. However, the tyrosine phosphorylation of FAK and Paxillin needs further investigation.

Because many studies have reported that ABCG1 regulates intracellular cholesterol homeostasis mainly by affecting cholesterol efflux, we also tested the total cholesterol levels and cholesterol efflux in ovarian cancer cells overexpressing ECM1a, ECM1b, ABCG1, and hnRNPLL. We found that both cholesterol efflux and total cholesterol levels were decreased by ECM1 silencing or ECM1b overexpression but were increased by overexpression of ECM1a, ABCG1, or hnRNPLL (Supplementary Fig. 12g).

### ABCG1 confers cancer cell stemness and cisplatin resistance through upregulation of CD326

**Fig. 7:**
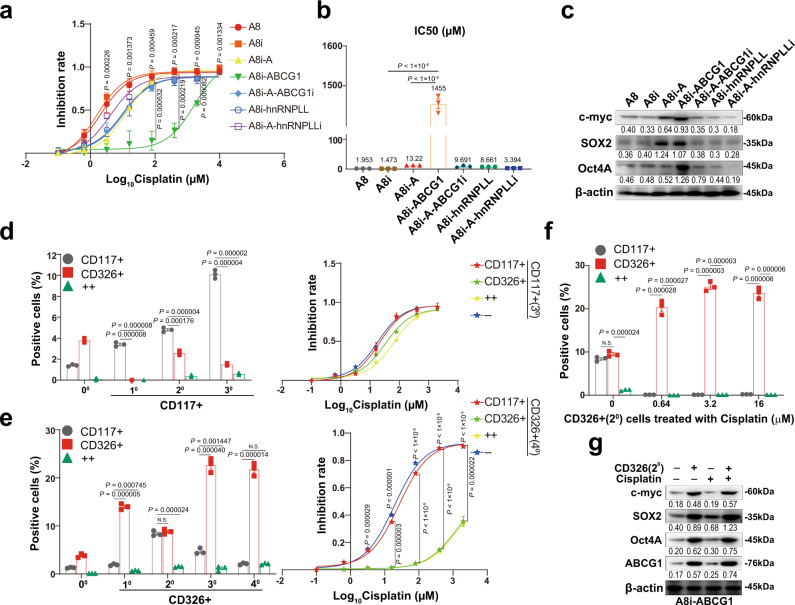
ABCG1 confers cancer cell stemness and cisplatin resistance through upregulation of CD326. Inhibition rates of various cell lines treated with cisplatin in terms of silencing or overexpression of ECM1, ABCG1, and hnRNPLL (**a**, data are presented as mean ± SD, *n* = 3 biologically independent repeats, two-tailed *t*-test was calculated between A8i-ABCG1 and A8i <upper> or A8i-A <lower>) and IC50 values (**b**, data are presented as mean ± SD, *n* = 3 biologically independent repeats, two-tailed *t*-test) of cisplatin in these cells as detected by CCK-8 assay. **c** Expression of stem cell transcription factors detected by WB analysis in ECM1-, ABCG1-, and hnRNPLL-silenced or -overexpressing cells. **d** Numbers of CD117+ and CD326+ cells (left panel, data are presented as ± SD, n = 3 biologically independent repeats, two-tailed *t*-test) and cisplatin inhibition rates (right panel, data are presented as ± SD, *n* = 3 biologically independent repeats) after three selection-and-culture cycles from CD117 + cells. **e** Numbers of CD117 + and CD326 + cells (left panel, data are presented as mean ± SD, *n* = 3 biologically independent repeats, two-tailed *t*-test) and cisplatin inhibition rates (right panel, data are presented as mean ± SD, *n* = 3 biologically independent repeats, two-tailed *t*-test was calculated between CD117+ and CD326+ <upper> or CD117+/CD326+ and CD117−/CD326−) after four selection-and-culture cycles from CD326+ cells. **f** A low concentration of cisplatin enriches CD326+ cells to increase cancer cell stemness. Data are presented as mean ± SD, *n* = 3 biologically independent repeats, two-tailed *t*-test. **g** Cisplatin treatment upregulates the expression of stem cell transcription factors in CD326+ cells.

Because one of the biggest obstacles in the treatment of most cancers, including ovarian cancer, is platinum resistance^30,31^, we first examined the cisplatin sensitivity of the ECM1-associated cell lines established in this study. As shown in Fig. 7a, b, cells expressing ABCG1 appeared to have the lowest cisplatin inhibition rate among all cell lines, and the IC50 values of cells expressing ABCG1, ECM1a, and hnRNPLL were 1455, 13.22, and 8.661 μM, respectively, indicating that ABCG1 is a key factor driving cisplatin resistance.

Since chemoresistance is associated with cancer cell stemness^32,33^, we tested some stem cell transcription factors and found that Oct4A was particularly upregulated by ABCG1 overexpression, whereas c-Myc and SOX2 were upregulated by both ECM1a and ABCG1 (Fig. 7c). Thus, we focused on the stemness of cancer cells induced by ABCG1. Flow cytometric analyses showed that the percentages of CD24-, CD133-, CD117-, and CD326-positive cells expressing ABCG1 were 0.5%, 0.1%, 1.4%, and 5.4%, respectively (Supplementary Fig. 13a–d), indicating that ABCG1 enriches the populations of CD117+ and CD326+ cells. Further tests showed that the inhibition rate of CD326+ cells treated with cisplatin was strongly reduced, whereas that of CD117+ cells was slightly decreased compared with that of CD326− or CD117− cells (Supplementary Fig. 13e). Thus, we recultured the cells derived from the first or the second selection of CD117+ or CD326+ cells with serum-free medium. Analysis of CD117+ cells after three culture-and-selection cycles showed that CD117−/CD326− and CD117+ cells were more sensitive to cisplatin treatment than CD117+/CD326+ and CD326+ cells, although CD117+ cells accounted for 10.1% (Fig. 7d and Supplementary Fig. 14a–d). However, examination of CD326+ cells after four culture-and-selection cycles suggested that CD326+ and CD117+/CD326+ cells were much more resistant to cisplatin than CD117−/CD326− and CD117+ cells (Fig. 7e and Supplementary Fig. 14e–i). These data suggest that CD326 may confer cancer cell stemness on ABCG1-overexpressing cells, which is also supported by the data from ES-2 cells (Supplementary Fig. 14j–l).

Because studies have shown that cancer cell stemness may be enhanced by treatment with a low concentration of cisplatin^34–36^, we treated the second set of selected CD326+, CD117+, or CD117+/CD326+ cells derived from CD326+ cells with cisplatin at concentrations of 0.64, 3.2, and 16 μM and found that the number of CD326+ cells was highly increased by cisplatin treatment (Fig. 7f and Supplementary Fig. 14m). Further analysis of the stemness of CD326+ cells treated with cisplatin at 3.2 μM revealed that the expression of c-Myc, Oct4A, and SOX2 was upregulated in both CD326+ and cisplatin-treated CD326+ cells with ABCG1 overexpression (Fig. 7g). These data suggest that ECM1 induces cancer cell stemness to confer cisplatin resistance through ABCG1-mediated upregulation of CD326, whereas treatment of CD326+ cells with a low dose of cisplatin significantly enriches the CD326+ cell population to enhance cancer cell stemness.

### Clinical correlations and association of patient survival with the expression of ECM1 subtypes, integrin αXβ2, hnRNPLL, and ABCG1 in ovarian cancer tissues

**Fig. 8:**
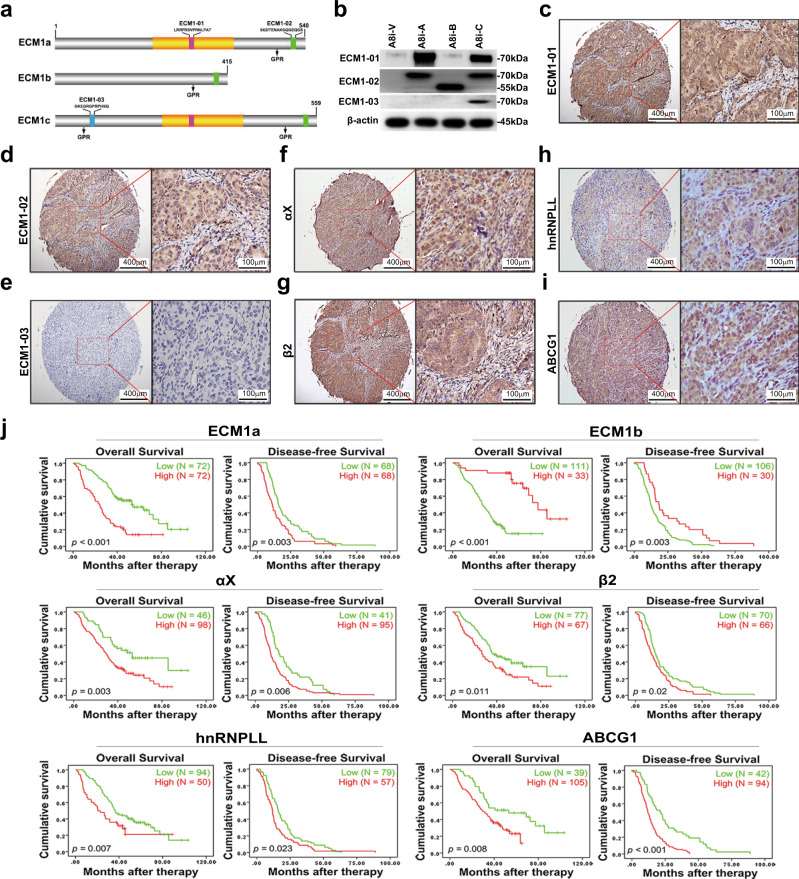
Correlations of ECM1a, ECM1b, integrin αX, integrin β2, and hnRNPLL levels with cancer patient survival. Design of antibodies recognizing ECM1 subtypes (**a**) and detection of ECM1 subtypes in HeyA8 cells expressing ECM1 shRNA and further transfected with vector (A8i-V), ECM1a (A8i-A), ECM1b (A8i-B), or ECM1c (A8i-C) cDNAs by WB analysis with specific antibodies (**b**). **c**–**i** Representative images from the TMA. IHC showed different levels of ECM1a (**c**, detected with the ECM1-01 antibody), ECM1a or ECM1b (**d**, detected with the ECM1-02 antibody), ECM1c (**e**, detected with the ECM1-03 antibody), integrin αX (**f**), integrin β2 (**g**), hnRNPLL (**h**), and ABCG1 (**i**); bars = 400 or 100 μm indicating different magnifications of the tissues. **j** A TMA consisting of 150 high-grade serous OC tissues and 30 normal ovarian tissues was stained by IHC for analysis of ECM1 subtypes, integrin αX, integrin β2, hnRNPLL, and ABCG1 expression. The level of each protein was classified as high or low expression. Survival analysis conducted with Kaplan–Meier plots showed that poor OS and DFS were associated with high expression of ECM1a, integrin αX, integrin β2, ABCG1, and hnRNPLL, whereas favorable OS and DFS were associated with high expression of ECM1b. Two-tailed *t*-test was used for survival analyses.

To validate the clinical significance of the aforementioned molecules, IHC was used to analyze the expression of ECM1a, ECM1b, ECM1c, integrin αX, integrin β2, hnRNPLL, and ABCG1 in a tissue microarray (TMA) consisting of 150 high-grade serous OC samples and 30 normal ovary samples from different subjects. Because commercial antibodies against ECM1 could not be used for IHC to identify the expression differences among ECM1 subtypes in cancer tissues, we ordered three custom rabbit polyclonal antibodies to identify the protein expression differences of the ECM1 subtypes. As indicated in Fig. 8a, an ECM1-E03 antibody was generated to specifically detect ECM1c expression, an ECM1-E01 antibody was used to detect both ECM1a and ECM1c expression, and an ECM1-E02 antibody was used to detect the expression of all of the subtypes. Thus, ECM1a and ECM1b were able to be detected by ECM1-01^+^/ECM1-03^−^ and ECM1-02^+^/ECM1-01^−^/ECM1-03^−^, respectively. The specificity of these antibodies in cancer cell lines was confirmed by WB analysis (Fig. 8b) and prestaining of a few cancer tissues (Supplementary Fig. 1d–f) before TMA staining. All normal tissues were negatively stained with these antibodies. While cancer tissues could be positively stained with ECM-01 and ECM1-02 antibodies (Fig. 8c, d), we did not observe ECM1c expression in any cancer tissues with the E03 antibody, indicating that ECM1c was expressed at low levels in both ovarian cancer tissues and normal tissues (Fig. 8e). Representative images of tissues stained by antibodies to integrins αX/β2, hnRNPLL, and ABCG1 are shown in Fig. 8d–i. Staining failed for six tissues. Aside from those tissues, out of the 144 cancer tissue samples stained with the E01 antibody, 105 (72.9%) exhibited positive expression of ECM1a, and 72 of the total 144 samples (50%) were categorized as having high expression of ECM1a. Of the 39 ECM1a-negative cancer tissues, 33 tissues exhibited strong staining with the E02 antibody, suggesting that these 33 tissues expressed only ECM1b. Of the 105 ECM1a-positive tissues, 72 tissues also showed positive staining with the E02 antibody, suggesting that either ECM1a or both ECM1a and ECM1b were expressed in these tissues; however, we regarded these cases as having low ECM1b expression (Table 1). The correlations between ECM1a or ECM1b expression and integrin αX, integrin β2, hnRNPLL, or ABCG1 expression are presented in Supplementary Tables 3 and 4. Significant positive correlations were found between ECM1a expression and integrin αX, integrin β2, hnRNPLL, or ABCG1 expression, but significant negative correlations were detected between ECM1b expression and the expression of other molecules (Supplementary Table 5), indicating that ECM1a and ECM1b play opposing roles during ovarian tumorigenesis.

**Table 1:** Association of ECM1a, ECM1b, integrin αX, integrin β2, ABCG1, and hnRNPLL expression (low/high) with patient characteristics.

	Items	ECM1a			ECM1b			Integrin αX			Integrin β2			hnRNPLL			ABCG1		
		Low	High	^a^*p*	Low	High	*p*	Low	High	*p*	Low	High	*p*	Low	High	*p*	Low	High	*p*
Age at diagnosis (year)	≤57	46	34		60	20		27	53		41	39		56	24		33	47	
	>57	26	38		51	13		23	41		36	28		42	22		15	49	
	Total	72	72	**0.018**	111	33	0.128	50	94	0.134	77	67	0.112	98	46	0.123	48	96	**0.011**
Ascites (ml)	≤1500	44	40		61	23		29	55		46	38		54	30		25	59	
	>1500	28	32		50	10		21	39		31	29		44	16		23	37	
	Total	72	72	0.107	111	33	0.053	50	94	0.14	77	67	0.126	98	46	0.077	48	96	0.08
Intestinal metastasis	No	37	24		50	11		29	32		38	23		47	14		22	39	
	Yes	35	48		61	22		21	62		39	44		51	32		26	57	
	Total	72	72	**0.012**	111	33	0.08	50	94	**0.003**	77	67	**0.026**	98	46	**0.02**	48	96	0.119
Mediastinal metastasis	No	26	27		40	13		24	29		33	20		38	15		23	30	
	Yes	46	45		71	20		26	65		44	47		60	31		25	66	
	Total	72	72	0.135	111	33	0.152	50	94	**0.019**	77	67	**0.038**	98	46	0.115	48	96	**0.022**

^a^Two-sided Fisher exact test is used for statistical analysis.

Bold values indicate statistical significance.

To link the expression of the above molecules with the major clinicopathological characteristics of patients with ovarian cancer, we analyzed the associations between the expression of these markers and age, ascites, and intestinal or mediastinal metastasis in ovarian cancer patients. Patient age (<57 or >57 years) was significantly associated with the protein expression of ECM1a (*p* = 0.018) or ABCG1 (*p* = 0.011). Intestinal metastasis (No or Yes) was significantly associated with ECM1a (*p* = 0.012), integrin αX (*p* = 0.003), integrin β2 (*p* = 0.026), or hnRNPLL (*p* = 0.02) expression. Mediastinal metastasis (No or Yes) was significantly associated with integrin αX (*p* = 0.019), integrin β2 (*p* = 0.038), or ABCG1 (*p* = *0.022*) expression, whereas no significant association was found between ascites (≤1500 ml or >1500 ml) and any of the detected markers (Table 1).

Finally, we analyzed the association of ovarian cancer patient survival with these markers. We found that poor overall survival (OS) and disease-free survival (DFS) were significantly associated with ECM1a (*p* < 0.001, *p* = 0.003), integrin αX (*p* = 0.003, *p* = 0.006), integrin β2 (*p* = 0.011, *p* = 0.02), hnRNPLL (*p* = 0.007, *p* = 0.023), and ABCG1 (*p* = 0.006, *p* < 0.001) expression, whereas good OS and DFS were significantly associated with ECM1b expression (*p* < 0.001, *p* = 0.003) (Fig. 8j). These results suggest that these markers can be used as prognostic markers for patients with ovarian cancer.

## Discussion

Although the gene for ECM1 can be transcribed into four mature mRNAs via alternative pre-mRNA splicing, the individual functions of the differently encoded subtypes have rarely been reported. In addition, the putative ECM1 receptor has not yet been identified. Studies have suggested that some ECM molecules interact with integrins. For example, laminin can bind to integrins α1β1 and α2β1, fibronectin binds to both integrin αVβ3 and integrin αVβ6, and fibrillin binds to integrin αVβ3^37^. However, the binding of ECM1 to integrins has not been reported to date. In our study, we found that ovarian tumorigenesis was controlled mainly by the ECM1 subtype ECM1a, which bound to integrin αXβ2 through the GPR motif to promote phosphorylation of AKT/FAK/Paxillin/Rac. Mutation of the GPR motif in ECM1a to VAQ eliminated the ability of ECM1a to bind to integrin αXβ2, activate AKT/FAK/Paxillin/Rac signaling and induce tumor growth. In addition, we found that ECM1a-WT bound to integrin β1, JUP, Annexin A1, Calnexin, and S100A9 in addition to integrin αXβ2 and that ECM1a-MT could still bind to other cell surface molecules, such as Plakophilin-1 and S100A8. These findings indicate that the binding between ECM1a and integrin αXβ2 does not prevent other cell surface molecules from accessing all of the binding sites but does competitively inhibit the binding between ECM1a and some other cell surface members, such as Plakophilin-1 and S100A8. On the other hand, our data also suggest that the binding of ECM1 to αXβ2 may differentially affect the abilities of other ligands, such as FGA, FGG, ICAM1, PLG, iC3b, CD90, and heparin/PF4, to bind αXβ2 and thus may regulate the function of these molecules.

ECM1b was not secreted from cells and did not bind to integrin αXβ2, which prevented activation of AKT/FAK//Rac/Myosin-mediated signaling and induction of ovarian cancer cell tumor growth. This finding is partially consistent with an early report that ECM1 might function as a tumor suppressor in hepatic cancer through epigenetic modification^8^. A recent study has reported that ECM1b localized at the ER may act as a tumor suppressor in ESCC through inhibition of MTORC2/MYC/MTORC1 signaling^9^; however, the mechanism of ECM1b in cancer cells has not yet been systematically characterized. In the current study, we found that nonsecretory ECM1b directly bound to nonphosphorylated myosin to potentially inhibit its phosphorylation, whereas ECM1a did not bind to myosin but promoted myosin phosphorylation through activation of AKT/FAK/Paxillin/Rac signaling. Therefore, phosphorylation of myosin may contribute to signaling downstream of the ECM1-integrin αXβ2 interaction, which is to a certain extent consistent with a report showing that ECM1 regulates the actin cytoskeletal architecture of breast cancer cells^7^. Moreover, the inhibition of myosin phosphorylation caused by direct binding of ECM1b to myosin may provide some important insights for the design of unique therapeutic compounds or small peptides that can improve cancer treatment efficacy once the binding mechanism between ECM1b and myosin is unraveled.

hnRNPLL, a master regulator of activation-induced alternative splicing in T cells, was first found to specifically alter the splicing of *CD45*, which encodes a tyrosine phosphatase that is essential for T cell development and activation^38^. Acting as one of the mRNA splicing repressors, hnRNPLL not only controls a wide range of mRNA splicing in memory T cells^39^ and pre-mRNA splicing in plasma cells along with the transcription elongation factor ELL2^40^ but also mediates a genome-wide RNA processing switch during the differentiation of primary B cells to plasma cells^41^. A recent study has shown that hnRNPLL functions as a suppressor of metastasis to inhibit the splicing level of variable exon 6 in *CD44* (CD44v6), a marker of poor prognosis for colorectal cancer^42^. However, whether hnRNPLL regulates the splicing of *ECM1* is unknown. In the current study, we found that ECM1a expression promotes the expression of *hnRNPLL*, while hnRNPLL in turn enhances ECM1a production through preferential mRNA splicing of *ECM1* over *ECM1b* or *ECM1c*. Although other mRNA splicing repressors, including hnRNPL, hnRNPM, and hnRNPU, were upregulated by ECM1a, silencing of their expression failed to alter ECM1 mRNA splicing. However, analysis of SRSFs that function as splicing activators and are counterfactors of hnRNPs showed that SRSF1 and SRSF6 were upregulated by ECM1a and ECM1b, respectively. Furthermore, overexpression of SRSF1 increased the mRNA splicing of ECM1b, whereas silencing of SRSF6 expression inhibited ECM1a mRNA splicing, and hnRNPLL did not affect the mRNA stability of ECM1 isoforms during cell proliferation. Therefore, selectively targeting hnRNPLL, SRSF1, and SRSF6 may usher in an important new era of ovarian cancer treatment.

ABCG1 (also known as ABC8), a member of the superfamily of ABC transporters, mainly regulates cholesterol homeostasis^43–46^ and is therefore associated with arteriosclerosis^47^. However, a few studies have indicated that ABCG1 is also connected with human cancer^48,49^. In our study, we proved that ABCG1 expression is upregulated by ECM1a but not by ECM1b. Overexpression of ABCG1 in ECM1-silenced cells activates AKT/FAK/paxillin/Rac/Myosin signaling to promote tumorigenesis. ABCG1 also interacts with phosphorylated AKT, paxillin, and myosin. Thus, we have uncovered a function of ABCG1 in association with ECM1-mediated tumorigenesis. One might wonder how ABCG1 facilitates the phosphorylation of downstream signaling molecules, including AKT/FAK/Paxillin/Rac/Myosin. Although ABCG1 transports ATP, no available evidence supports the idea that ABCG1 is a kinase. In an in vitro kinase assay, we found that eukaryotic ABCG1 could directly phosphorylate AKT2, which may further trigger ECM1a-integrin αXβ2 interactive signaling. On the other hand, we also found that the serine/threonine phosphatase subunit PPP2CB and the serine/threonine phosphatase inhibitor subunit PPP1R14C might participate in the serine phosphorylation regulation of AKT, Rac, and Myosin. These data suggest that the ECM1-induced activation of AKT/Rac/Myosin signaling is regulated by multiple signaling molecules, including ABCG1 and/or protein phosphatases and their inhibitors. However, the regulation of tyrosine-phosphorylated FAK (Y397) and Paxillin (Y118) needs additional investigation.

As ABC transporters are responsible for transport of small particles, including nutrients, peptides, cholesterol, and small compounds such as drugs, cancer cell chemoresistance has been reported to be associated with ABC transporters in numerous studies. One study has found that ABCB1 and ABCG2 might mediate chemoresistance in HER2-positive small cell lung cancer cells^50^. In addition, inhibition of ABCC4 attenuates the proliferation of neuroblastoma cells and sensitizes tumors to irinotecan treatment^51^. Platinum-based chemoresistance is the largest barrier to successful chemotherapy in many cancers, including ovarian cancer, but we show that ABCG1 significantly induces cisplatin resistance in ECM1-associated cancer cells via induction of CD326-mediated stemness, which is an original finding of this study. Other studies have shown that some ABC transporters, including ABCA2, ABCA3, ABCB1, and ABCG2, are associated with cancer cell stemness^52–54^. Our study reveals that ABCG1 upregulates the expression of stem cell transcription factors, including c-Myc, Oct4A, and SOX2, to enrich the CD326+ cell population and that the CD326+ cell population can be further enriched by treatment of CD326+ cells with a low dose of cisplatin. Therefore, targeting ABCG1 may enhance chemotherapeutic efficacy for cancers, especially those with ECM1a/ABCG1 activation.

We also found that ECM1-associated signaling may regulate intracellular cholesterol homeostasis through ABCG1-mediated cholesterol efflux and total cholesterol control. Although one study has found that knockout of *ABCG1* in mice inhibits tumor growth by modulating macrophage function in terms of cholesterol homeostasis within the tumors^55^, the reports that serum cholesterol levels are associated with ovarian cancer are quite controversial^56–58^. Therefore, whether the ECM1a/ABCG1-mediated regulation of cholesterol levels in plasma or the tumor microenvironment can directly indicate a risk for or diagnosis of ovarian cancer requires more detailed investigation. Nevertheless, the results of our clinical analyses showed that ECM1a, integrin αXβ2, hnRNPLL, and ABCG1 were positively correlated with each other and that these molecules may be independent prognostic factors for poor survival in patients with ovarian cancer. ECM1b was associated with improved patient survival, while ECM1c was undetectable. Therefore, ECM1 subtypes, integrin αXβ2, hnRNPLL, and ABCG1 can be used as diagnostic or prognostic markers.

All of our data suggest that ECM1a plays a tumorigenic role and confers cancer cell cisplatin resistance through integrin αXβ2/hnRNPLL/ABCG1-mediated cellular phosphorylation signaling and stemness induction. Whether our findings of the opposite tumorigenic functions between ECM1a and ECM1b, cytoskeletal phosphorylation induced by the ECM1a-integrin αXβ2 interaction, hnRNPLL-regulated ECM1a mRNA splicing, and ABCG1-mediated cytoskeletal phosphorylation and cancer stemness apply to different cancer cell types warrants comprehensive investigation. Nevertheless, the involved molecules may be selectively targeted for cancer therapy.

## Methods

### Cell lines and cell culture

The human ovarian epithelial cancer cell line SKOV3 was purchased from the American Tissue Culture Collection (ATCC), and the human ovarian epithelial cancer cell lines Hey, HeyA8, SKOV3ip1, OVCA429, OVCA433, ES-2, and A2780 were obtained from Dr. Bast, R.C. Jr of the University of Texas MD Anderson Cancer Center, Houston, Texas (USA). The immortalized HOSE cell line T29 has been described previously^59^, and the normal HOSE cell line and the immortalized FTE cell line were established in our laboratory^60,61^. All cell lines were cultured in RPMI 1640 medium containing 10% fetal bovine serum, 2 mM L-glutamine, penicillin (100 units/ml), and streptomycin (100 μg/ml) at 5% CO_2_ and 37 °C.

Each cell line was routinely tested and found to be free of mycoplasma contamination before each experiment was conducted. The SKOV3, Hey, OVCA429, OVCA433, and A2780 cell lines were authenticated by a third party (the ATCC or Genetic Testing Biotechnology Co., Ltd., in Suzhou, China) according to the STR data in the public database. The HeyA8 and SKOV3ip1 cell lines were derived from the Hey and SKOV3 cell lines, respectively, so they are isogenic cell lines^62^. However, HeyA8 and SKOV3ip1 cells do not have STR data in public databases, so we had these two cell lines authenticated by comparison of their STR data with those of the Hey and SKOV3 cell lines. The authentication documents are available upon request.

### Construction of plasmids for gene silencing, overexpression, and mutation

To silence gene expression, synthesized DNA oligos for the transcription of specific shRNAs designed to target *ECM1*-specific mRNAs (primers 1–6; Supplementary Table 6), integrin *αX* mRNA (primers 13–18; Supplementary Table 6), integrin *β2* mRNA (primers 21–26; Supplementary Table 6), *hnRNPLL* mRNA (primers 31–36; Supplementary Table 6), or *ABCG1* mRNA (primers 41–46; Supplementary Table 6) were inserted separately into plko.1/puromycin, zeocin, blasticidin, and neomycin vectors. In addition, a scrambled shRNA was used as the negative control^62^. In a pilot study, we first designed three shRNAs at the 5′ end of *ECM1* mRNA to target all *ECM1* subtypes, including *ECM1a*, *ECM1b*, *ECM1c*, and *ECM1d*, but the silencing efficiency was very low. Thus, we next designed shRNAs to target only the intermediate sequences of *ECM1* mRNA included in *ECM1a*, *ECM1b*, and *ECM1c*. The results of later experiments suggested that ECM1d is largely dispensable for tumorigenic function in ovarian cancer cells; thus, our experimental design excluded ECM1d.

cDNAs for *ECM1* isoforms 1a, 1b, and 1c were amplified from HeyA8 cells by RT-PCR using primers 7 and 8 (Supplementary Table 6) and inserted into the PCDH-puromycin, PCDH-zeocin/hygromycin, and PCDH-GFP vectors, respectively. To generate *ECM1a-MT* with VAQ instead of GPR, mutagenic primers 9 and 10 (Supplementary Table 6) were employed to perform PCR by using a QuikChange site-directed mutagenesis kit according to the manufacturer’s protocol (200523, Stratagene, La Jolla, CA, USA), and *ECM1a-MT* cDNA was cloned into the PCDH-zeocin vector. The cDNAs for *hnRNPLL*, *ABCG1, and SRSF1* were cloned similarly using *hnRNPLL-, ABCG1-, and SRSF1-*specific primers 29–30, 39–40, and 73–74 (Supplementary Table 6), respectively, and inserted into the PCDH-puromycin or PCDH-neomycin/hygromycin vector. Correct plasmids were screened with DNA sequencing by Jinweizhi Co., Ltd. (Suzhou, China). Empty vectors were used as negative controls.

### Establishment of Supplementary table cell lines by gene silencing or overexpression

Silencing of *ECM1* expression with shRNA in HeyA8 cells through lentivirus-mediated delivery generated A8i cells (the control cells were designated A8-ctrli); these cells were selected with puromycin at 2 µg/ml for 5 days. Overexpression of ECM1a, ECM1b, and ECM1c was performed in A8i cells to generate A8i-ECM1a (A8i-A), A8i-ECM1b (A8i-B), and A8i-ECM1c (A8i-C) cells, which were selected either by flow cytometry (GFP±) or by growth in the presence of zeocin or hygromycin at a concentration of 100 µg/ml or 1 µg/ml for 2 weeks. Transfection of A8 cells with ECM1b was similarly conducted to confirm the function of ECM1b in the presence of ECM1a and ECM1c. Delivery of ECM1a or ECM1b cDNA into ECM1b- or ECM1a-overexpressing cells was also carried out to investigate the overlapping functions between ECM1a and ECM1b.

A8i-A-αXi, A8i-A-β2i, or A8i-A-β2i-αXi cells were generated by silencing of integrin *αX* and/or *β2* expression in A8i-A cells via lentiviral vector-based infection and selection with zeocin at 100 µg/ml for 2 weeks, blasticidin at 50 µg/ml for 4 weeks, or zeocin at 100 µg/ml for 2 weeks. A8i cells expressing ECM1a-MT (zeocin) were established via lentiviral vector-based infection and selected with zeocin at 100 µg/ml for 2 weeks.

A8i cells (with puromycin resistance) or A8i-A cells (with puromycin and zeocin resistance) with overexpression or silencing of *hnRNPLL* were established similarly and selected with neomycin or hygromycin for 1 week at 100 or 1 µg/ml, respectively. SKOV3 cells overexpressing ECM1a or ECM1b were selected with puromycin at 1 µg/ml for 1 week, whereas OVCA429, OVCA433, and ES-2 cells overexpressing ECM1a were selected with puromycin at 2 µg/ml for 5 days. T29 and FTE cells expressing ECM1a or ECM1b were selected with puromycin at 0.25 µg/ml for 2 weeks.

All control cell lines were generated by infection with viruses containing the empty vector or a scrambled shRNA vector following the same protocol.

### RNA-seq and gene expression analysis

Total RNA was isolated from each cell sample using the standard TRIzol protocol (Invitrogen, Carlsbad, CA, USA). RNA quality was examined by gel electrophoresis and with a NanoDrop spectrophotometer (Thermo, Waltham, MA, USA). High-throughput mRNA sequencing (RNA-seq) was carried out using an Illumina HiSeq 2500SBS instrument and cBot cluster generation (Genergy Biotech, Co., Ltd., Shanghai, China). Qualified reads were mapped to the human hg19 reference genome (hg19) at the same time with TopHat (v2.1.0) with the default parameters. Next, Cufflinks (v2.2.1) was used to estimate the gene expression levels in the alignment file, and the gene abundances were reported as fragments per kilobase of transcript per million fragments mapped (FPKM) values. Because the low-quality bases were concentrated at the ends of the Illumina sequencing reads, Trim Galore software was used to dynamically remove adapter sequence fragments and low-quality fragments at the 3′ ends, and FastQC software was used to conduct quality control of the preprocessed data. A total of 10–12 Gbp of data was obtained with the PE150 sequencing model. We counted the detected genes (FPKM > 0) that mapped to the human hg19 reference genome in each sample separately and constructed a scatter plot. Differentially expressed genes (DEGs) between different time points (12 hpt vs. 0 hpt, 36 hpt vs. 0 hpt, and 72 hpt vs. 0 hpt) were determined using the MA-plot-based method with random sampling model in the DEGseq package^63,64^. The thresholds for DEGs were a *p* < 0.05 and an absolute fold change ≥2. Then, DEGs were chosen for functional and signaling pathway enrichment analysis using the GO and KEGG databases. Pathways with *p* values < 0.05 were considered significantly enriched, and those that were associated with at least two affiliated genes were retained. Heatmaps were generated by using gene clustering data based on analysis of the RNA-seq data.

### Liquid chromatography (LC)-MS/MS analysis

To investigate the potential proteins binding to ECM1, a total of 500 μg of CL extracted from A8i-V, A8i-A, and A8i-B cells with radioimmunoprecipitation (RIPA) buffer containing a proteinase inhibitor was mixed with 2 μg of HA antibody (#2367, CST) and 50 μl of protein G-conjugated agarose beads, and the mixture was incubated with rocking at 4 °C overnight. The mixture was then centrifuged at 15,000 × *g* at 4 °C for 1 min, and the beads were washed with prechilled TBS (pH 7.4) containing 0.1% Tween-20 and centrifuged five times under the same conditions. The beads were finally centrifuged for 2 min, and the supernatant was completely removed. Then, 50–100 μl of 2× SDS loading buffer was added to the washed beads, and the mixture was boiled at 100 °C for 15 min. The samples were then separated by 10% SDS-PAGE and stained with Coomassie Brilliant Blue R-250. The specific bands for proteins that were differentially expressed between A8i-A and A8i-B cell samples after destaining with 15% methanol and 3% acetic acid were cut out and washed with ddH_2_O until colorless. The proteins were extracted from the gel, resuspended in 100 μl of ddH_2_O, and analyzed by MS via high-performance liquid chromatography and a Q Exactive Mass Spectrometer (Thermo Scientific, Waltham, Massachusetts) at Shanghai Applied Protein Technology Co., Ltd. The original files were transformed with Proteomics Tools 3.1.6 software, and Mascot 2.2 software was used for database screening. For protein identification, the following options were used. Peptide mass tolerance = 20 ppm, MS/MS tolerance = 0.1 Da, Enzyme = Trypsin, Missed cleavage = 2, Fixed modification: Carbamidomethyl (C), Variable modification: Oxidation (M).

To analyze the differences between membrane proteins binding to ECM1a-WT and ECM1-MT, total CLs, membrane fractions obtained from A8i-ECM1a-WT (A8i-A) and A8i-A-MT cells, or membrane fractions obtained from A8 cells pretreated with the CM of A8i-A-WT and A8i-A-MT cells were used in the same way to perform the co-IP assays and MS analysis described above.

### Cell treatments and generation of ECM1 subtype-specific antibodies

Cells at 75% confluence were treated with okadaic acid (20 nM, O7885, Sigma-Aldrich) for 24 h; ceramide (20 µM, 22244, Sigma-Aldrich) for 24 h; antibodies against HA, ECM1, integrins αX and β2 (1 μg/ml for 24 h); or bioactive ECM1 (#3937-EC050, R&D Systems Inc.) at 0.05 µg/ml for 24 h. The above cellular molecules were then detected. To enable specific identification of the subtypes of ECM1, polyclonal rabbit antibodies against ECM1a/ECM1c (Antigen A01), ECM1a/ECM1b/ECM1c (Antigen A02), or ECM1c (Antigen A03) were produced by Wuhan Pujian Biotechnology Co., Ltd. (Wuhan, Hubei, China).

### Semiquantitative RT-PCR and qRT-PCR

To determine the subtypes of *ECM1* mRNA in cell lines, we isolated RNA with TRIzol (Invitrogen, Waltham, MA) and performed semiquantitative RT-PCR with primers 51 and 52; *GAPDH* was used as the internal control and was amplified by primers 85 and 86 (Supplementary Table 6). The PCR products were analyzed in 1.8% agarose gels containing 0.5% ethidium bromide and visualized and photographed under ultraviolet light. All PCR products were purified using a Gel Extraction Kit (Qiagen, Germantown, MD, USA) and sequenced.

To perform qRT-PCR, total RNA from cells was isolated by using TRIzol reagent (Invitrogen, Waltham, MA). RNA RT was carried out by using a PrimeScript™ RT Master Mix kit (RR036A, Takara Bio Inc., Takara, Japan) according to the manufacturer’s instructions. The cDNA was subsequently analyzed via qPCR on a LightCycler®/LightCycler® 480 System (Corbett Research, Sydney, Australia) by using a SYBR® Premix Ex Taq^TM^ Kit (RR420A, Takara Bio Inc., Japan) and qPCR primers 11–12 for *ECM1*, 19–20 for integrin *αX*, 27–28 for integrin *β2*, 37–38 for *HNRNPLL*, 47–48 for *ABCG1*, 49–50 for *ACTB*, 57–58 for *hnRNPL*, 59–60 for *hnRNPM*, 61–62 for *hnRNPU*, 63–64 for *SRSF1*, 65–66 for *SRSF6*, 81–82 for *PPP2CB*, and 83–84 for *PPP1R14C* (Supplementary Table 6). The absence of primer-dimer formation for each oligonucleotide set was validated by establishing the melting curve profile. The gene expression levels were calculated by the ΔΔ*C*_t_ method.

### Co-IP, MS analysis, and WB analysis

To perform co-IP, 0.5–1.0 mg of whole-CL or cell membrane fractions from relevant cells pretreated or not pretreated with CM was prepared with RIPA lysis buffer (10 mM Tris-Cl [pH 8.0], 1 mM EDTA, 0.5 mM EGTA, 1% Triton X-100, 0.1% sodium deoxycholate, 0.1% SDS, and 140 mM NaCl) or extracted with a kit (#P0003) from Beyotime Biotechnology (Shanghai, China), respectively. Two micrograms of antibodies against specific proteins or normal IgG from the same species were used for the assay. The co-IP products were separated by SDS-PAGE, and the specific protein bands were analyzed by MS after Coomassie Brilliant Blue R-250 staining. The primary antibodies used for co-IP targeted ECM1, HA, integrin β2, integrin αX, pPaxillin (Y118), pMyosin IIa (S1943), ABCG1, integrin β1, JUP, Annexin A2, Calnexin, S100A9, Plakophilin-1, S100A8, FGA, FGG, ICAM1, PLG, iC3b, CD90/Thy-1, and heparin/PF4. Detailed information on the antibodies is listed in Supplementary Table 7.

WB analysis was used for routine detection of protein expression in different samples, which were prepared as CLs or as cell culture medium concentrated with Amicon Ultra-4 Centrifugal Filter Devices (Amicon Ultra 50 K device, 50,000 MW CO, Millipore, Massachusetts, USA) after centrifugation at 7500 × *g* for 40 min. The condensed samples (~200 µl) were mixed with 5× loading buffer, boiled for 3 min and then analyzed by WB analysis.

For routine WB, primary antibodies were purchased from different companies to detect ECM1, integrin αX, integrin β2, AKT, pAKT (S473), FAK, pFAK397, Paxillin, pPaxillin, Myosin IIa, pMyosin IIa S1943, pRac (S71), hnRNPLL, ABCG1, integrin β1, JUP, Annexin A1, Calnexin, S100A9, Plakophilin-1, S100A8, FGA, FGG, ICAM1, PLG, iC3b, CD90/Thy-1, heparin/PF4, hnRNPL, hnRNPM, hnRNPU, SRSF1, SRSF6, PPP2CB, PPP1R14C, HA, Flag, and myc. Antibodies against β-actin and GAPDH were purchased from CST. Detailed information on the antibodies is listed in Supplementary Table 7.

### IF staining

Double staining for integrin αX or integrin β2 and ECM1 was performed, and colocalization was assessed as before^65,66^; nuclei were stained with 4′,6-diamidino-2-phenylindole (DAPI). CM, which presumably contained ECM1a (with HA), ECM1b (with HA), or ECM1a-MT, was incubated with HeyA8 cells to detect the binding of ECM1 to integrins αX and β2 by using an established protocol^66^; antibodies against ECM1, integrin αX, integrin β2, and HA were used. Detailed information on the antibodies is listed in Supplementary Table 7.

Cells that cultured overnight on cover slides were used for IF staining^67^. Primary antibodies were used to detect Myosin IIa, pMyosin IIa (S1943), and ABCG1; TBS (pH 7.2) was used instead of phosphate-buffered saline (PBS) to detect phosphorylated proteins. The following secondary antibodies were purchased from Molecular Probes (Invitrogen, Carlsbad, CA): donkey anti-rabbit IgG 488 (green, 1:2000, A21206), donkey anti-mouse 555 (red, 1:2000, A31570), donkey anti-rabbit 555 (red, 1:2000, A31572), donkey anti-mouse 488 (green, 1:2000, A21202), and donkey anti-goat 488 (green, 1:2000, A11055). Detailed information on the antibodies is listed in Supplementary Table 7. Nuclei were also stained with DAPI (Sigma-Aldrich. St. Louis, MO, USA). The results were detected and photographed with a confocal microscope equipped with a Nikon N1CCD camera, and the images were processed with NIS Viewer software.

### 3D cell culture and spheroid formation

Rat tail collagen (#354236, Corning, Corning, NY, USA) was diluted to 50 µg/ml with 0.02 N acetic acid; 500 µl was added to a 24-well plate and incubated at room temperature for 1 h. Then, the remaining solution was carefully aspirated, and PBS was used to rinse the wells 2–3 times to remove acid. Cells were added to an appropriate volume of RPMI 1640 medium to generate a cell concentration of 250 cells/500 µl in one tube. Another tube contained RPMI 1640 medium, collagen and NaOH at a ratio of 5:1:0.0235. The two tubes were mixed well on ice for plating. After 1 week of incubation, spheroids were counted under a phase-contrast microscope. The results are presented as a percentage representing the number of spheroids divided by the initial number of cells seeded.

### Transient transfection of siRNAs

Cells were transiently transfected with siRNAs 53, 54, and 55 specifically targeting *hnRNPL*, *hnRNPM*, and *hnRNPU*, respectively; with three pair of siRNAs (67–72) targeting *SRSF6*; or with control siRNA (56) (Supplementary Table 6) according to the protocol supplied by the manufacturer. Lipofectamine® RNAiMAX (Invitrogen, USA) was used for siRNA transfection. Cells were seeded in 6-well culture plates overnight (1 × 10^6^ cells/well for WB analysis), and the culture medium was then changed to Opti-MEM. The transfection system was prepared with 9 µl of Lipofectamine® RNAiMAX diluted in 150 µl Opti-MEM and then mixed with siRNA-containing Opti-MEM. After 15 min of mixing, the transfection mixture was added to the wells for 48 h. The resulting cells were used for WB analysis or other analyses.

### Protein–protein PLA and cell surface biotinylation labeling

To identify whether ECM1a (wild-type [WT] or MT) truly binds to integrins αX and β2, cells cultured on cover slides at 75% confluence were subjected to PLA by using a Duolink® In Situ Red Starter Kit Mouse/Rabbit (#DUO92101, Sigma-Aldrich) and a Duolink® PLA Control Kit—PPI (#DUO92202, Sigma-Aldrich). The experimental protocol included blocking, primary antibody incubation, PLA probe incubation, ligation, amplification, washing, and photography after mounting with Duolink® In Situ Mounting Medium containing DAPI. Proper control assays were performed with mouse IgG or reagents in the control kit, and the cytoskeleton was stained with phalloidin. The assays were repeated at least three times.

To confirm that ECM1a was secreted and bound to cell surface proteins, including integrin αXβ2, cell surface biotinylation was performed with a Pierce Cell Surface Protein Isolation Kit (Thermo Scientific™, #89881) containing EZ-Link Sulfo-NHS-SS-Biotin and NeutrAvidin Agarose according to the supplier’s instructions. The isolated cell surface components were first detected with antibodies against ECM1a (HA), integrin αX, and integrin β2. A co-IP assay was also performed with an antibody against HA, and the co-IP product was detected with either an antibody against integrin αX or an antibody against integrin β2 (Supplementary Table 7). Control samples were generated with mouse or rabbit IgG. The assays were repeated more than three times.

### Expression and purification of ABCG1 and AKT2

To obtain the GST fusion proteins of ABCG1 and AKT2 in eukaryotic or prokaryotic cells, human *ABCG1* or *AKT2-WT/MT* (S474A) cDNAs were first constructed using the primers 75–76 or 77–80 (Supplementary Table 6) either into pEBG with *Bam*HI/*Not*I or into pGEX-4T-3 with *Bam*HI/*Eco*RI, respectively. Then, pEBG/*ABCG1* was transfected into HEK293T cells for 48 h. pGEX-4T-3/*AKT2-WT* and pGEX-4T-3/*AKT2-MT* were transfected into *E. coli* BL21(DE3) cells that were later induced with 0.4 mmol/l isopropyl β-D-1- thiogalactopyranoside for 8 h at 28 °C. Afterwards, ABCG1 or AKT2-WT/MT GST fusion proteins (~1 mg) were incubated with 40 µl of prepared glutathione Sepharose beads at 4 °C for 2 h with gentle rotation. After removing the supernatant, the beads were collected and washed three times with PBST. The target proteins were eluted and resolved with elution buffer (20 mM Tris [pH 8.0], 10 mM reduced glutathione) and then confirmed by WB analysis. MK-2206 (Selleckchem, 600 µM), an AKT-specific phosphorylation inhibitor, was added before the cells were lysed during the purification of AKT2. AKT2 phosphorylation was expected to be much lower in the presence of MK-2206 than in the absence of MK-2206 as detected by WB analysis.

### In vitro kinase assay and cholesterol measurement

An in vitro kinase assay was performed at 25 °C for 90 min in kinase buffer containing 50 µM ATP and 50 µM DTT according to the protocol of the ADP-Glo^TM^ Kinase Assay Kit (Promega, V9101). The reactions were optimized via selective addition of eukaryotic lysates, AKT2-WT, AKT2-MT, ABCG1, and MK-2206 (Selleckchem, 600 µM). AKT2 was used as a substrate, whereas ABCG1 or eukaryotic CLs were used as kinases. Purified AKT2-WT without MK-2206 pretreatment during purification was used as a phosphorylated positive control. The kinase reaction was terminated in half of each sample via addition of ADP-Glo^TM^ Reagent, and the remaining ATP was then depleted. Kinase Detection Reagent was then added to the samples to convert ADP to ATP and to enable the newly synthesized ATP to be measured using a luciferase reaction. The kinase reaction was terminated in the other half of each sample via addition of SDS loading buffer. The protein was boiled for 3 min and loaded for protein separation on a 10% SDS-PAGE gel. The proteins were transferred onto PVDF membranes and detected by chemiluminescence using the standard methods^67^.

Cholesterol efflux and total cholesterol in cells expressing ECM1 subtypes, hnRNPLL, and ABCG1 were measured using the Cholesterol Efflux Assay Kit (MAK192, Merck/Sigma-Aldrich) and Cholesterol Quantitation Kit (MAK043, Merck/Sigma-Aldrich) according to the protocols supplied by the manufacturer.

### Detection of cisplatin resistance and stemness

To detect the cytotoxicity induced by cisplatin, 3000 cells were seeded in 96-well plates. The cells were treated for 48 h with a fivefold gradient of cisplatin (concentrations ranging from 0.64 to 2000 µM). After 24 h of recovery after removal of cisplatin, 10 µl of CCK-8 reagent (Biyuntian, China) was added per well. The absorbance value at a wavelength of 490 nm was detected with a microplate reader (BioTek, USA) after 4 h of treatment. The inhibition rate was calculated using the following formula: inhibition rate = (OD^blank^ − OD^cisplatin^)/(OD^blank^ − OD^DMSO^). The IC50 values were calculated using GraphPad Prism. The expression of stem cell transcription factors was detected by WB analysis using the antibodies against c-Myc, SOX, and Oct4A listed in Supplementary Table 7.

To examine cancer cell stemness, a single-cell suspension with ~1 × 10^6^ cells was prepared for staining with antibodies against CD117, CD24, CD326, and CD133 or a control antibody (IgG κ isotype control) (Supplementary Table 7). The cells were stained at 4 °C for 30 min in the dark, washed with PBS, resuspended in 100 µl of PBS after centrifugation, and finally subjected to flow cytometry analysis. The flow cytometers used for analysis and selection were Beckman Cytomics FC 500 MPL and MOFLO XDP (Beckman Coulter, USA), respectively. The cells were first gated on a forward scatter/side scatter plot (a) and then gated on specific populations. The gating strategy is provided in Supplementary Fig. 15 of the Supplementary Information. Cell population abundance was determined by comparing the stained cells with negative controls. Specific dye-conjugated antibodies used to stain the cell population was gated by specified laser channels recognizing PE and FITC.

### Mouse tumor growth assays

To test the in vivo functions of various genes, we performed mouse tumor growth assays. The number of mice (sample size) required to reach statistical significance was determined in preliminary pilot studies that used the following formula^62^: *n* = 16 × (standard deviation/difference in mean tumor volume)^2^ + 1. The results of the pilot study indicated that at least five mice were required to detect differences in tumor size with 80% power at a *p* value of <0.05. Statistical analysis was carried out using *t*-Test at different time points for the mean tumor sizes of each group.

To assess subcutaneous tumor growth, 4- to 6-week-old BALB/c athymic nude female mice were maintained in a pathogen-free environment. Briefly, cells were harvested by trypsinization, washed twice with PBS, and then resuspended in PBS. At least five mice were used per cell line, and each mouse received one subcutaneous injection of 1 × 10^6^ cells for SKOV3-series cell lines; 3 × 10^6^ cells for HeyA8-, OVCA429- and OVCA433-series cell lines; and 5 × 10^6^ cells for T29- and FTE-series cell lines. A volume of 150 µl of PBS was used for each injection. Tumor growth was monitored every 3 days, and the tumor volume was calculated with the following formula: tumor volume (in mm^3^) = a × b^2^ × 0.52^66^. When the largest tumor reached 1.5–2.0 cm in diameter, all mice in the same group were sacrificed simultaneously by CO_2_ inhalation. For peritoneal tumor growth assessment, 1 × 10^7^ cells for the HeyA8-series cell lines were injected into each mouse, and a total of five female mice were injected for each cell line. The animals were observed for poor appetite, lethargy, abdominal enlargement, severe weight loss, etc. Tumor tissues were isolated and weighted. The mouse experiments were approved by the Institutional Animal Care and Use Committee of Fudan University and were performed following institutional guidelines and protocols. The assays were repeated three times.

### TMA analysis and IHC

To identify the different subtypes of ECM1, we ordered three custom rabbit polyclonal antibodies from Wuhan Pujian Biotechnology Co., Ltd. (Wuhan, Hubei, China) to specifically detect the expression of ECM1a, ECM1b, and ECM1c. An ECM1-01 antibody (Ab-01) was generated from peptide LRRFRSVPRNLPAT to recognize aa 315–328 of ECM1a and aa 342–355 of ECM1c. An ECM1-02 antibody (Ab-02) was generated from peptide SGDTENAKGQGEQGS to identify aa 511–525 of ECM1a, aa 386–400 of ECM1b, and aa 538–552 of ECM1c. An ECM1-03 antibody (Ab-03) was generated from the peptide GKEGRGPRPHSQ to test ECM1c only.

Ovarian or fallopian tissue samples from patients who were diagnosed with primary high-grade epithelial ovarian cancer or fallopian tube diseases (from whom normal fallopian or ovarian tissues were also collected, respectively) and had undergone initial surgery at Fudan University Shanghai Cancer Center between June 2013 and December 2016 were selected for this study. A total of 150 cumulative patients were identified with updated follow-up information until January 20, 2017. Histopathologic diagnoses were based on the World Health Organization criteria; tumor grades were based on the Gynecologic Oncology Group criteria. The informed consent was obtained from both patients and healthy donors whose tissues were properly collected and used in this study according to the protocols approved by the Ethics Committee of Fudan University Shanghai Cancer Center. The TMA included core samples from 30 normal human ovarian tissues and 150 human OC samples as described above. The expression levels of ECM1a, ECM1b, ECM1c, integrin αX, integrin β2, ABCG1, and hnRNPLL were detected by IHC. For IHC staining of each molecule and Kaplan–Meier survival analysis, at least 50 tissues from different cases were required in the preanalysis with 80% power at a *p* value of <0.05.

The sections were treated, stained, and scored using our previously published methods^62^. Differences in the proportions of ECM1a or ECM1b and integrin αX, integrin β2, ABCG1, or hnRNPLL expression in tumor tissues were evaluated by Fisher exact test, as appropriate. The correlations between the expression levels of each molecule in tissue arrays (based on scores for immunostaining intensity and tumor cell percentages) and patient survival were analyzed by the Kaplan–Meier method using SPSS 19.0 software (SPSS Inc.). Disease-specific survival rates were calculated using the Kaplan–Meier method and compared by log-rank test. Clinical correlations between ECM1a or ECM1b and integrin αX, integrin β2, ABCG1, or hnRNPLL expression and patient survival were examined with exclusion of missing data.

### Statistics and reproducibility

Graphs were created with GraphPad Prism. Correlations between the protein expression differences derived from IHC were analyzed by Pearson’s correlation coefficient (*r*). *t*-Test was used for quantitative analyses of various gene mRNA levels based on qRT-PCR, number of spheroids in 3D culture, cDNA bands based on semiquantative RT-PCR, mouse tumor volumes of animal assays. Fisher exact test was used for analyses of the immunostained cells and tissues. A result was considered statistically significant if the *p* value was <0.05. All statistical tests were two-sided with 95% confidential intervals. All data are reproducible with the described materials and methods.

### Reporting summary

Further information on research design is available in the Nature Research Reporting Summary linked to this article.

## Supplementary information

Supplementary Information

Peer Review File

Description of Additional Supplementary Files

Supplementary Data 1

Supplementary Data 2

Supplementary Data 3

Supplementary Data 4

Supplementary Data 5

Reporting Summary

## Data Availability

The RNA-seq data have been deposited in the NCBI-Gene Expression Omnibus (GEO) database under the accession code GSE171425. The Mass Spectrum data referenced during the study are available via ProteomeXchange with identifier PXD025239. The microscopic image files are available in the BioImage Archive with accession code S-BIAD116. All the other data supporting the findings of this study are available within the article and its Supplementary information files and from the corresponding author upon reasonable request. Source data are provided with this paper.

## Source data

Source Data
