# Supplementary material for: Extracellular matrix protein-1 secretory isoform promotes ovarian cancer through increasing alternative mRNA splicing and stemness
Source: Nat Commun. 2021 Jul 9;12:4230. doi: 10.1038/s41467-021-24315-1 (PMC8270969; doi:10.1038/s41467-021-24315-1)
Supplement: Supplementary file 1 — Supplementary Information [file 41467_2021_24315_MOESM1_ESM.pdf]

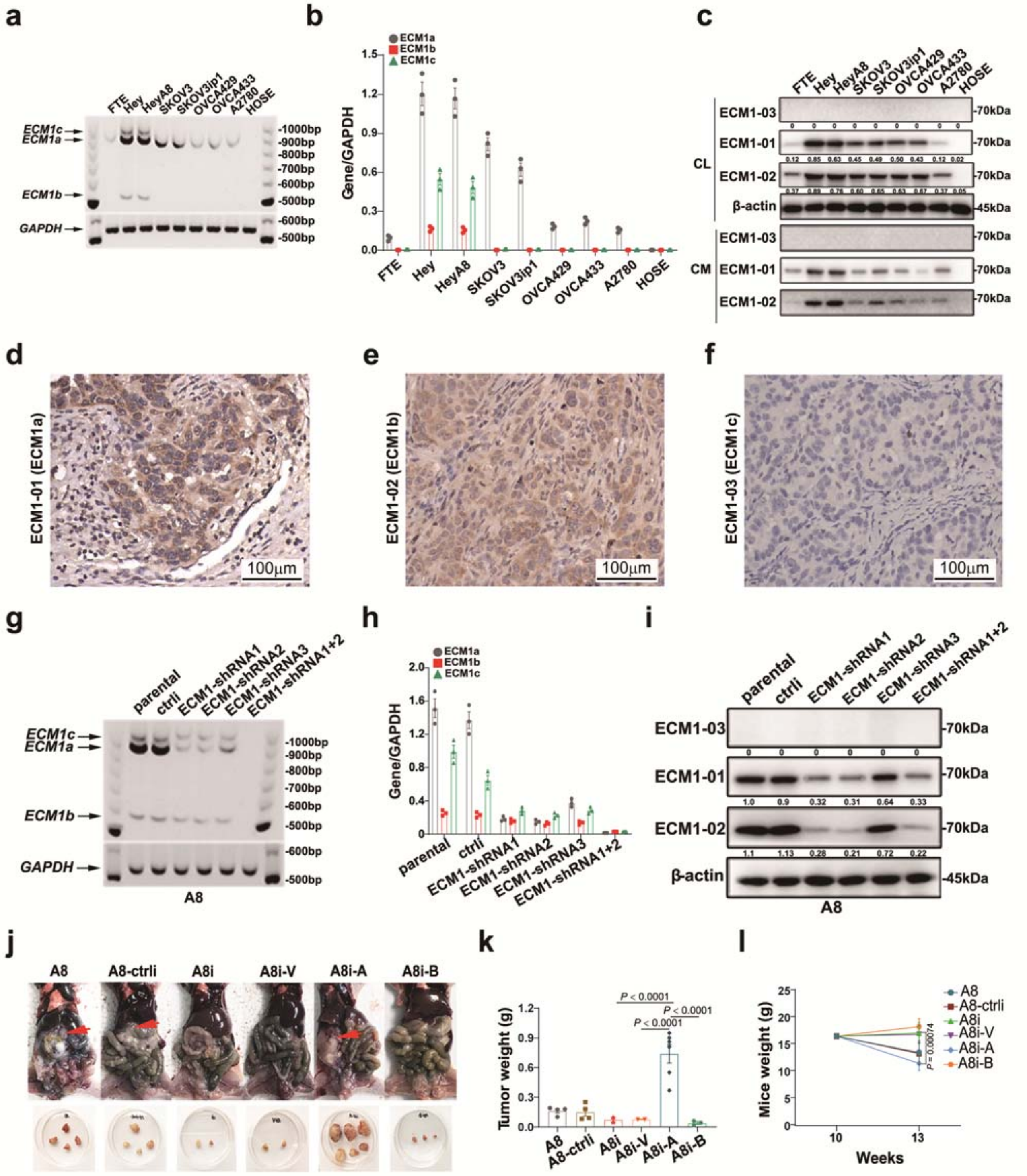

Supplementary Figure 1 Expression and tumorigenesis of ECM1 isoforms

a-c mRNA and protein levels of ECM1 isoforms in cells, CLs, and CM as detected by semiquantitative RT-PCR (**a**, representative image) and PCR product quantification (**b**, data are presented as mean ± SD, n = 3 biologically independent repeats, two-tailed *t*-test, *GAPDH* was used as an internal control), and by WB analyses (**c**) with antibodies specifically recognizing ECM1 subtypes in an FTE cell line, ovarian cancer cell lines, and a normal ovarian epithelial cell line (HOSE). **d-f** Immunostaining of ECM1a (**d**), ECM1b (**e**), and ECM1c (**f**) expression using specific antibodies by IHC in ovarian cancer tissues. Bars with 100μm indicate the tissue magnification. **g-i** mRNA and protein detection of ECM1 subtypes in cells with or without ECM1 silencing by semiquantitative RT-PCR (**g**, representative image) and PCR product quantification (**h**, data are presented as mean ± SD, n = 3 biologically independent repeats, two-tailed *t*-test, *GAPDH* was used as an internal control), and WB analysis (**i**). **j-l** Ovarian cancer cell spreading (**j**, upper panel) and nodules (**j**, lower panel) in representative xenograft mice peritoneally injected with various cells (as indicated)**.** The average tumor weights of nodules collected from five mice are shown (**k**, data are presented as mean ± SD, n = 5v5 mice, two-tailed *t*-test was calculated between A8i-A and A8i, A8i-V, or A8i-B), and the average body weights of the animals during the peritoneal tumor burden period were determined (**l**, data are presented as mean ± SD, n = 5v5 mice, two-tailed *t*-test was calculated between A8i-A and A8i-V).

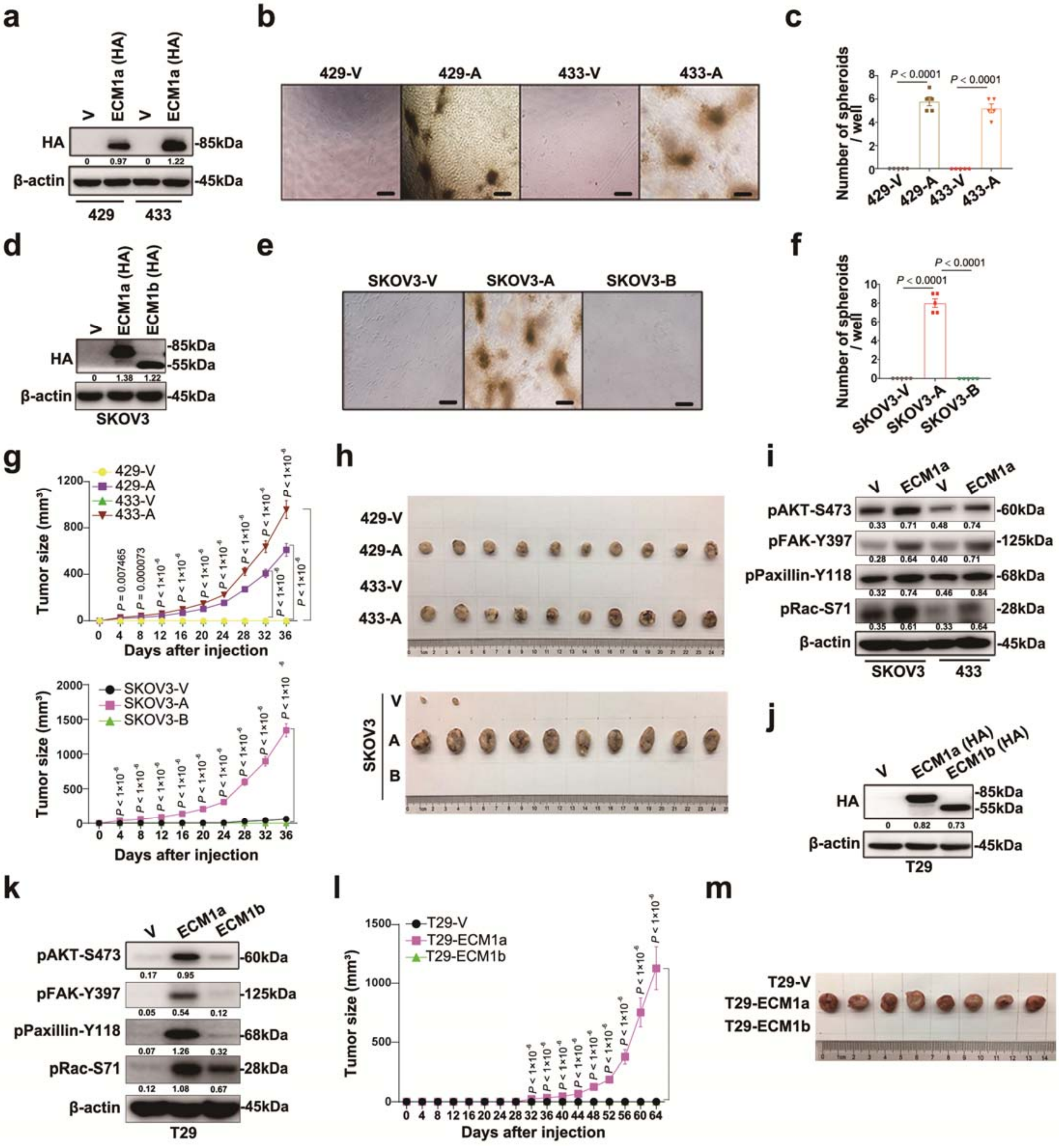

Supplementary Figure 2 Oncogenic roles of ECM1 subtypes in additional ovarian cancer cells and immortalized ovarian surface epithelial cells

a-c Overexpression of ECM1a (**a**) in the ovarian cancer cell lines OVCA429 (429) and OVCA433 (433) induced spheroid formation (**b**, bars = 400μm) and quantity (**c**, data are presented as mean ± SD, n = 5 biologically independent repeats, two-tailed *t*-test) in 3D culture. **d-f** Overexpression of ECM1a or ECM1b (**d**) promoted or inhibited spheroid formation (**e**, bars = 400μm) and quantity (**f**, data are presented as mean ± SD, n = 5 biologically independent repeats, two-tailed *t*-test) in SKOV3 cells. **g**-h Tumor growth (**g**, data are presented as mean ± SD, n = 10v10 mice, two-tailed *t*-test was calculated between 429-V and 429-A, 433-V and 433-A, or SKOV3-V and SKOV3-A) and tissues (**h**) in ten mice was promoted by ECM1a in 429, 433, and SKOV3 cells but repressed by ECM1b in SKOV3 cells compared with control cells. **i** Activation of the signaling molecules by ECM1a in SKOV3 and 433 cells. j-**m** Overexpression of ECM1a, but not ECM1b, in the immortalized ovarian surface epithelial cell line T29 (**j**) activated the same signaling molecules (**k**) identified in A8i-A cells and induced tumor growth (**l**, data are presented as mean ± SD, n = 8v8 mice, two-tailed *t*-test was calculated between T29-V and T29-ECM1a) and formation in eight mice (**m**).

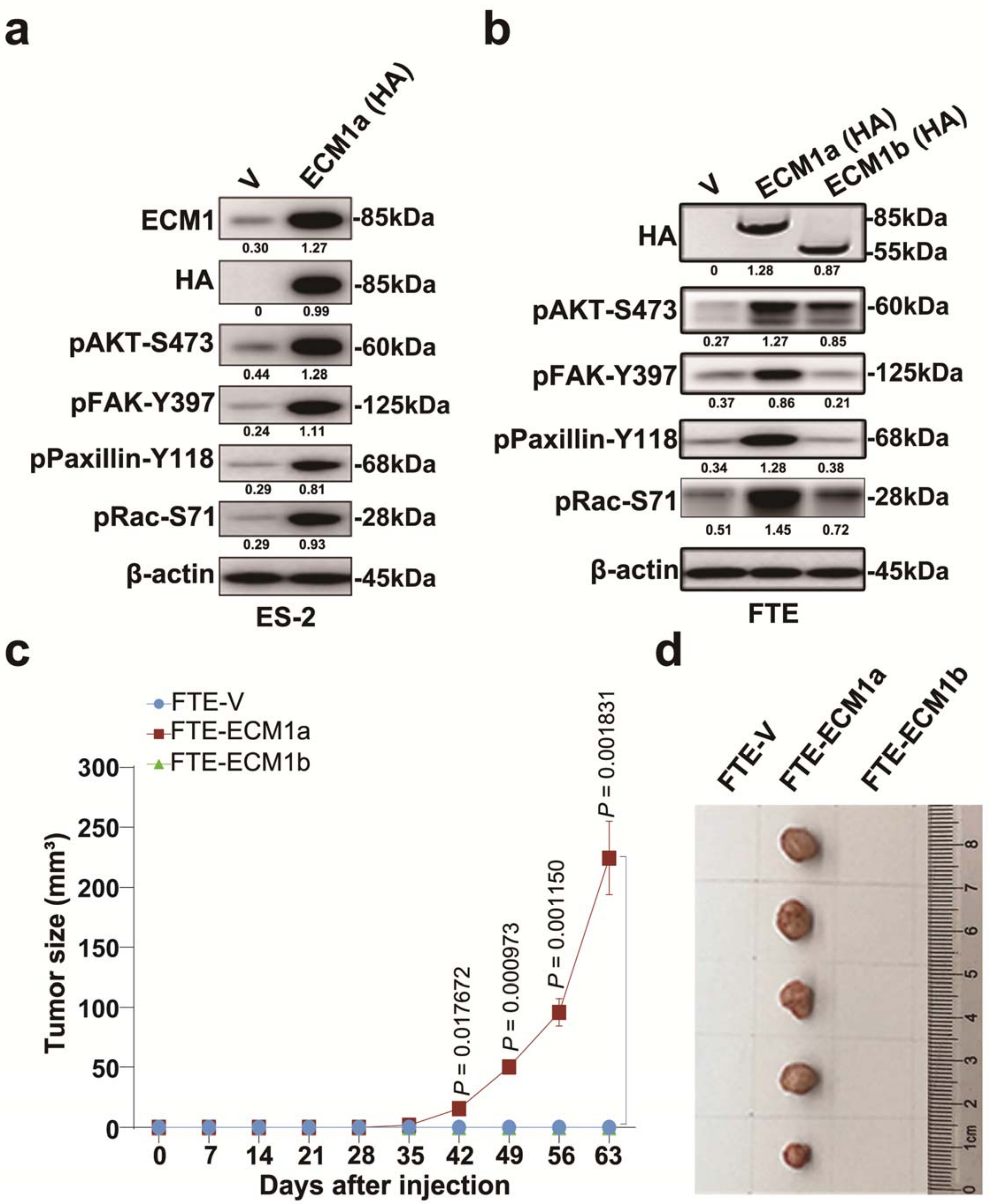

Supplementary Figure 3 Functions of ECM1a and ECM1b in ovarian clear cell carcinoma cells and FTE cells

a-d Overexpression of ECM1a or ECM1b in an ovarian clear cell carcinoma cell line (ES-2) and an immortalized FTE cell line induced or inhibited cytoskeletal molecule phosphorylation (**a-b**) and tumor growth (**c**, data are presented as mean ± SD, n = 5v5 mice, two-tailed *t*-test was calculated between FTE-V and FTE-ECM1a) and formation (**d**) in five mice injected with FTE-series cell lines.

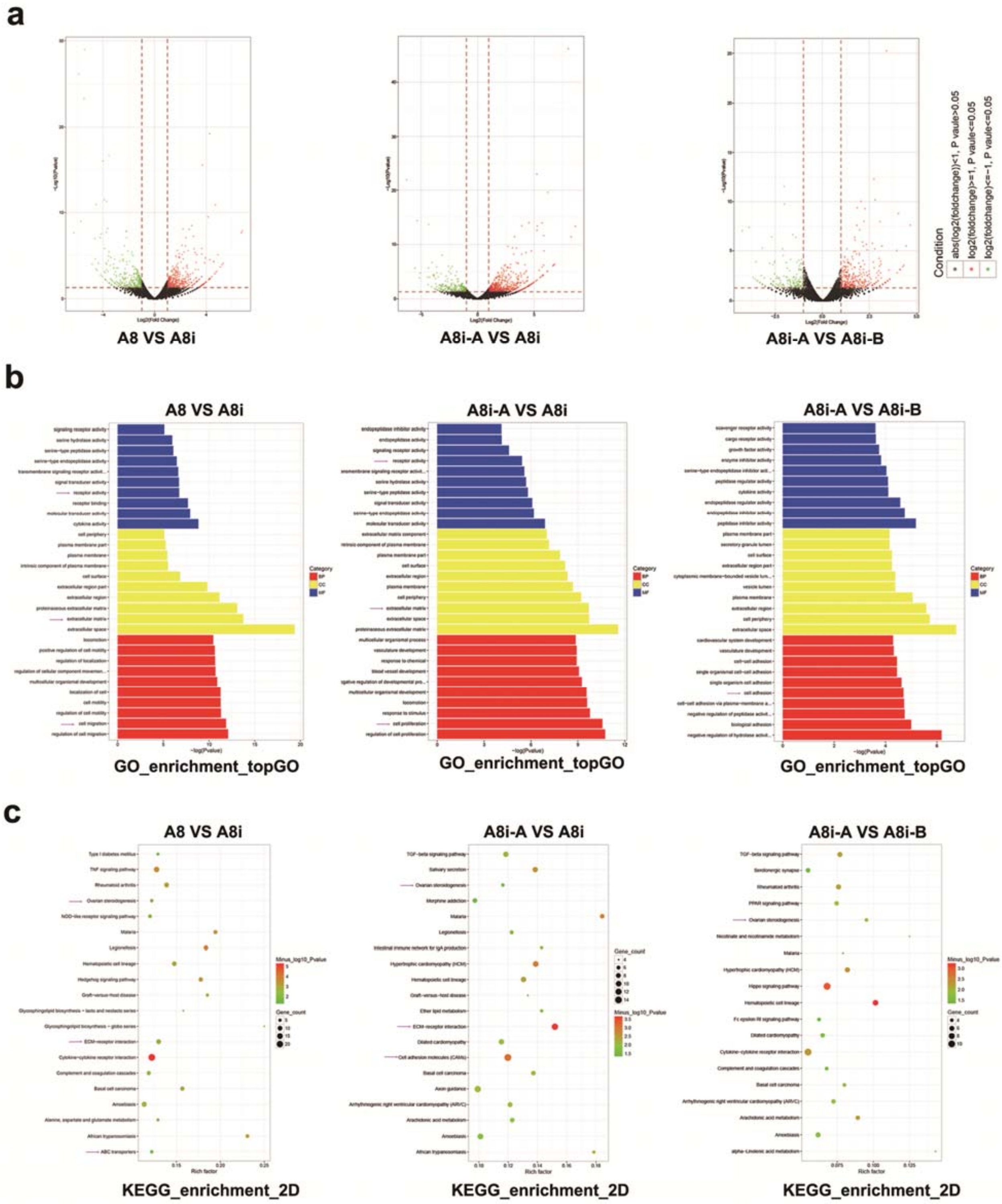

Supplementary Figure 4 RNA-seq analyses

a Volcano plots of gene expression differences in A8 cells vs. A8i cells, A8i-A cells vs. A8i cells, or A8i-A cells vs. A8i-B cells. Each point represents a gene. The red points represent significantly upregulated genes with fold changes equal to or greater than 1, the green dots represent downregulated genes with fold changes equal to or less than **-**l, and the black points are nonsignificantly changed genes. **b** Genes with differential expression in A8 cells vs. A8i cells, A8i-A cells vs. A8i cells, or A8i-A cells vs. A8i-B cells were subjected to GO analyses. The top 10 most significantly enriched biological processes (**BPs**), cellular components (**CCs**) and molecular functions (**MFs**) are shown in red, yellow and blue, respectively. The entries indicated by arrows are some of the most significantly altered cellular terms associated with ECM1 subtypes. The vertical axis displays the GO annotations corresponding to the three categories. The horizontal axis displays the -log P values corresponding to the different functional types. **c** Enrichment factor plots for the KEGG pathway enrichment analysis results. The entries indicated by arrows are some of the most altered cellular events associated with ECM1 subtypes. The degree of color represents the P value, and the size of the node represents the number of genes associated with the term. The detailed information is listed in **Supplementary Data 1-3**.

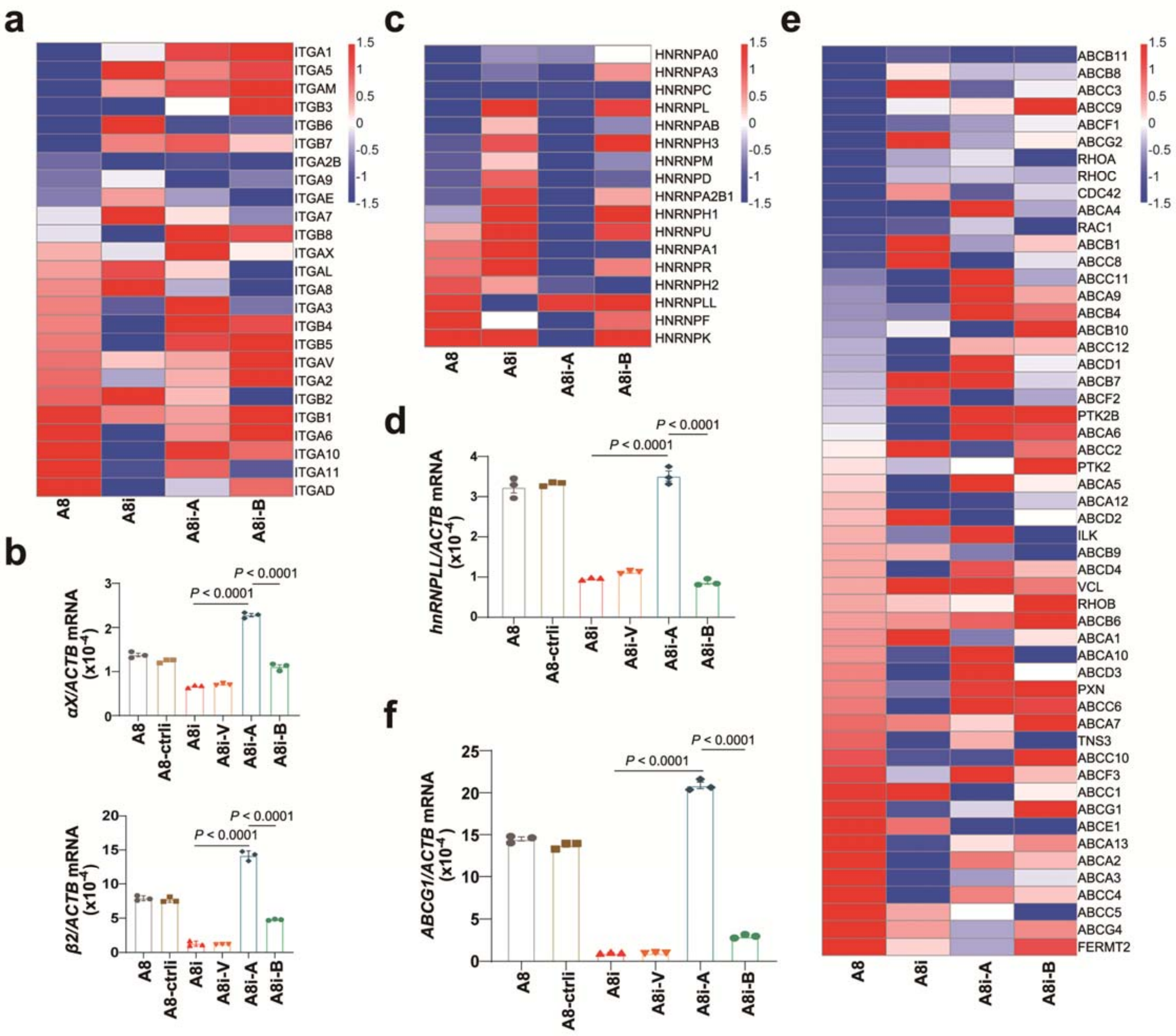

Supplementary Figure 5 Heatmaps and qRT-PCR analysis

a, c, and **e** Heatmap clusters of global patterns of integrin (ITG, **a**), hnRNP (**c**), and ABC transporter (**e**) genes determined by using the hierarchical clustering (HCL) algorithm in the four cell lines. A color map is used to distinguish the upregulated and downregulated gene transcripts in the different samples. **b, d,** and **f**. mRNA levels of integrin αXβ2, hnRNPLL, and ABCG1 as detected by qRT-PCR. *ACTB* (encoding β-actin) was detected as an internal control. Data are presented as mean ± SD, n = 3 biologically independent repeats, two-tailed *t*-test was calculated between A8i and A8i-A or A8i-A and A8i-B.

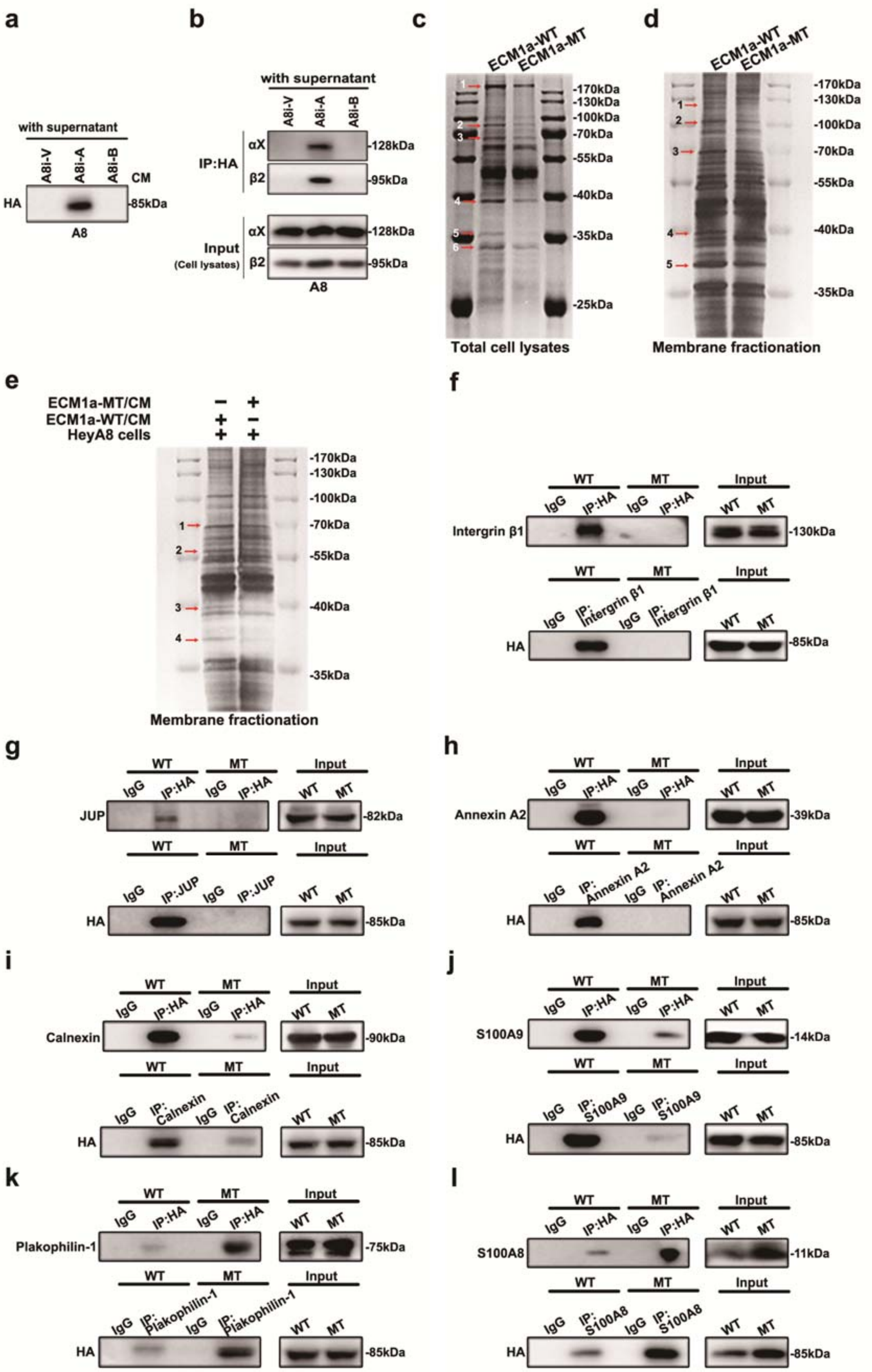

Supplementary Figure 6 Binding of ECM1 to cell surface molecules

a Detection of secreted ECM1a and ECM1b by WB analysis in CM of ECM1-silenced HeyA8 cells transfected with ECM1a and ECM1b, respectively. **b** Binding of ECM1a or ECM1b to integrin αX or integrin β2 in parental HeyA8 cells treated with the CM of ECM1a- or ECM1b-overexpressing cells. **c-d** Co-IP products prepared with an anti-HA antibody and total CLs (**c**) or cell membrane fractions (**d**) of ECM1a-WT- and ECM1a-MT-expressing cells after SDS-PAGE separation and Coomassie Brilliant Blue (R-250) staining. **e** Co-IP products prepared in the same way with cell membrane fractions of parental HeyA8 cells pretreated with the CM of ECM1a-WT- and ECM1a-MT-expressing cells. The arrows indicate the protein bands analyzed by MS. **f-l** Analyses of cell surface molecules potentially binding to ECM1a-WT or ECM1a-MT by co-IP and WB.

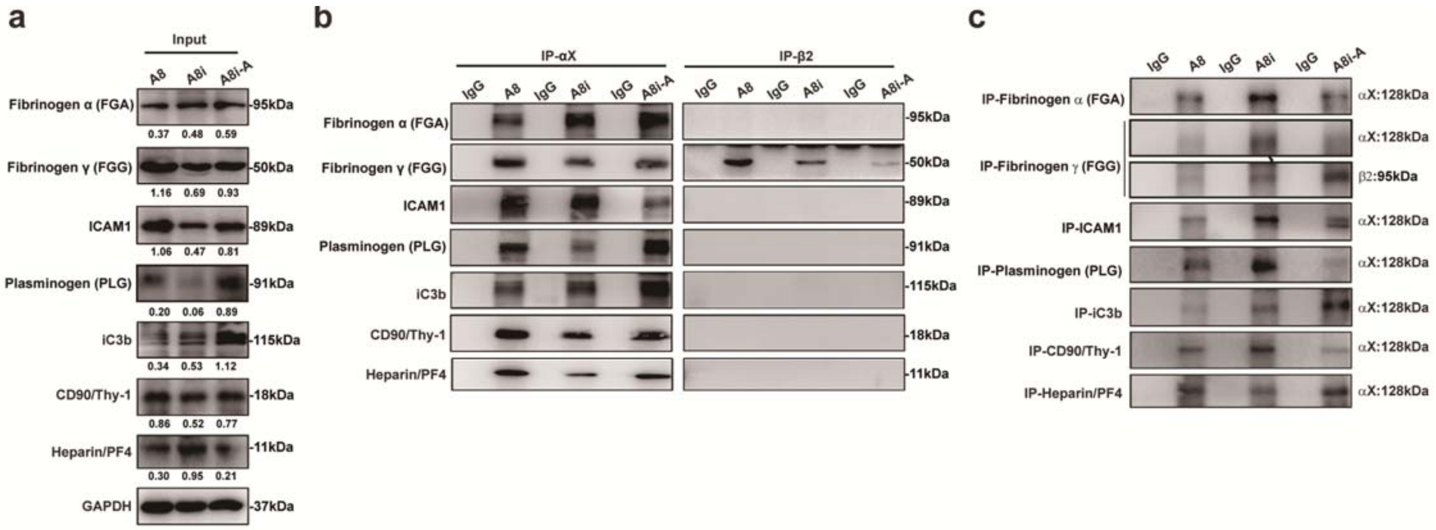

Supplementary Figure 7 Binding activity of other ligands with integrins αX and β2 in terms of the ECM1-αXβ2 interaction

a Expression levels (as the inputs of **b**) of FGA, FGG, ICAM1, PLG, iC3b, CD90/Thy-1, and heparin/PF4 in HeyA8 (A8) cells, in ECM1-silenced HeyA8 (A8i) cells, and in ECM1a-overexpressing cells after total ECM1 was silenced. **b** Binding of FGA, FGG, ICAM1, PLG, iC3b, CD90/Thy-1, and heparin/PF4 to αX (**left panel**) or β2 (**right panel**) as detected by WB in co-IP products prepared with an αX or β2 antibody. **c** Binding of αX or β2 to FGA, FGG, ICAM1, PLG, iC3b, CD90/Thy-1, and heparin/PF4 in co-IP products prepared with the corresponding antibodies against these ligands.

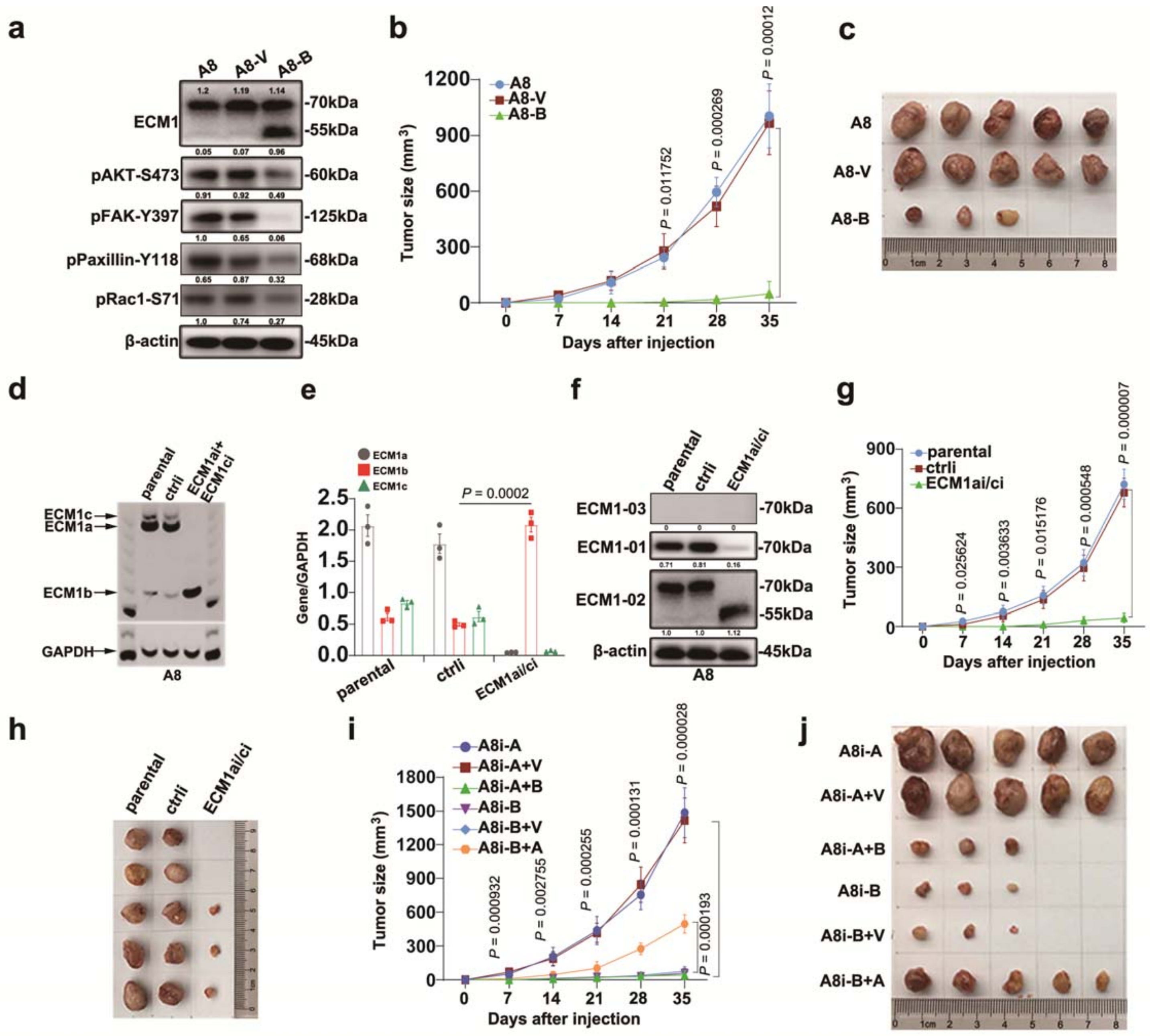

Supplementary Figure 8 Cellular signaling and tumorigenesis induced by overexpression of ECM1b and/or ECM1a or by simultaneous silencing of ECM1a and ECM1c

a-c Overexpression of ECM1b in HeyA8 cells (high endogenous ECM1a expression) suppressed cytoskeletal signaling (**a**) and tumor growth (**b**, data are presented as mean ± SD, n = 5v5 mice, two-tailed *t*-test was calculated between A8-V and A8-B) and formation (**c**) in five mice. **d-h** Silencing of *ECM1a* and *ECM1c* enhanced *ECM1b* mRNA level as detected by semi-quantitative RT-PCR in agarose gel (**d**) and quantification (**e**, data are presented as mean ± SD, n = 3 biologically independent repeats, two-tailed *t*-test was calculated between ctrli and ECM1ai/ECM1ci), and protein (**f**) detected by WB, but repressed tumor growth (**g,** data are presented as mean ± SD, n = 5v5 mice, two-tailed *t*-test was calculated between ctrli and ECM1ai/ECM1ci) and formation (**h**) in five mice. **i-j** Introduction of ECM1b or ECM1a into ECM1a- or ECM1b-overexpressing cells reversed the tumor growth (**i**, data are presented as mean ± SD, n = 5v5 mice, two-tailed *t*-test was calculated between all points of A8i-A+V and A8i-A+B <upper> or between the last points of A8i-B+V and A8i-B+A <lower>) and formation (**j**) in five mice injected with the resulting cells.

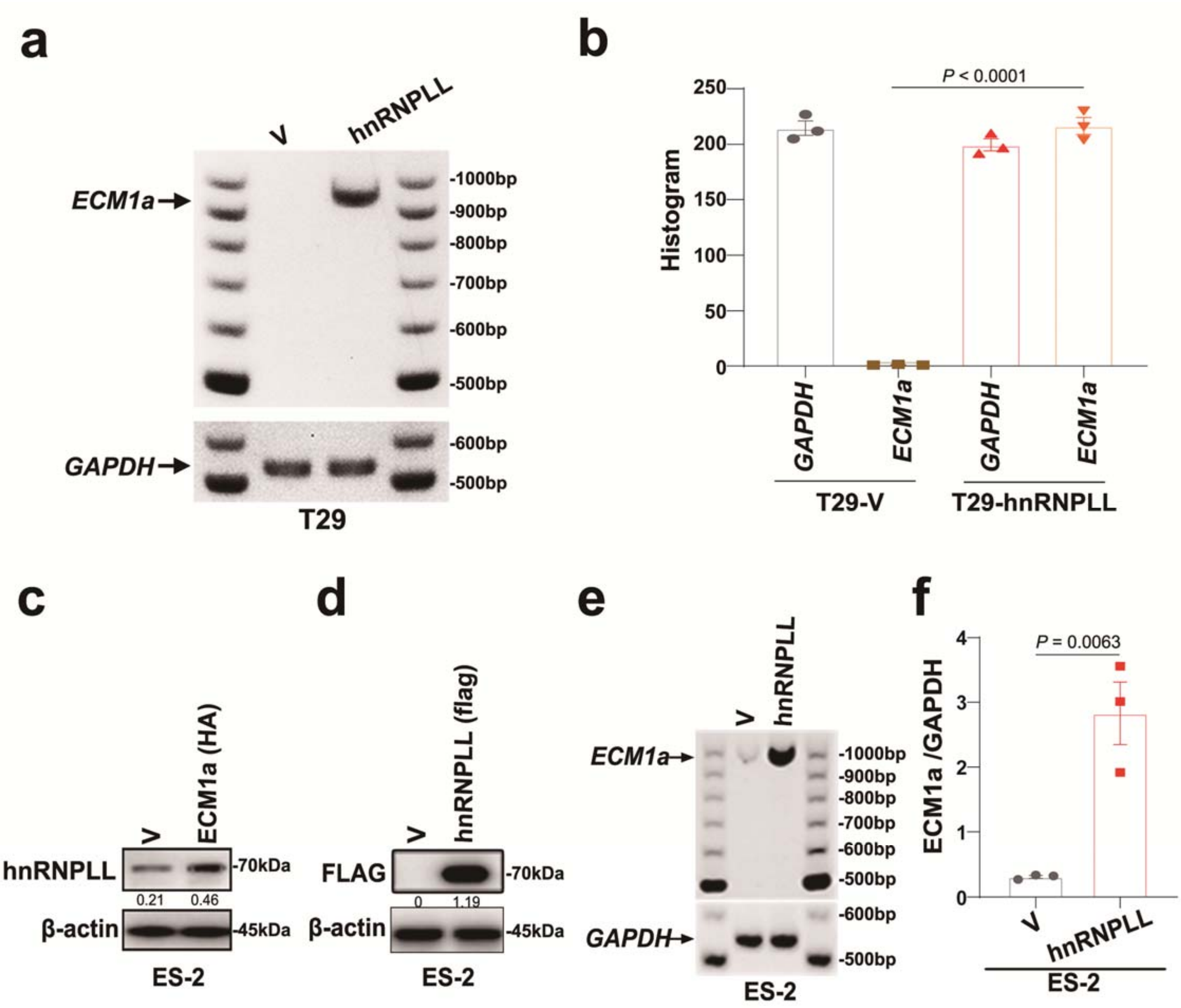

Supplementary Figure 9 hnRNPLL promotes the splicing production of ECM1a mRNA in immortalized ovarian surface epithelial cells and ovarian clear cell carcinoma cells

a-b Overexpression of hnRNPLL in T29 cells enhanced ECM1a mRNA splicing, as tested by semiquantitative RT-PCR (**a**) and quantification (**b**, data are presented as mean ± SD, n = 3 biologically independent repeats, two-tailed *t*-test was calculated between ECM1a mRNA levels of T29-V and T29-hnRNPLL cells). **c-f** Overexpression of hnRNPLL in the ovarian clear cell carcinoma cell line ES-2 (**c-d;** the actin loading control in SFig. 9c was also used for the same samples in SFig. 14j) enhanced the splicing of ECM1a mRNA as detected by semiquantitative RT-PCR in agarose gel (**e**) and quantification (**f**, data are presented as mean ± SD, n = 3 biologically independent repeats, two-tailed *t*-test).

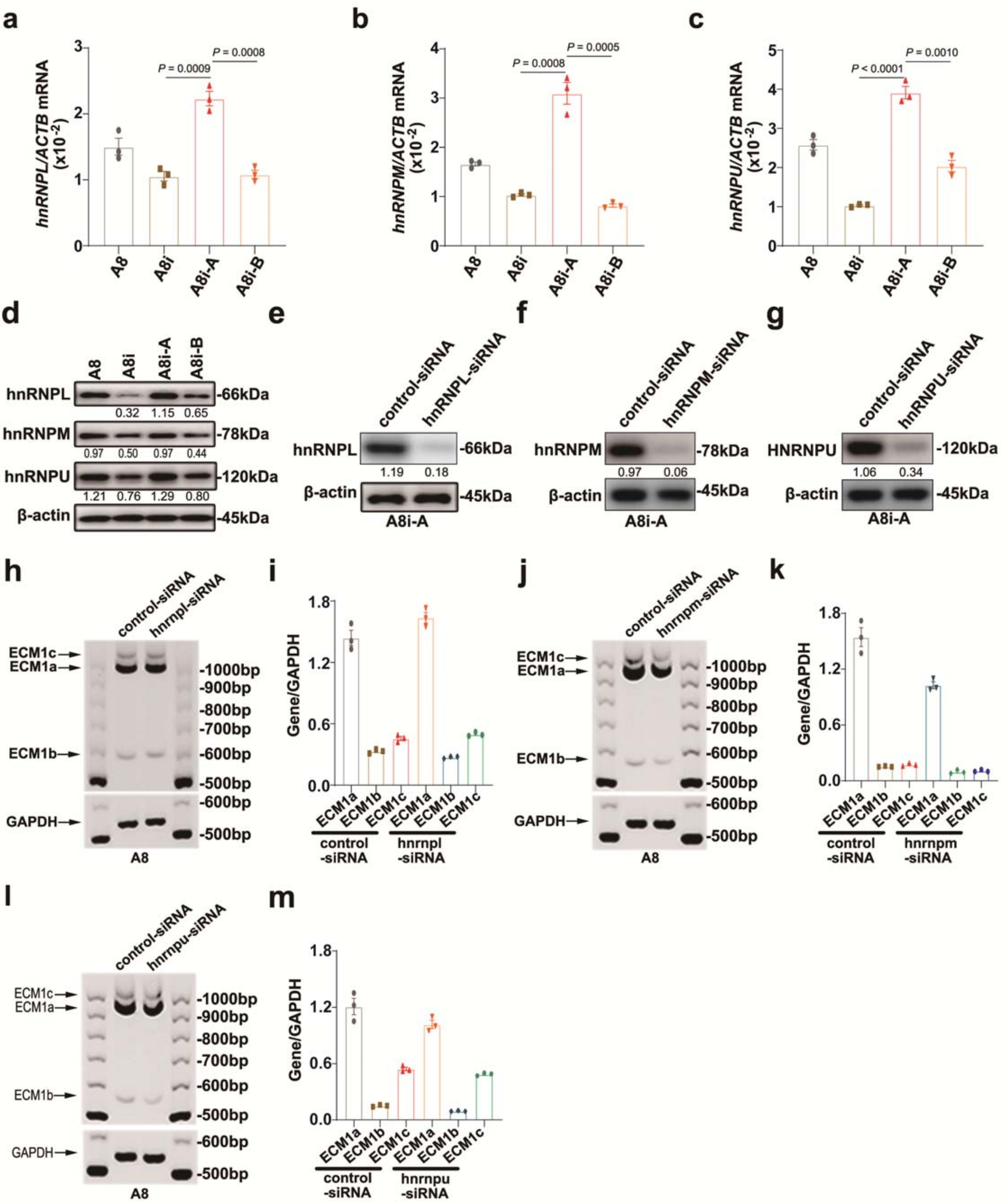

Supplementary Figure 10 hnRNPL, hnRNPM, and hnRNPU do not regulate ECM1 mRNA splicing
a-d mRNA and protein levels of hnRNPL, hnRNPM, and hnRNPU as detected by qRT-PCR (**a-c**, data are presented as mean ± SD, n = 3 biologically independent repeats, two-tailed *t*-test) and WB (**d**). **e-g** Interruption of hnRNPL (**e**), hnRNPM (**f**), and hnRNPU (**g**) expression by siRNAs. **h-m** mRNA levels of ECM1 isoforms as detected by semiquantitative RT-PCR after silencing of hnRNPL (**h-i**), hnRNPM (**j-k**), and hnRNPU (**l-m**). Data are presented as mean ± SD, n = 3 biologically independent repeats; silencing of hnRNPL, hnRNPM, and hnRNPU did not significantly alter the mRNA splicing of ECM1 isoforms.

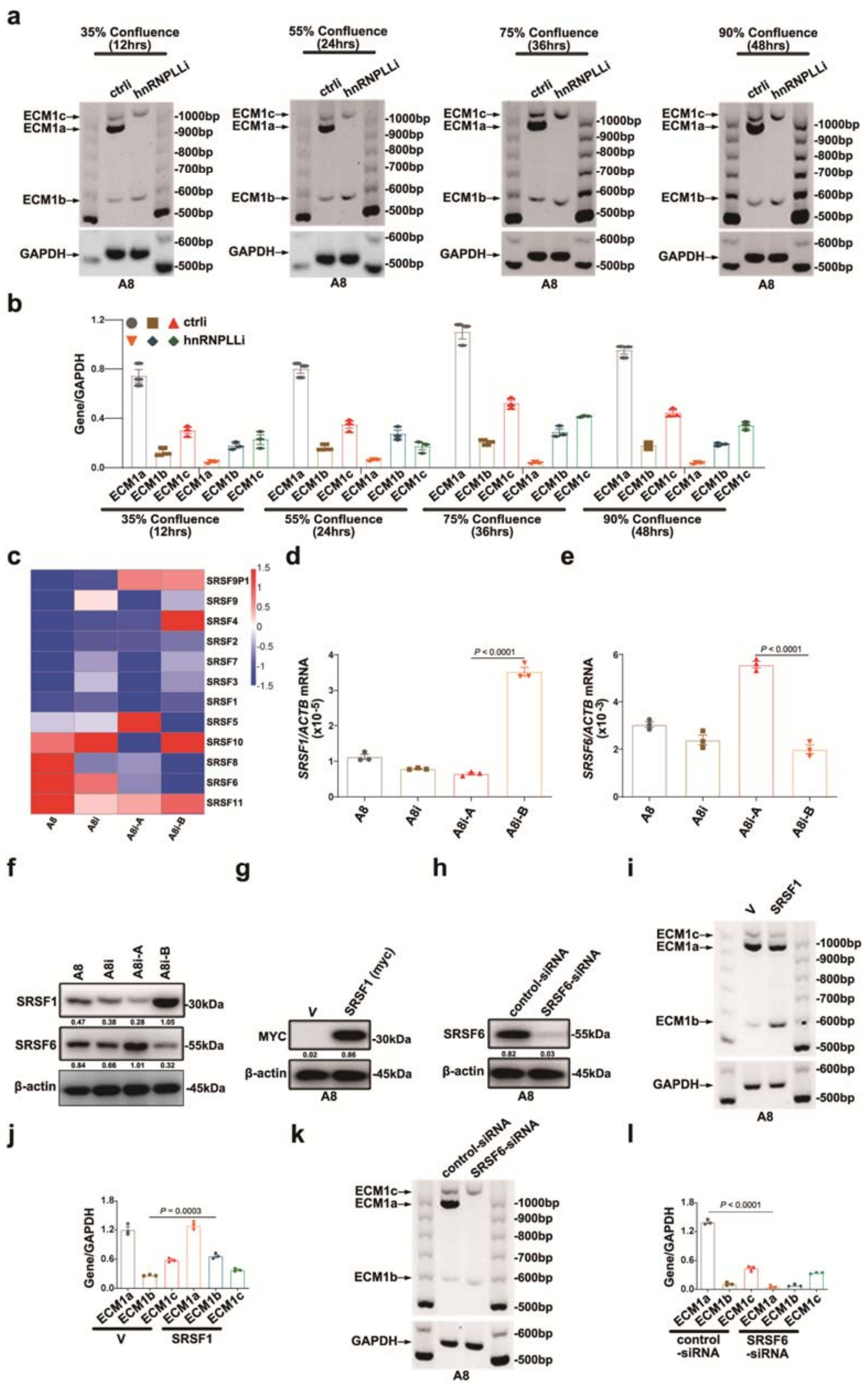

Supplementary Figure 11 mRNA regulation of ECM1 isoforms by splicing activators

a-b Silencing hnRNPLL slightly increased the mRNA levels of ECM1b and ECM1c without affecting mRNA stability during cell proliferation with progression from 35% confluence (12 hr) to 55% (24 hr), 75% (36 hr), and 90% confluence (48 hr), as detected by semiquantitative RT-PCR (**a**) and quantification (**b**, data are presented as mean ± SD, n = 3 biologically independent repeats). **c-f** ECM1a and ECM1b enhanced the splicing activators SRSF6 and SRSF1, respectively, as shown by heatmap (**c**), qRT-PCR (**d-e**, data are presented as mean ± SD, n = 3 biologically independent repeats, two-tailed *t*-test), and WB (**f**). **g-l** Overexpression of SRSF1 (**g**) or silencing of SRSF6 (**h**) upregulated ECM1b mRNA splicing (**i**) and quantity (**j**, data are presented as mean ± SD, n = 3 biologically independent repeats, two-tailed *t*-test) or downregulated ECM1a mRNA splicing (**k**) and quantity (**l**, data are presented as mean ± SD, n = 3 biologically independent repeats, two-tailed *t*-test).

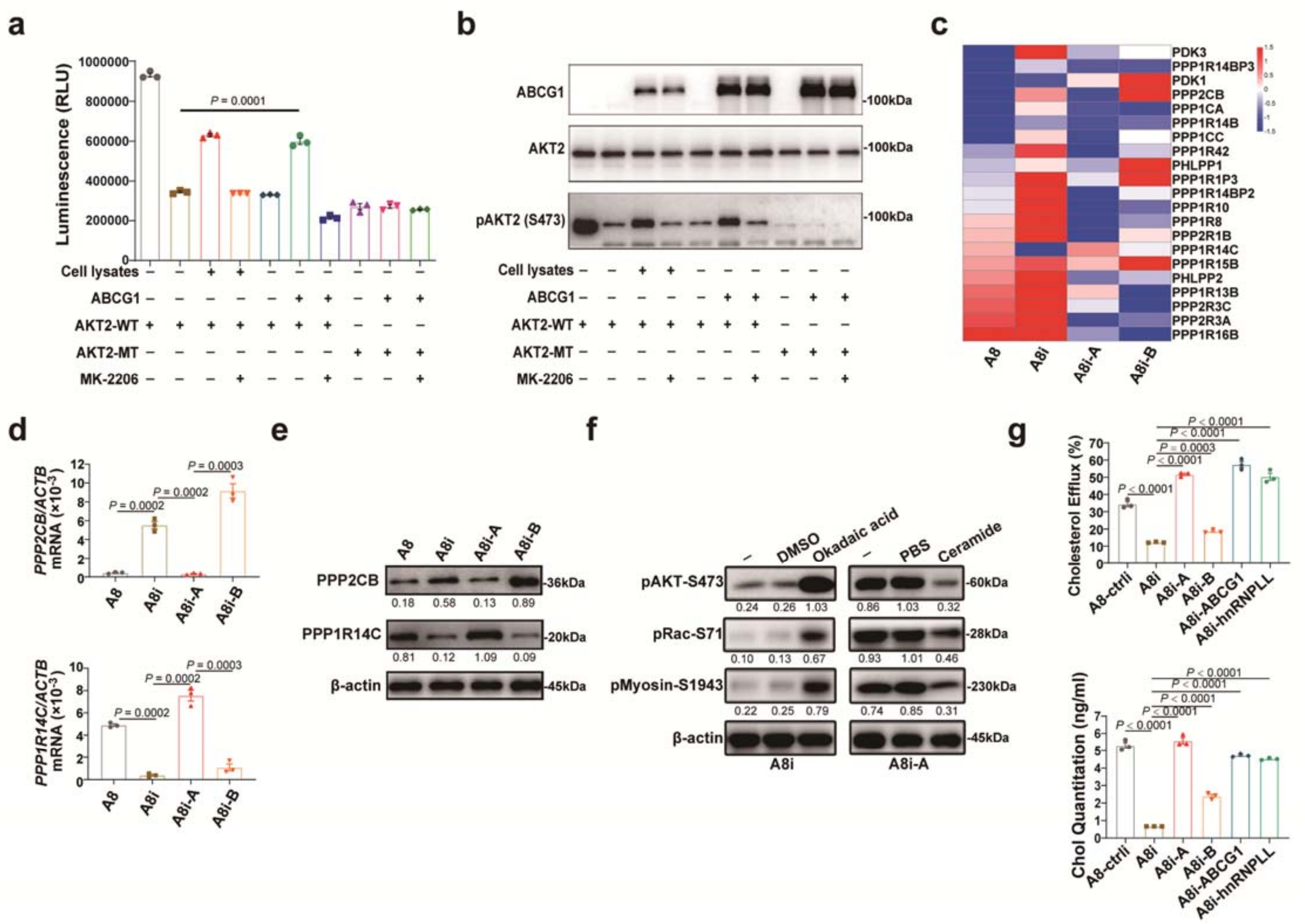

Supplementary Figure 12 Potential regulation of AKT/Rac/Myosin phosphorylation by ABCG1, protein phosphatases and phosphatase inhibitors and analysis of cholesterol alterations

a-b *In vitro* kinase assays performed with ABCG1 using AKT2 as a substrate showing the phosphorylation of AKT2 (S474), as detected by luciferase reaction (**a**, data are presented as mean ± SD, n = 3 biologically independent repeats, two-tailed *t*-test) and WB (**b**). **c-e** Based on the heatmap of the RNA-seq data (**c**), PPP2CB and PPP1R14C were either downregulated or upregulated by ECM1a or ECM1b, respectively as detected by qRT-PCR (**d**, data are presented as mean ± SD, n = 3 biologically independent repeats, two-tailed *t*-test) and WB (**e**). **f** Treatment of ECM1-silenced (A8i) or ECM1a-overexpressing (A8i-A) cells with the PP1/PP2A inhibitor okadaic acid or the PP1/PP2A activator ceramide facilitated or blocked the phosphorylation of AKT, Rac, and Myosin. **g** Quantitative analyses of cholesterol efflux (upper panel) and total cholesterol (lower panel) levels in ECM1-silenced cells and in ECM1a/1b-, ABCG1- and hnRNPLL-overexpressing cells. Data are presented as mean ± SD, n = 3 biologically independent repeats, two-tailed *t*-test.

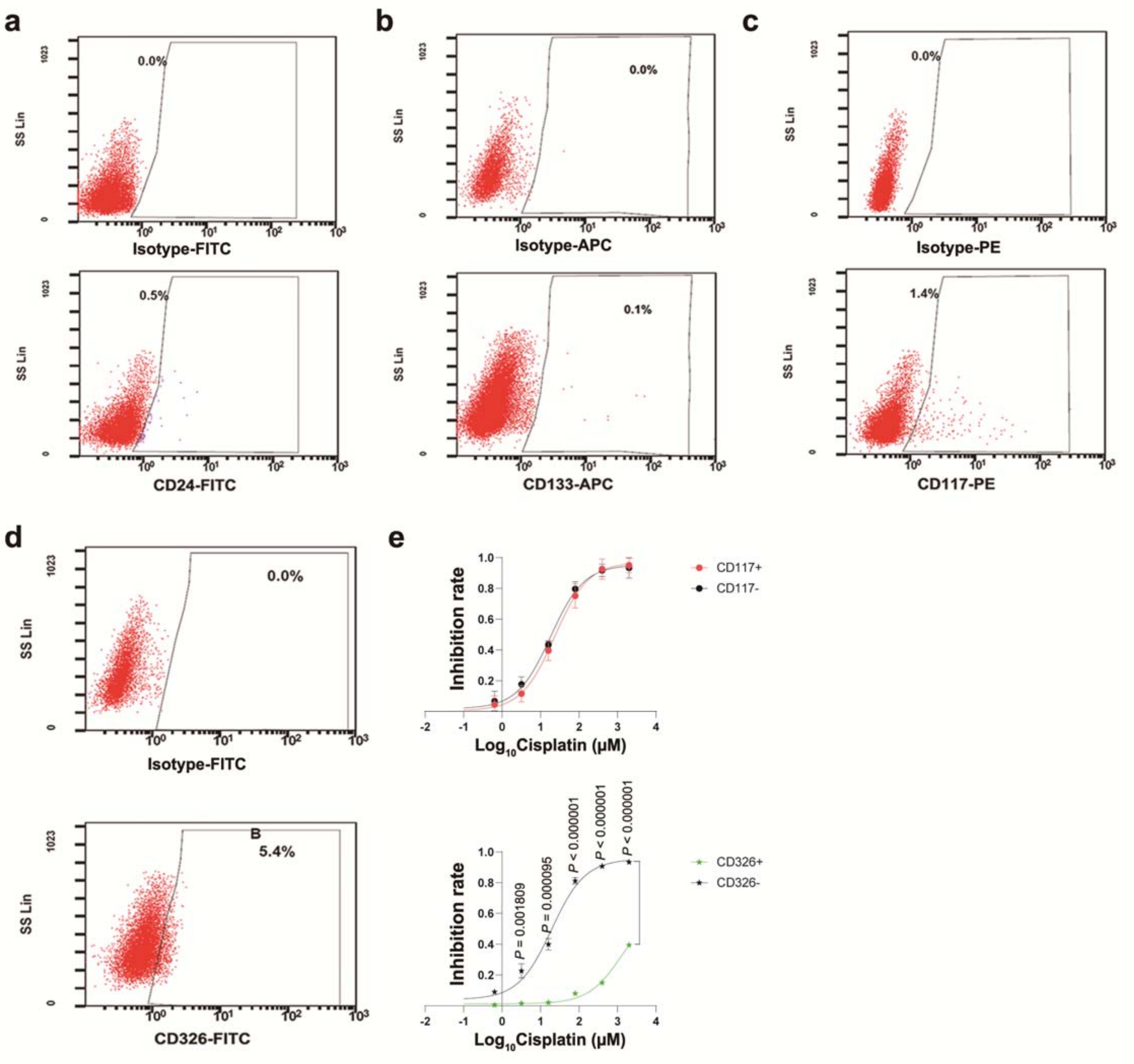

Supplementary Figure 13 Analysis of CD24+, CD133+, CD117+, and CD326+ cell populations from ABCG1-overexpressing cells by flow cytometry

a-d The population percentages of CD24+, CD133+, CD117+, and CD326+ cells were 0.5% **(a),** 0.1% **(b),** 1.4% **(c),** and 5.4% **(d)**, respectively. **e** Cisplatin inhibition rates of CD117+ and CD326+ cells. Data are presented as mean ± SD, n = 3 biologically independent repeats, two-tailed *t*-test.

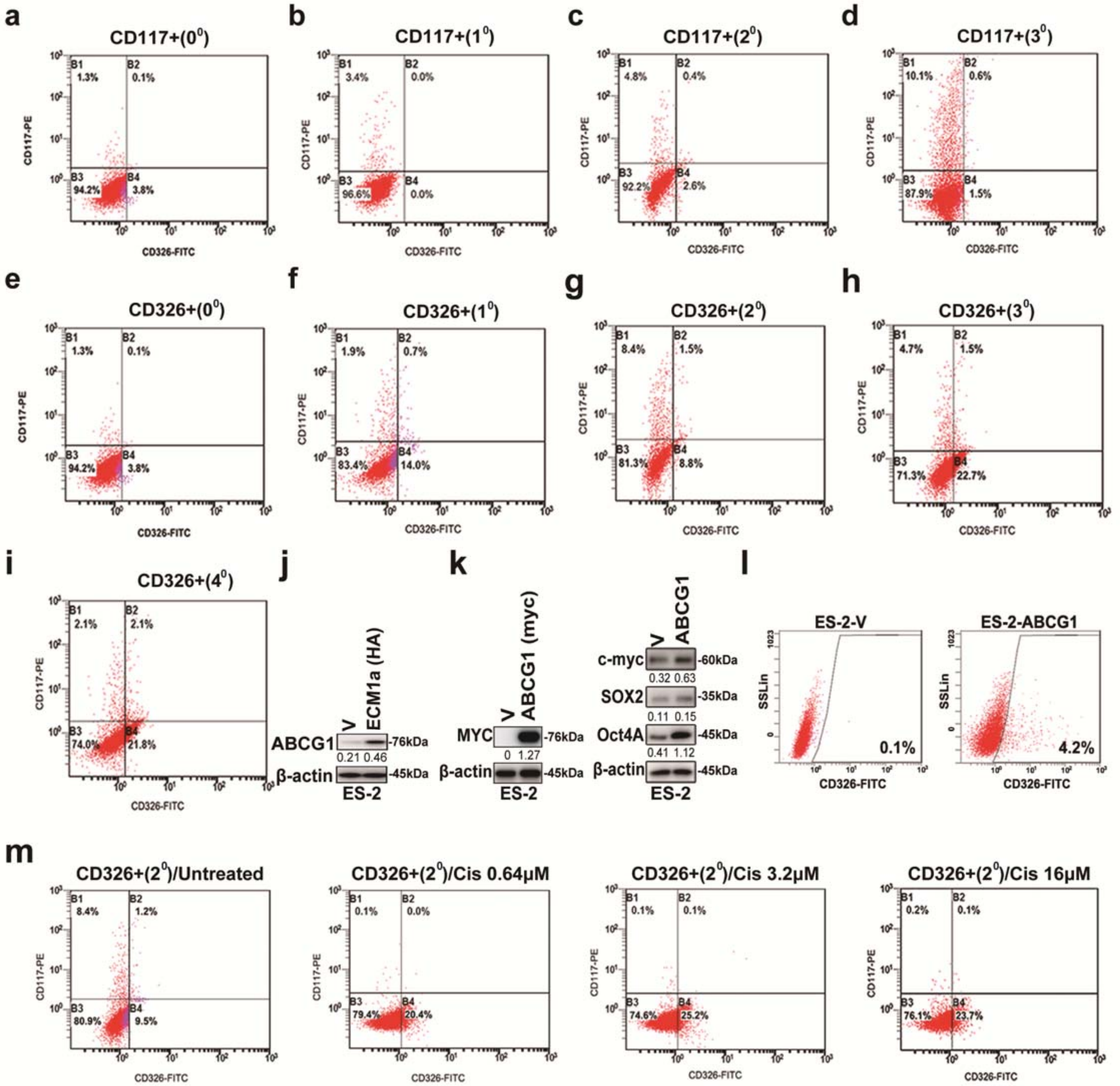

Supplementary Figure 14 Analysis of CD117+ or CD326+ cells by flow cytometry after selection-and-culture cycles or low-dose cisplatin treatment

a-d Analysis of CD117+ cells after three selection-and-culture cycles of CD117+ cells by flow cytometry. **e-i** Analyses of CD326+ cells after four selection-and-culture cycles of CD326+ cells by flow cytometry. **j-l** Introduction of ECM1a upregulated ABCG1 expression (**j;** the actin loading control was also used for the same samples in **SFig. 9c**), whereas overexpression of ABCG1 in ES-2 cells promoted cell stemness transcription factor expression (**k**) and enriched the CD326+ cell population (**l**). **m** Analysis of the CD326+ cell population from CD326+ cells pretreated with low concentrations of cisplatin at 0.64, 3.2, and 16 μM.

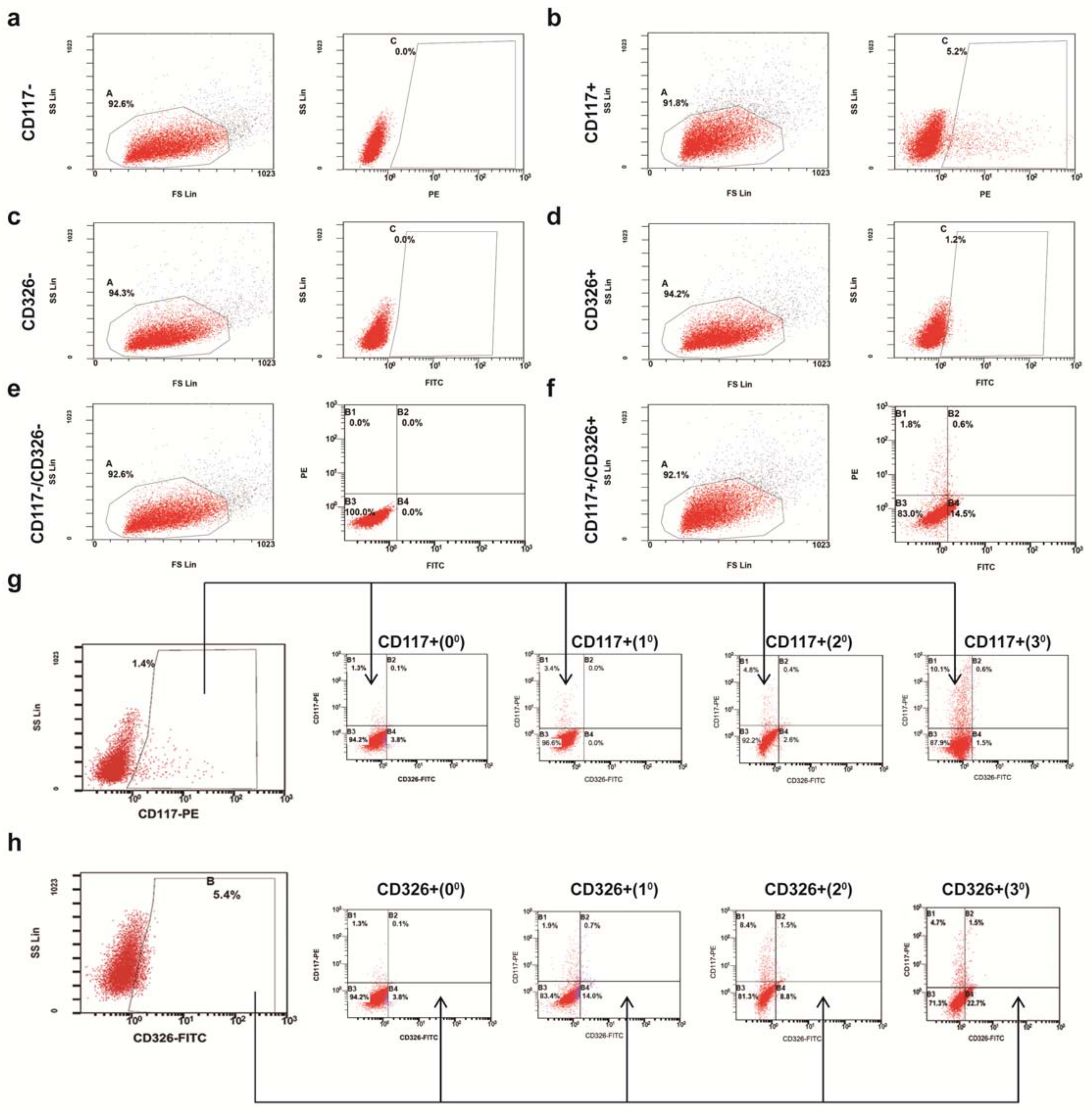

Supplementary Figure 15 Figures exemplifying the gating strategy used on a flow cytometer equipped with MXP software

a-b Images exemplifying the gating strategy of CD117- cells (**a**) and CD117+ cells (**b**) for **Supplementary Figure 13 c**. **c-d** Images exemplifying the gating strategy of CD326- cells (**c**) and CD326+ cells (**d**) for **Supplementary Figure 13d** and **Supplementary Figure 14l**. **e-f** Figures exemplifying the sorting strategy of CD117-/CD326- cells (**e**) and CD117+/CD326+ cells (**f**) for **Supplementary Figure 14 a** & **e**. **g-h** Images exemplifying the sorting strategy of CD117/CD326 cell population from three culture-selection cycles of CD117+ cells (**g**) or four culture-selection cycles of CD326+ cells (**h**) for **Supplementary Figure 14b-d, f-i** and **m**. The data in all figures were analyzed with MXP software coupled to the flow cytometer.

Supplementary Table 1. Additional cell surface molecules other than integrin αXβ2 binding to ECM1-WT or ECM1-MT identified by mass-spectrum

**Supplementary Figure 6c gel**		
**Common**	**Difference**	**Difference**
W1/M1*	W1	M1
Myosin 9 , Keratin 1, Filamin A, B	Myosin 10,11,18A, Desmoplakin, Junction plakoglobin, Filaggrin-2	Talin
W2/M2	W2	M2
Keratin, Gelsolin	CD71, Actinin alpha 4, Calnexin	
W3/M3	W3	M3
Keratin, Moeson, Epididymis	ECM1, Desmoplakin	Desmoplakin, Hornerin, Junction plakoglobin, Dermcidin
W4/M4	W4	M4
Myosin	Desmoplakin, Filaggrin, Serpin B3, Desmoglein-1, Annexin 2, Galectin-7, S100	
W5/M5	W5	M5
Keratin, Myosin, Desmoplakin		Annexin A2
W6/M6	W6	M6
Keratin, Annexin A1/A2, Dermcidin		Desmoplakin, Plakophilin-1
**Supplementary Figure 6d gel**		
**Common**	**Difference**	**Difference**
W1/M1	W1	M1
Keratin 1,dermcidin, Hsp90	Integrin b1	Integrin a3
W2/M2	W2	M2
Keratin 1, Desmoplakin, Epididymis, Hornerin,	Annexin A2	

Dermcidin, Junction Plakoglobin		
W3/M3	W3	M3
Keratin 1, Dermcidin, Hornerin, Desmoplakin, Desmoglein-1	ECM1	
W4/M4	W4	M4
Keratin 1, S100-A9, Desmoplakin, Annexin a2, Demcidin, Desmoglein-1	Annexin a7	S100-A8
w5/m5	W5	M5
Keratin 1, Dermcidin, Desmoplakin		Desmoglein-1
**Supplementary Figure 6e gel**		
**Common**	**Difference**	**Difference**
W1/M1	W1	M1
Keratin 1, Endoplasmic reticulum chaperone BiP, Desmoplakin, Hornerin	Moesin	S100, S100-a9, Junction plakoglobin, Annexin a2
W2/M2	W2	M2
Keratin 1, Desmoplakin, Myosin 3	Myosin 1, 4, 6, 7, 8, Dermcindin	Ixa, Desmoglein-1, Junction plakoglobin isoform 1, Annexin a2
W3/M3	W3	M3
Keratin 1, Hornerin, Desmoplakin, S100	S100-A9,	Annexin a2
W4/M4	W4	M4
Keratin 1, Dermcidin	Desmoglein-1	Desmoplakin, Desmoglein-1, S100, S100-a9, Annexin a2

***W indicates ECM1a-WT samples; M indicates ECM1a-MT samples.**

Supplementary Table 2. The genes/proteins from the RNA sequencing that may interact with integrin αXβ2

Gene_ID	Symbol	A8	A8i	A8i-A
ENSG00000171560	FGA	4.326663	3.360172	4.400572
ENSG00000171557	FGG	0.871012	2.929876	0
ENSG00000090339	ICAM1	10.34976	11.38996	11.89356
ENSG00000122194	PLG	3.080745	3.161007	3.688112
ENSG00000125730	C3	9.141298	12.69696	12.61412
ENSG00000154096	CD90/Thy-1	/*	/	/
ENSG00000163737	PF4	/	/	/

*not available in the database

Supplementary Table 3. Correlation of ECM1a expression with other molecules in the TMA

Markers	Pearson's correlation (r)	#*P* value
integrin αX	0.181	0.03
integrin β2	0.403	< 0.001
hnRNPLL	0.204	0.014
ABCG1	0.392	< 0.001

#Two-sided Fisher exact test is used for statistical analysis.

Supplementary Table 4. Correlation of ECM1b expression with other molecules in the TMA

Markers	Pearson's correlation	#*P* value
Integrin αX	-0.248	0.003
Integrin β2	-0.207	0.013
hnRNPLL	-0.294	< 0.001
ABCG1	-0.356	< 0.001

#Two-sided Fisher exact test is used for statistical analysis.

Supplementary Table 5. Expression level correlations between ECM1a or ECM1b and other molecules in the TMA

		ECM1a				ECM1b			
		Low	High	Total	**P*	Low	High	Total	**P*
Integrin αX	Low	28	16	44		27	17	44	
	High	44	56	100	0.0139	84	16	100	0.0025
Integrin β2	Low	51	22	73		50	23	73	
	High	21	50	71	< 0.001	61	10	71	0.007
hnRNPLL	Low	54	40	94		64	30	94	
	High	18	32	50	0.007	47	3	50	<0.001
ABCG1	Low	35	9	44		24	20	44	
	High	37	63	100	< 0.001	87	13	100	<0.001

*Two-sided Fisher exact test was used for statistical analysis.

Supplementary Table 6. Primers/oligoes used/ordered in the study

No.	Names	Sequences and notes	Purposes
1	ECM1-sh-1FW	5'-ccgg**AGAGCCATCCAGAACCTGAGT**ctcgag**ACTCAGGTTCTGGATGGCTCT**tttttg-3' targeting ECM1 CDS mRNA 410-430nt (ECM1a)	Silencing of ECM1
2	ECM1-sh-1RV	5′-aattcaaaaaa**AGAGCCATCCAGAACCTGAGT**ctcgag**ACTCAGGTTCTGGATGGCTCT-**3′	Silencing of ECM1
3	ECM1-sh-2FW	5′-ccgg**ATTTCCTGGAGATTGGATATT**ctcgag**AATATCCAATCTCCAGGAAATttttt**G-3′ targeting ECM1 CDS mRNA 632-652nt (ECM1a)	Silencing of ECM1
4	ECM1-sh-2RV	5′-aattcaaaaaa**ATTTCCTGGAGATTGGATATT**ctcgag**AATATCCAATCTCCAGGAAAT-**3′	Silencing of ECM1
5	ECM1-sh-3FW	5′-ccgg**GAGTTCTCACCAAGCATAAAC**ctcgag**GTTTATGCTTGGTGAGAACTC**tttttg-3′targeting ECM1 CDS mRNA 1289-1309nt (ECM1a)	Silencing of ECM1
6	ECM1-sh-3RV	5′-aattcaaaaaa**GAGTTCTCACCAAGCATAAAC**ctcgag**GTTTATGCTTGGTGAGAACTC-**3′	Silencing of ECM1
7	ECM1-FW	5′-ataatTCTAGAGCTAGCGAATTC**atggggaccacagccagagcag**-3′ with *Xba* I/*Nhe* I/*Eco* R I sites	ECM1 cDNA cloning
8	ECM1-RV	5′-ataatGCGGCCGCGGATCCtca*AGCGTAGTCTGGGACGTCGTATGGGTA***ttcttccttgggctcagagg**-3′ with *Not* I/*Bam*H I sites and HA-tag	ECM1 cDNA cloning
9	ECM1a-GPR-FW	5′-atcaatgatctgtgtg**T**t**G**ccc**A**acgtaacatctggcga-3′	ECM1a mutation
10	ECM1a-GPR-RV	5′-tcgccagatgttacgt**T**ggg**C**a**A**cacacagatcattgat-3′	ECM1a mutation
11	ECM1-Q-FW	5′- CTGTGACCTGCCATTTCCAGA-3′	ECM1 qPCR
12	ECM1-Q-RV	5′- CCAGACACTAGAGCCACGTTCC-3′	ECM1 qPCR
13	ITG αX shRNA-1FW	5′-ccgg**AACCAACTGAAGGAGAAGATC**ctcgag**GATCTTCTCCTTCAGTTGGTT tttttg**-3′ targeting αX CDS mRNA 979-1001nt	Silencing of αX
14	ITG αX shRNA-1RV	5′-aattcaaaaa**AACCAACTGAAGGAGAAGATC**ctcgag**GATCTTCTCCTTCAGTTGGTT-**3′	Silencing of αX
15	ITG αX shRNA-2FW	5'-ccgg**GGGTGCTGTCTACCTGTTTCA**ctcgag**TGAAACAGGTAGACAGCACCC**tttttg-3' targeting αX CDS mRNA 1650-1670nt	Silencing of αX
16	ITG αX shRNA-2RV	5′-aattcaaaaa**GGGTGCTGTCTACCTGTTTCA**ctcgag**TGAAACAGGTAGACAGCACCC-**3′	Silencing of αX
17	ITG αX shRNA-3FW	5′-ccgg**TGCCACCTTCCAGGAAACAAA**ctcgag**TTTGTTTCCTGGAAGGTGGCA**tttttg-3′ targeting αX CDS mRNA 2067-2087nt	Silencing of αX
18	ITG αX shRNA-3RV	5′-aattcaaaaa**TGCCACCTTCCAGGAAACAAA**ctcgag**TTTGTTTCCTGGAAGGTGGCA-**3′	Silencing of αX
19	ITG αX-Q-FW	5′-CAACACTCCCAGGACCAGCA-3′	integrin αX qPCR
20	ITG αX-Q-RV	5′-GCATGGCCACATGGCTTTC-3′	integrin αX qPCR
21	ITG β2 shRNA-1FW	5′-ccgg**TGCTGAAGCTGACCAACAACT**ctcgag**AGTTGTTGGTCAGCTTCAGCA**tttttg-3′ targeting β2 CDS mRNA 620-640nt	Silencing of β2
22	ITG β2 shRNA-1RV	5′-aattcaaaaa**TGCTGAAGCTGACCAACAACT**ctcgag**AGTTGTTGGTCAGCTTCAGCA-**3′	Silencing of β2
23	ITG β2 shRNA-2FW	5′-ccgg**TGGAGGACAACTTGTACAAGA**ctcgag**TCTTGTACAAGTTGTCCTCCA**tttttg-3′ targeting β2 CDS mRNA 863-883nt	Silencing of β2

24	ITG β2 shRNA-2RV	5′-aattcaaaaa**TGGAGGACAACTTGTACAAGA**ctcgag**TCTTGTACAAGTTGTCCTCCA**-3′	Silencing of β2
25	ITG β2 shRNA-3FW	5′-ccgg**GTGTGACACCATCAACTGTGA**ctcgag**TCACAGTTGATGGTGTCACAC**tttttg-3′ targeting β2 CDS mRNA 1605-1625nt	Silencing of β2
26	ITG β2 shRNA-3RV	5′-aattcaaaaa**GTGTGACACCATCAACTGTGA**ctcgag**TCACAGTTGATGGTGTCACAC**-3′	Silencing of β2
27	ITG β2-Q-FW	5′- ACTCCAGCAATGTGGTCCATCTC-3′	integrin β2 qPCR
28	ITG β2-Q-RV	5′-GATCTGCACGCCATCACAGTC-3′	integrin β2 qPCR
29	hnRNPLL-FW	5′- gattaTCTAGA**atgttggctcgggagacgtacgag**-3′ with *Xba*I I site	hnRNPLLcDNA cloning
30	hnRNPLL-RV	5′-GCGC**GAATTC**tta*CTTGTCGTCATCGTCTTTGTAGTC***taaatgggatgatgtagaaaagc**-3′with *Eco*R I site and Flag-tag	hnRNPLLcDNA cloning
31	hnRNPLL shRNA-1FW	5′-ccgg**GCACTGAATCACTATCAGATA**ctcgag**TATCTGATAGTGATTCAGTGC**tttttg-3′ targeting hnRNPLL CDS mRNA 1543-1563 nt (NM_138394.3)	Silencing of hnRNPLL
32	hnRNPLL shRNA-1RV	5′-aattcaaaaa**GCACTGAATCACTATCAGATA**ctcgag**TATCTGATAGTGATTCAGTGC**-3′	Silencing of hnRNPLL
33	hnRNPLL shRNA-2FW	5′-ccgg**GCAAAGTGCAACGTATTGTTA**ctcgag**TAACAATACGTTGCACTTTGC**tttttg-3′ targeting hnRNPLL CDS mRNA 578-598 nt (NM_138394.3)	Silencing of hnRNPLL
34	hnRNPLL shRNA-2RV	5′-aattcaaaaa**GCAAAGTGCAACGTATTGTTA**ctcgag**TAACAATACGTTGCACTTTGC**-3′	Silencing of hnRNPLL
35	hnRNPLL shRNA-3FW	5′-ccgg**CGACAGGCTCTAGTGGAATTT**ctcgag**AAATTCCACTAGAGCCTGTCG**tttttg-3′ targeting hnRNPLL CDS mRNA 334-354 nt (NM_138394.3)	Silencing of hnRNPLL
36	hnRNPLL shRNA-3RV	5′-aattcaaaaa**CGACAGGCTCTAGTGGAATTT**ctcgag**AAATTCCACTAGAGCCTGTCG**-3′	Silencing of hnRNPLL
37	HnRNPLL-Q-FW	5′-CTACAAGCAAAAGGATCACTCG-3′	hnRNPLL qPCR
38	HnRNPLL-Q-RV	5′-CCACTGTAATTGGATAAAGCGG-3′	hnRNPLL qPCR
39	Abcg1-FW	5′- gattcTCTAGA**atggcctgtctgatggccgctttct**-3′ with *Xba* I site	Abcg1 cDNA cloning
40	Abcg1-RV	5′-GCGT**GGATCC**tta*CAGATCCTCTTCTGAGATGAGTTTTTGTTC***cctctctgcccggattttgtacc**-3′ with *Bam* HI site and Myc-tag	Abcg1 cDNA cloning
41	Abcg1 shRNA-1FW	5'-ccgg**GAAGTTCAATAGTGGTGAGTT**ctcgag**AACTCACCACTATTGAACTTC**tttttg-3′targeting Abcg1 CDS mRNA 324-344 nt (V1)	Silencing of Abcg1
42	Abcg1 shRNA-1RV	5′-aattcaaaaa**GAAGTTCAATAGTGGTGAGTT**ctcgag**AACTCACCACTATTGAACTTC**-3′	Silencing of Abcg1
43	Abcg1 shRNA-2FW	5'-ccgg**CATGCCTACTGTTCTGACATT**ctcgag**AATGTCAGAACAGTAGGCATG**tttttg-3′targeting Abcg1 CDS mRNA 1380-1400 nt (V1)	Silencing of Abcg1
44	Abcg1 shRNA-2RV	5′-aattcaaaaa**CATGCCTACTGTTCTGACATT**ctcgag**AATGTCAGAACAGTAGGCATG**-3′	Silencing of Abcg1
45	Abcg1 shRNA-3FW	5'-ccgg**GACTTCATCGTACTCGGGATT**ctcgag**AATCCCGAGTACGATGAAGTC**tttttg-3′ targeting Abcg1 CDS mRNA 1921-1941 nt (V1)	Silencing of Abcg1
46	Abcg1 shRNA-3RV	5′-aattcaaaaa**GACTTCATCGTACTCGGGATT**ctcgag**AATCCCGAGTACGATGAAGTC**-3′	Silencing of Abcg1
47	Abcg1-Q-FW	5′-GTCTGAACTGCCCAACCTACCAC-3′	Abcg1 qRT-PCR
48	Abcg1-Q-RV	5′-CCGACTGTTCTGATCACCGTACTC-3′	Abcg1 qRT-PCR

49	β-actin-Q-FW	5′-ATTGCCGACAGGATGCAGA-3′	β-actin qRT-PCR
50	β-actin-Q-RV	5′-GAGTACTTGCGCTCAGGAGGA-3′	β-actin qRT-PCR
51	ECM1-SQ-PCR-FW	5′-TCACAGCATACTCCCGGTCACA-3′	ECM1 semi-RT-PCR
52	ECM1-SQ-PCR-RV	5′-TCTCAGCATGGCCCTCCCTTTGA-3′	ECM1 semi-RT-PCR
53	hnRNP L siRNA (h)	sc-38284, Santa Cruz Biotech, CA, USA	Silencing of hnRNPL
54	hnRNP M siRNA (h)	sc-38286, Santa Cruz Biotech, CA, USA	Silencing of hnRNPM
55	hnRNP U siRNA (h)	sc-38298, Santa Cruz Biotech, CA, USA	Silencing of hnRNPU
56	Control siRNA	sc-37007, Santa Cruz Biotech, CA, USA	
57	HNRNPL-F	5′-CAAAGCCTACACGCTTGAATGT-3′	HNRNPL qRT-PCR
58	HNRNPL-R	5′-TGTCCACTGAGATTGGGGTTT-3′	HNRNPL qRT-PCR
59	HNRNPM-F	5′-TGGTCCGAGCAGACATTCTTG-3′	HNRNPM qRT-PCR
60	HNRNPM-R	5′-TGACGTGCATTGGTCTATCAAA-3′	HNRNPM qRT-PCR
61	HNRNPU-F	5′-GATCTTGGCGTTGCCTTCAAA-3′	HNRNPU qRT-PCR
62	HNRNPU-R	5′-CTCTAACTCGATCCTCTAAGGGG-3′	HNRNPU qRT-PCR
63	SRSF1-F	5′-GGAAGACGCGGTGTATGGTC-3′	SRSF1 qRT-PCR
64	SRSF1-R	5′-CACCTGCTTCACGCATGTG-3′	SRSF1 qRT-PCR
65	SRSF6-F	5′-ACATAGGACGCCTGAGCTACA-3′	SRSF6 qRT-PCR
66	SRSF6-R	5′-GCCGTACCCATTTTTGAGGTCTA-3′	SRSF6 qPCR
67	hSRSF6 si-1 sense	5′-CGAACAAAUGAGGGUGUAAUUTT-3	Silencing of SRSF6
68	hSRSF6 si-1 antisense	5′-AAUUACACCCUCAUUUGUUCGTT-3′	Silencing of SRSF6
69	hSRSF6 si-2 sense	5′-GCCCACAAGGAACGAACAAAUTT-3′	Silencing of SRSF6
70	hSRSF6 si-2 antisense	5′-AUUUGUUCGUUCCUUGUGGGCTT-3′	Silencing of SRSF6
71	hSRSF6 si-3 sense	5′-GUCGGUGCAGUUGGCAAGAUUTT-3′	Silencing of SRSF6
72	hSRSF6 si-3 antisense	5′-AAUCUUGCCAACUGCACCGACTT-3′	Silencing of SRSF6
73	SRSF1-FW	5′-ACT***ggatcc***ATGTCGGGAGGTGGTGTGATTCG-3′ with *Bam* HI	SRSF1 cDNA cloning

74	SRSF1-RV	5'-GATCCTT***gcggccgc***TTAcagatcctcttcagagatgagtttctgctcTGTACGAGAGCGAGATCTGCTATG-3′ with *Not* I site and myc-tag	SRSF1 cDNA cloning
75	Abcg1-PF2	5'-gattc**GGATCC**atggcctgtctgatggccgctttct-3′	Abcg1 cloning in pEBG
76	Abcg1-RV2	5'-cttatc**GCGGCCGC**ttaCAGATCCTCTTCTGAGATGAGTTTTTGTTCcctctctgcccggattttgtacc-3’ with *Not* I site and myc tag	Abcg1 cloning in pEBG
77	Akt2-WT-PF	5'-gcgcg***GGATCCTCTAGA***atgaatgaggtgtctgtcatcaaag-3′ with *Bam*H I and *Xba* I sites	AKT2-WT cloning
78	Akt2-WT-RV	5'-cgatcag***CTCGAGGAATTC***tca**CTTATCGTCGTCATCCTTGTAATC**ctcgcggatgctggccgagtaggagaa-3′ with flag tag and *Xho I* and *Eco*R I sites	AKT2-WT cloning
79	Akt2-MT	5'-gcgcg***GGATCCTCTAGA***atgaatgaggtgtctgtcatcaaag-3′ with *Bam*H I and *Xba* I sites	AKT2-MT cloning
80	Akt2-MT	5'-cgatcag***CTCGAGGAATTC***tca**CTTATCGTCGTCATCCTTGTAATC**ctcgcggatgctggccgagtagg**C**gaa-3' with flag, *Xho* I and *Eco*R I sites, and a mutation	AKT2-MT cloning
81	PPP2CB-F	5'-ATTCAGTGGAGACTGTGACTCT-3′	PPP2CB qRT-PCR
82	PPP2CB-R	5'-GGCTTTCGTGATTTCCTCTCAAT-3′	PPP2CB qRT-PCR
83	PPP1R14C-F	5'-CTGGGTCAGCTCTACGGCT-3′	PPP1R14C qRT-PCR
84	PPP1R14C-R	5'-CATGCCTCTTATCCGAGAAAGC-3′	PPP1R14C qRT-PCR
85	GAPDH-SQ-PCR-FW	5′-AGGTCGGAGTCAACGGATTTG-3′	GAPDH semi-RT-PCR
86	GAPDH-SQ-PCR-RV	5′-GTGATGGCATGGACTGTGGT-3′	GAPDH semi-RT-PCR

Supplementary Table 7. Antibodies used in the study

Antibodies	Providers	Catalog Number	Clone name	Lot Number	Dilution	Mw
Co-IP* and WB						
Anti-ECM1	Santa Cruz Biotech, CA, USA	sc-365946	C-12	L8342	1:500	85
Anti-HA	Cell Signaling Technology, Massachusetts, USA	#2367	6E2	7	1:1000	
Anti-integrin β2	Santa Cruz Biotech, CA, USA	sc-8420	CTB104	B8463	1:500	95
Anti-integrin αX	Abcam, Cambridge, UK	Ab52632	EP1347Y	GR56392-33	1:1000	128
Anti-ABCG1	SAB, Maryland, USA	#37074		4926	1:1000	76
Anti-p-paxillin(Tyr118)	Cell Signaling Technology, Massachusetts, USA	#2541		09	1:1000	68
Anti-p-myosin IIa (Ser1943)	Cell Signaling Technology, Massachusetts, USA	#14611	D7Z7T	1	1:1000	230
Anti-AKT	Cell Signaling Technology, Massachusetts, USA	#2938	C73H10	2	1:1000	60
Anti-p-AKT (S473)	Cell Signaling Technology, Massachusetts, USA	#9018	D7F10	04	1:1000	60
Anti-FAK	Santa Cruz Biotech, CA, USA	sc-558	C-20	E2014	1:1000	125
Anti-p-FAK397	Cell Signaling Technology, Massachusetts, USA	#8556	D20B1	1	1:1000	125
Anti-paxillin	Cell Signaling Technology, Massachusetts, USA	#2542		6	1:1000	68
Anti-p-paxillin (Y118)	Cell Signaling Technology, Massachusetts, USA	#2541		1	1:1000	68
Anti-myosin IIa	Cell Signaling Technology, Massachusetts, USA	#3403		2	1:1000	230
Anti-Rho	Cell Signaling Technology, Massachusetts, USA	#9968		2	1:1000	21
Anti-p-RAC1 (S71)	Cell Signaling Technology, Massachusetts, USA	#9968		1	1:1000	28
Anti-hnRNPLL	Cell Signaling Technology, Massachusetts, USA	#4783		2	1:1000	61
Anti-c-myc	Cell Signaling Technology, Massachusetts, USA	#5605	D84C12	12	1:1000	57-65
Anti-Sox2	Cell Signaling Technology, Massachusetts, USA	#3579	D6D9	8	1:1000	35

Anti-Oct4A	Cell Signaling Technology, Massachusetts, USA	#2840	C30A3	15	1:1000	45
Anti-Intergrin β1	Proteintech, Hubei, China	12594-1-AP		00051218	1:1000	130
Anti-JUP	Proteintech, Hubei, China	27872-1-AP		00057630	1:1000	82
Anti-Annexin A2	Proteintech, Hubei, China	66035-1-Ig	1C1E12	00060800	1:1000	39
Anti-Calnexin	Proteintech, Hubei, China	10427-2-AP		00050122	1:1000	90
Anti-S100A9	Proteintech, Hubei, China	26992-1-AP		00050010	1:1000	14
Anti-Plakophilin-1	Abcam, Cambridge, UK	ab183512	EPR14890	GR153672-2	1:2000	75
Anti-S100A8	Proteintech, Hubei, China	15792-1-AP		00070065	1:1000	11
Anti-Fibrinogen alpha chain	Abcam, Cambridge, UK	ab92572	EPR2919	GR3323834-1	1:1000	95
Anti-Fibrinogen gamma chain	Abcam, Cambridge, UK	ab96532		GR28505-13	1:1000	50
Anti-ICAM1	Abcam, Cambridge, UK	ab109361	EPR4776	GR3356796-3	1:1000	89
Anti-Plasminogen	Abcam, Cambridge, UK	ab242329	EPR22254-115	GR3249656-4	1:1000	91
Anti-C3b / iC3b	Abcam, Cambridge, UK	ab231078	7C12	GR3258138-2	1:1000	115
Anti-CD90 / Thy1	Abcam, Cambridge, UK	ab92574	EPR3132	GR37919-38	1:500	18
Anti-PF4	Abcam, Cambridge, UK	ab129183	EPR7763	GR91410-16	1:500	11
Anti-HNRNPL	Absin Bioscience Inc. Shanghai, China	abs118485-50ul		DE02	1:1000	
Anti-HNRNPM	Absin Bioscience Inc. Shanghai, China	abs117522-50ul		DC02	1:1000	
Anti-HNRNPU	Absin Bioscience Inc. Shanghai, China	abs105213-50ul		NA29	1:1000	
Anti-SRSF1	Absin Bioscience Inc. Shanghai, China	abs115679-50ul		DA02	1:800	
Anti-SRSF6	Proteintech, Hubei, China	11772-1-AP		00002378	1:1200	55
Anti-PPP2CB	Abcam, Cambridge, UK	Ab168371	EPR11786(B)	GR124061-3	1:2000	36
Anti-PPP1R14C	Signalway Antibody LLC, MD. USA	33982-1		4813	1:2000	20
Anti-Flag	Proteintech, Hubei, China	66008-2-Ig	1E7B4	10002973	1:1000	
Anti-myc	Proteintech, Hubei, China	16286-1-AP		00053868	1:1000	
Anti-β-actin	Cell Signaling Technology, Massachusetts, USA	#4967		5	1:1000	45

Anti-rabbit IgGHRP-linked	Cell Signaling Technology, Massachusetts, USA	#7074		2	1:1000	
anti-mouse IgGHRP-linked	Cell Signaling Technology, Massachusetts, USA	#7076		1	1:1000	
IF staining						
Anti-ECM1	Santa Cruz Biotech, CA, USA	sc-365946	C-12	L8342	1:50	85
Anti-integrin αX	Abcam, Cambridge, UK	Ab52632	EP1347Y	GR56392-33	1:50	128
Anti-integrin β2	Santa Cruz Biotech, CA, USA	sc-8420	CTB104	B8463	1:50	95
Anti-HA	Cell Signaling Technology, Massachusetts, USA	#2367	6E2	7	1:50	
Anti-myosin IIa	Cell Signaling Technology, Massachusetts, USA	#3403		2	1:50	230
Anti-p-myosin IIa (S1943)	Cell Signaling Technology, Massachusetts, USA	#14611	D7Z7T	1	1:50	230
Anti-ABCG1	SAB, Maryland, USA	37074		4926	1:50	76
Alexa Fluor® 488 Donkey Anti–mouse IgG	Molecular Probes, Invitrogen, Carlsbad, CA, USA	A21202		2266877	1:1000	
Alexa Fluor® 488 Donkey Anti–rabbit IgG	Molecular Probes, Invitrogen, Carlsbad, CA, USA	A21206		2256732	1:1000	
Alexa Fluor® 555 Donkey Anti–mouse IgG	Molecular Probes, Invitrogen, Carlsbad, CA, USA	A31570		2273774	1:1000	
Alexa Fluor® 555 Donkey Anti–rabbit IgG	Molecular Probes, Invitrogen, Carlsbad, CA, USA	A31572		2286312	1:1000	
Alexa Fluor® 488 Donkey Anti–goat IgG	Molecular Probes, Invitrogen, Carlsbad, CA, USA	A11055		2211210	1:1000	
Neutralization						
Anti-ECM1	R&D Systems, Inc.Minneapolis, MN, USA	MAB3937	966248	PQD0218021	1:300	
Anti-CD11c (Integrin αX)	R&D Systems, Inc.Minneapolis, MN, USA	MAB17771	538207	CDMS0113101	1:300	
Anti-CD18 (Integrin β2)	R&D Systems, Inc.Minneapolis, MN, USA	AF1730		JZR0115031	1:2000	
IHC						

Anti-ECM1-01	Wuhan Pujian Biotechnology, Hubei, China				1:50	
Anti-ECM1-02	Wuhan Pujian Biotechnology, Hubei, China				1:50	
Anti-ECM1-03	Wuhan Pujian Biotechnology, Hubei, China				1:50	
Anti-integrin αX	Abcam, Cambridge, UK	Ab52632	EP1347Y		1:50	128
Anti-integrin β2	Santa Cruz Biotech, CA, USA	sc-8420	CTB104		1:50	95
Anti-ABCG1	SAB, Maryland, USA	37074		4926	1:50	76
Anti-hnRNPLL	Cell Signaling Technology, Massachusetts, USA	#4783		2	1:50	61
Goat Anti-Mouse IgG	Bioss antibodies,China	Bs-0296G-Bio		AG03086698	1:1000	
Biotinylated goat anti-rabbit IgG	Jackson ImmunoResearch Laboratories, PA, USA	111-065-144		147832	1:1000	
FCM						
Anti-CD117	BD Pharmingen，San Diego，USA	555714		7212837	1ug per million cells	
Anti-CD326	BioLegend，California，USA	369814		13222942	5ul per million cells	
Anti-CD24	BD Pharmingen，San Diego，USA	555427		7354615	1ug per million cells	
Anti-CD133	BD Pharmingen，San Diego，USA	566596		80559645	1ug per million cells	
Anti-IgG κ	BD Pharmingen，San Diego，USA	565571		9080944	1ug per million cells	

*Antibodies used for co-IP assays were 2μg per 500μg total proteins.
