# Supplementary material for: Extracellular matrix protein-1 secretory isoform promotes ovarian cancer through increasing alternative mRNA splicing and stemness
Source: Nat Commun. 2021 Jul 9;12:4230. doi: 10.1038/s41467-021-24315-1 (PMC8270969; doi:10.1038/s41467-021-24315-1)
Supplement: Supplementary file 3 — Description of Additional Supplementary Files [file 41467_2021_24315_MOESM3_ESM.pdf]

Description of Additional Supplementary Files

Supplementary Data 1 KEGG analysis of altered signaling pathways between A8 and A8i cells based on RNA sequencing

Supplementary Data 2 KEGG analysis of altered signaling pathways between A8i-A and A8i cells based on RNA sequencing

Supplementary Data 3 KEGG analysis of altered signaling pathways between A8i-A and A8i-B cells based on RNA sequencing

Supplementary Data 4 Potential proteins binding to intracellular ECM1a identified by mass-spectrum

Supplementary Data 5 Potential proteins binding to intracellular ECM1b identified by mass-spectrum
