# Supplementary material for: Extracellular matrix protein-1 secretory isoform promotes ovarian cancer through increasing alternative mRNA splicing and stemness
Source: Nat Commun. 2021 Jul 9;12:4230. doi: 10.1038/s41467-021-24315-1 (PMC8270969; doi:10.1038/s41467-021-24315-1)
Supplement: Supplementary file 9 — Reporting Summary [file 41467_2021_24315_MOESM9_ESM.pdf]

nature research

Corresponding author(s): Gong Yang, yanggong@fudan.edu.cn
Chunxia Ren, renchunxia2018@shutcm.edu.cn

Last updated by author(s): 2021-May-31

Reporting Summary

Nature Research wishes to improve the reproducibility of the work that we publish. This form provides structure for consistency and transparency in reporting. For further information on Nature Research policies, see our Editorial Policies and the Editorial Policy Checklist.

Statistics

For all statistical analyses, confirm that the following items are present in the figure legend, table legend, main text, or Methods section.

n/a	Confirmed	
☐	☒	The exact sample size (n) for each experimental group/condition, given as a discrete number and unit of measurement
☐	☒	A statement on whether measurements were taken from distinct samples or whether the same sample was measured repeatedly
☐	☒	The statistical test(s) used AND whether they are one- or two-sided<br>*Only common tests should be described solely by name; describe more complex techniques in the Methods section.*
☐	☒	A description of all covariates tested
☐	☒	A description of any assumptions or corrections, such as tests of normality and adjustment for multiple comparisons
☐	☒	A full description of the statistical parameters including central tendency (e.g. means) or other basic estimates (e.g. regression coefficient) AND variation (e.g. standard deviation) or associated estimates of uncertainty (e.g. confidence intervals)
☐	☒	For null hypothesis testing, the test statistic (e.g. F, t, r) with confidence intervals, effect sizes, degrees of freedom and P value noted<br>*Give P values as exact values whenever suitable.*
☒	☐	For Bayesian analysis, information on the choice of priors and Markov chain Monte Carlo settings
☐	☒	For hierarchical and complex designs, identification of the appropriate level for tests and full reporting of outcomes
☐	☒	Estimates of effect sizes (e.g. Cohen's d, Pearson's r), indicating how they were calculated

Our web collection on statistics for biologists contains articles on many of the points above.

Software and code

Policy information about availability of computer code

Data collection	No software was used
Data analysis	No software was used

For manuscripts utilizing custom algorithms or software that are central to the research but not yet described in published literature, software must be made available to editors and reviewers. We strongly encourage code deposition in a community repository (e.g. GitHub). See the Nature Research guidelines for submitting code & software for further information.

Data

Policy information about availability of data

All manuscripts must include a data availability statement. This statement should provide the following information, where applicable:
- Accession codes, unique identifiers, or web links for publicly available datasets
- A list of figures that have associated raw data
- A description of any restrictions on data availability

The accession codes are provided in "Data availability statement", the unique identifiers or web links are provided for publicly available datasets. The list of figures that have associated raw data is provided. The description of restrictions on data availability is provided.

Field-specific reporting

Life sciences study design

All studies must disclose on these points even when the disclosure is negative.

Sample size	For animal assays, a pilot study indicated that at least 5 mice for each group of tested cell lines were required to detect differences in tumor size with 80% power at a P value of less than 0.05. For IHC staining of each molecules and Kaplan-Meier survival analysis, at least 50 tissues from different cases were required in the preanalysis with 80% power at a P value of less than 0.05.
Data exclusions	The data from unexpectedly dead animals were excluded. The data from in vitro assays obtained from cells contaminated with mycoplasma or bacteria were excluded. Any data suspected with artificiality during experiments were excluded.
Replication	All experiments were repeated at least three times. Each test for some assays such as qRT-PCR was performed in triplicate. All data are reproducible.
Randomization	Animals used in this study were randomized for each group assay.
Blinding	For immunohistochemical staining of human ovarian cancer tissue array (TMA), the staining of tissues, intensity of tissues, and cases were blinded for pathologists and researchers. All statistical analyses were performed blindly by statisticians.

Reporting for specific materials, systems and methods

We require information from authors about some types of materials, experimental systems and methods used in many studies. Here, indicate whether each material, system or method listed is relevant to your study. If you are not sure if a list item applies to your research, read the appropriate section before selecting a response.

Materials & experimental systems

n/a	Involved in the study	
☐	☒	Antibodies
☐	☒	Eukaryotic cell lines
☒	☐	Palaeontology and archaeology
☐	☒	Animals and other organisms
☐	☒	Human research participants
☐	☒	Clinical data
☒	☐	Dual use research of concern

Methods

n/a	Involved in the study	
☒	☐	ChIP-seq
☐	☒	Flow cytometry
☒	☐	MRI-based neuroimaging

Antibodies

Antibodies used	All antibodies used are listed in Supplementary Table 12 containing the detailed information. Three custom antibodies were generated by a company described in the manuscript.
Validation	All antibodies used in this study are validated or pretested with detection stability by the researchers. The results were included in the manuscript. The data sheets of all antibodies are available in each company's website.

Eukaryotic cell lines

Policy information about cell lines

Cell line source(s)	Human ovarian epithelial cancer cell lines SKOV3 was from the American Tissue Culture Collection (ATCC); human ovarian epithelial cancer cell lines HEY, HEYA8, SKOV3ip1, OVCA429, OVCA433, ES-2 and A2780 were from Dr. Bast, RC Jr of University of Texas, MD Anderson Cancer Center, Houston, Texas (USA). The immortalized human ovarian surface epithelial cell line T29 was from Dr. Liu, Jingsong of University of Texas, MD Anderson Cancer Center, and the normal HOSE cell line and the immortalized fallopian tube epithelial cell line (FTE) were established and reported previously in our lab.
Authentication	SKOV3, HEY, OVCA429, OVCA433, ES-2 and A2780 cell lines were authenticated by the third party -ATCC or GENETIC TESTING BIOTECHNOLOGY Co. Ltd, in Suzhou, China according to the STR data in the public database. HEYA8 and SKOV3ip1 cell lines were derived from HEY and SKOV3 cell lines, respectively, so they are isogenic cell lines. However, HEYA8 and SKOV3ip1 do not have STR data in public database, so we had these two cell lines authenticated by comparison of their STR data with those of HEY and SKOV3 cell lines, respectively. The authentication documents are available.
Mycoplasma contamination	Mycoplasma contamination of each cell line was routinely tested before each experiment was conducted.
Commonly misidentified lines (See ICLAC register)	None

Animals and other organisms

Policy information about studies involving animals; ARRIVE guidelines recommended for reporting animal research

Laboratory animals	4- to 6-week-old BALB/c athymic nude female mice from Shanghai SLAC Laboratory Animal Co., Ltd were maintained in a pathogen free environment
Wild animals	None
Field-collected samples	Upon the end of the experiments, mice were sacrificed by CO2 inhalation, and tumor tissues were isolated and weighted. No field collected samples were made during experiments.
Ethics oversight	The mouse experiments were approved by the Institutional Animal Care and Use Committee of Fudan University Shanghai Cancer Center and performed following the institutional guidelines and protocols.

Note that full information on the approval of the study protocol must also be provided in the manuscript.

Human research participants

Policy information about studies involving human research participants

Population characteristics	Ovarian or fallopian tissue samples from patients, who were diagnosed with primary high-grade epithelial ovarian cancer or fallopian tube diseases (from whom normal fallopian or ovarian tissues were also collected, respectively) and had undergone initial surgery at Fudan University Shanghai Cancer Center between June 2013 and December 2016, were selected in this study. A total of 150 cumulative patients were identified with updated follow-up information until January 20th, 2017. Histopathologic diagnoses were based on the World Health Organization (WHO) criteria; tumor grades were based on the Gynecologic Oncology Group criteria. The TMA consisted of core samples from 30 normal human ovarian tissues and 150 human OCs were used to built a tissue microarray (TMA) for analysis of the marker expressions and their association with patient pathological characteristics.
Recruitment	This study was retrospective, specimens were selected from patients with primary high-grade epithelial ovarian cancer.
Ethics oversight	All patients signed an informed consent form showing that their blood or tissues might be used for various research purposes before or after diagnosis and treatments. The study was approved by the Ethics Committee of Fudan University Shanghai Cancer Center.

Note that full information on the approval of the study protocol must also be provided in the manuscript.

Clinical data

Policy information about clinical studies

All manuscripts should comply with the ICMJEguidelines for publication of clinical research and a completedCONSORT checklist must be included with all submissions.

Clinical trial registration	None
Study protocol	This is a retrospective study, the study protocol includes patients with primary high-grade epithelial ovarian cancer or fallopian tube diseases, and histopathologic diagnoses based on the World Health Organization (WHO) criteria; tumor grades were based on the Gynecologic Oncology Group criteria.
Data collection	The staining for each protein was scored by immunostaining intensity and tumor cell percentages. The data were collected by two pathologists based on double blind scoring.
Outcomes	Patient characteristics including the disease records and survival status were either from the original archives or from the updated follow-up information after treatment. The clinical outcomes (survival) were calculated by the Kaplan-Meier method and compared by the log-rank test in terms of protein expression and survival months.

Flow Cytometry

Plots

Confirm that:

- [x] The axis labels state the marker and fluorochrome used (e.g. CD4-FITC).
- [x] The axis scales are clearly visible. Include numbers along axes only for bottom left plot of group (a 'group' is an analysis of identical markers).
- [x] All plots are contour plots with outliers or pseudocolor plots.
- [x] A numerical value for number of cells or percentage (with statistics) is provided.

Methodology

Sample preparation	A single-cell suspension with approximately 1×10^6 cells was prepared for staining with antibodies against CD117 (555714, BD Pharmingen), CD24 (555427, BD Pharmingen), CD326 (369814, BioLegend), and CD133 (566596, BD Pharmingen) or a control antibody (IgG κ isotype control). The cells were stained at 4°C for 30 min in the dark, washed with PBS, resuspended in 100 µl of PBS after centrifugation, and finally subjected to flow cytometry analysis or selection.
Instrument	The flow cytometers used for analysis and selection were Beckman Cytomics FC 500 MPL and MOFLO XDP, respectively (Beckman Coulter, USA).
Software	Summit 5.2 was used for analysis, while MXP was used for selection.
Cell population abundance	Cell population abundance was determined by comparing the stained cells with negative controls. Specific dye-conjugated antibodies used to stain the cell population was gated by specified laser channels recognizing PE and FITC
Gating strategy	The cells were first gated on a forward scatter (FS)/side scatter (SS) plot (a) and then gated on specific populations. The gating strategy is provided in Supplementary Figure 15 of the Supplementary Information.

☒ Tick this box to confirm that a figure exemplifying the gating strategy is provided in the Supplementary Information.
