# Supplementary material for: Extracellular matrix protein-1 secretory isoform promotes ovarian cancer through increasing alternative mRNA splicing and stemness
Source: Nat Commun. 2021 Jul 9;12:4230. doi: 10.1038/s41467-021-24315-1 (PMC8270969; doi:10.1038/s41467-021-24315-1)
Supplement: Supplementary file 2 — Peer Review File [file 41467_2021_24315_MOESM2_ESM.pdf]

Editorial Note: Parts of this Peer Review File have been redacted as indicated to maintain the confidentiality of unpublished data.

Reviewers' comments:

Reviewer #1 (Remarks to the Author); expert in ovarian cancer and mouse models:

In the present manuscript, the authors report the different roles and mechanisms of ECM1 splice variants in tumorigenesis. In particular, ECAM1a, which mediates integrin aXb2-mediated cytoskeleton signaling, hnRNPLL-mediated mRNA alternative splicing, and ABCG1-induced cancer cell stemness. Therefore, targeting ECM1a signaling may provide optimal therapy.
1. It is unclear what is the rationale of using ovarian cancer as a model. Is ECM1 particularly relevant to its pathogenesis?
2. Does ECM1a have same abilities to regulate cytoskeleton signaling, alternative splicing and stemness in non-serous ovarian cancer or other cancer types?
3. As serous ovarian cancer probably also may arise from the fallopian tube, please include it as a control.
4. Figure 1A: it is important to show the expression of the different ECM1 splice variants among the cell lines, for example, which one is the most abundant and which is secretory? It is difficult to conclude from Figure 2C and D which one is cytoplasmic. The authors shall then dissect the function of the secretory versus the non-secretory ECM1.
5. Figure 1C, the silencing of specific transcripts should be shown. Figure 1E, please indicate which shRNA is used.
6. The finding on a possible novel function of ABCG1 in phosphorylating downstream signaling molecules is interesting. The authors should provide more evidence on the direct phosphorylation. What is the probable significance for ECM1 to mediate cytoskeletal signaling via ABCG1?

Reviewer #2 (Remarks to the Author); expert in ECM and cytoskeleton:

This manuscript attempts to demonstrate how hnRNPLL mediates differential splicing of Extracellular Matrix Protein-1 (ECM1), binding to integrins, and ultimately signaling through ABCG1 to contractile proteins or to promote drug resistance via CD326-mediated stem-like behaviors. Authors posit that this mechanism could be useful therapeutically. Overall I found this to be a compelling story (especially later figures with drugs) but some of the data requires some clarification as noted below.

1. The RNAseq data section starting on line 112 is confusing in how it's written. The authors do not clearly identify which samples they are comparing at the outset, and once the comparison is made, Figure S3 doesn't indicate relative to which sample up/down-regulation occurs. More importantly, the presentation of GO and KEGG data in Figure S3 is not helpful as common outcomes are hard to see. Also the conclusion that Fig. S4A, C, F shows that integrins, ABC transporters, and HNRNP were highly altered by ECM1 silencing and ECM1a overexpression is not clear, at least in panels A and F. There isn't any pattern in the heat maps, so the authors should delete this or show it differently (more quantitatively).

2. The co-IP information in Figure 2 is intriguing but it only shows that they bind, not specifically where. Given how little is known about ECM1, it would be nice to know if this competitively inhibits matrix protein binding, i.e. does it cover the ECM-binding site.

3. Is dimerization of alphaX with beta2 integrin required for any of these effects. To put it another way, is a functioning integrin required or not?? IF alone of each monomer doesn't demonstrate this, just that they are localized appropriately.

4. Spectra in Figure 3C are not helpful as they are both difficult to see and interpret. Rather some sort of normalized spectral abundance factor should be provided to describe quantification.

Minor:
1. In the title, the authors say "Ruling of..." but I believe that they mean "Regulation of..." I think that the latter is more grammatically and scientifically correct.

2. In Figure 1L, I do not agree with the author's statement that FAK397 is attenuated with inhibition, i.e. A8i and A8i-V. Overexpression is a bit more convincing, but not the reduction. I suggest eliminating this comment.
3. On line 128, the authors state: "...we further tested by immunofluorescence..." What did you test? I believe that the sentence should say "...we further tested integrin αXβ2 expression and localization, finding that it was down-regulated when ECM1 was silenced or ECM1b and ECM1c overexpressed but up-regulated in ECM1a overexpressing cells..."
4. Cisplatin axises in Figure 6 appear to show the wrong scale of concentration (mm)???

Reviewer #3 (Remarks to the Author); expert in ECM and cytoskeleton:

In this manuscript, the authors identify a specific splicing inform of ECM1 as one that promotes tumorigenesis. They go onto show its interaction with a specific integrin and its role in activating and attenuating specific oncogenes. And lastly, they show the presence of ECM-1 correlates with prognosis. The paper is very through in that it explore a numbers of different avenues. However, it is somewhat disjointed for the same reason. At points, it is difficult to understand the transition from experiment to experiment or how the results integrate together. More specific comments below:

Abstract does not connect well to itself and is difficult to read. The authors need to better connect the ideas and pathways they are pursuing.

Are the results specific to ovarian cancer? This is an important issue to address either experimentally, or to clarify the abstract, title and text by making it more clear this study focuses on ovarian exclusively.

Many cell lines were tested in Figure 1A, and then only A8 cells were used in the other part of Figure 1. Why?

The inconsistency between the text labels in the figures and the main text make it difficult to read.

Figure 1M- is it is not clear why treatment with the anti-HA antibody would prevent secretion.

Did expression of the mutant ECM1 decrease expression of FAK, AKT etc, or did it decrease the phosphorylation (activation) of these signaling molecules?

Line 159 may be referencing the wrong figure?

There is no quantification of western blots

How is ECM1 leading to phosphorylation of numerous cancer genes?

The authors open the manuscript but pointing out the inconsistencies in the literature on ECM-1. It is not clear that they have clarified the literature.

Reviewer #4 (Remarks to the Author); expert in mRNA alternative splicing:

This is generally a very thorough and well performed study on the role of the ECM1 isoforms 1a, 1b and 1c and their potentially different roles in tumorigenesis. Several previous studies have documented a role for ECM1 in tumorigenesis and ECM1 has been implicated in several regulatory pathways, as for instance TGFB signaling. Knowledge about the potential different roles of the different ECM1 isoforms is however lacking.
The authors initially demonstrate that ECM1 (without distinguishing between the isoforms) is over-expressed in cancers. This is consistent with all previous studies indicating that ECM1 is involved in tumorigenesis.
Using siRNA and overexpression studies the authors convincingly demonstrate that it is in

particular the ECM1a isoform which is a tumorigenic driver and that this involves interaction with integrin αXβ2.
One of the major claims in this paper is that the different ECM1 isoforms have different roles in tumorigenesis. Generally, this claim is well supported by the data, but I am a bit worried by the fact that much is based on overexpression (combined with KD) of the three isoforms to point at ECM1a as the tumorigenic isoform. Overexpression of the individual isoforms may result in artificially high protein levels and therefore do not mimic the real situation. In fact, there is some effect of overexpressing the ECM1b isoform in KD cells (both on tumor size and AKT activation etc.) - Check fig 2 supplemental.
It would be beneficial if the authors had demonstrated the effect of overexpression (in particular the ECM1a and ECM1b isoforms) in cells (and mice?) without shRNA treatment.
I also worry about the expression levels of overexpressed ECM1a, ECM1b and ECM1c. In figure 1D it appears that ECM1a is significantly (2fold? or more?) higher overexpressed than ECM1b.
This could have implications for this and subsequent experiments – This tendency of more ECM1a is also apparent in the co-ip experiments. Therefore the authors need to control better that the level of overexpression is comparable between the three isoforms.
The authors should demonstrate by shRNA KD of the individual isoforms that ECM1a and not ECM1b and ECM1c is tumorigenic. This should be feasible at least for 1a and 1b isoforms given the fact that exon 7 distinguishes these isoforms.
Abolishing one isoform at a time by shRNA KD (decreasing the endogenous level) may better reveal their functional difference, since the remaining isoforms will have more realistic protein levels.
Apart from the potentially questionable comparability between the levels of expression between the three isoforms the experiments very clearly establishes a role for ECM1a in tumorigenesis and that this involves the Akt/Fak/Paxillin/Rho-associated signaling axis in ovarian cancer. The lack of tumorigenicity of ECM1b and ECM1c seems plausible, but is potentially questionable by lower levels of overexpression of in particular ECM1b.

The authors then use RNA-seq analysis to further detect and delineate the involved pathways. This points them towards integrins αX and β2, ABCG1, and HNRNPLL, which they demonstrate (fig 4) to be dependent on the presence of ECM1a, as knock down leads to a dramatically lower expression that can not be rescued by the ECM1b and ECM1c isoforms (but again: was ECM1b and ECM1c overexpressed at the same level as ECM1a?).
Using HA-tagged proteins and IP they demonstrate that ECM1b and ECM1c interact with non-phosphorylated myosin. This together with other experiments indicate that phosphorylation of myosin is essential for the signaling induced by the ECM1a–integrin αXβ2 interaction.
Next they move on to demonstrate that a positive feedback exist between ECM1a and hnRNPLL, where they both stimulate each others expression. This could have been detailed a bit more, as it is tempting to speculate that hnRNPLL is directly involved in regulating ECM1 splicing, whereas ECM1a stimulation of hnRNPLL expression is likely indirect. It would have been interesting also to know if the expression of other splicing factors than hnRNPLL are changed. The data are, however, consistent with previous studies showing that hnRNPLL expression also promotes colorectal cancer cell proliferation, but it can not be concluded if the observed effect of hnRNPLL is directly on ECM1 splicing or perhaps on mRNA stability.
Next the authors the authors demonstrate that ABCG1 could mediate the ECM1a-integrin αXβ2 interactive signaling to control tumorigenesis through the regulation of AKT/FAK/Paxillin/Rac/myosin protein phosphorylation and also that ABCG1 overexpression may mediate stemness through upregulation of cd326.
Finally the authors analyze patient material and demonstrates that the identified proteins may be used as prognostic markers for patients with ovarian cancer.

In conclusion the authors very convincingly demonstrate the role of ECM1a isoform in (ovarian) cancer and they delineate some of the pathways/factors involved. This is new and important knowledge. They also, a little less convincingly show that ECM1b and ECM1c isoforms do not function in same way as ECM1a and that they may even have opposing roles through interaction with different protein partners (for instance phosphorylate Myosin).

Minor points:

Generally, I am missing a little more background information about the three isoforms. Which functional domains are affected etc.

The claim that hnRNPLL may be targeted for optimal therapy in cancers associated with the ECM1 signaling is a not well documented. hnRNPLL regulates thousands of alternative splicing events, so not only could there be serious side effects from targeting hnRNPLL, but there could potentially be oncogenic effects since most likely hnRNPLL also regulates other protooncogenes and tumor suppressor genes in the opposite direction.

RESPONSE TO REFEREES

Dear Referees,

We sincerely appreciate your helpful review and the critical comments. Herein, we have carefully addressed all the issues that you concerned.

Reviewer #1 (Remarks to the Author); expert in ovarian cancer and mouse models:

In the present manuscript, the authors report the different roles and mechanisms of ECM1 splice variants in tumorigenesis. In particular, ECAM1a, which mediates integrin aXb2-mediated cytoskeleton signaling, hnRNPLL-mediated mRNA alternative splicing, and ABCG1-induced cancer cell stemness. Therefore, targeting ECM1a signaling may provide optimal therapy.

Q1. It is unclear what is the rationale of using ovarian cancer as a model. Is ECM1 particularly relevant to its pathogenesis?

A1. Many thanks. We selected ovarian cancer (OC) as a model because OC lacks early diagnosis, but is featured with high metastasis, chemoresistance, and poor survival. Because ECM1 was reported highly secreted in OC patient serum by Kristin et al. in 2010[1], we first investigated the function of ECM1 in OC, but now we have extended this research in different cancer types including breast cancer, lung cancer, liver cancer, etc.

Q2. Does ECM1a have same abilities to regulate cytoskeleton signaling, alternative splicing and stemness in non-serous ovarian cancer or other cancer types?

A2. Good questions! The preliminary data tested from ES-2 cells (a human ovarian clear cell adenocarcinoma cell line), and MDA-MB-231 cells (a human breast cancer cell line) show that ECM1a up-regulates cytoskeleton signaling in both cell lines (SFig. 3A and the rebuttal letter Figure 1 [rFigure 1] at the end of this letter). Both hnRNPLL

and ABCG1 were also up-regulated by ECM1a in ES-2 cells (SFig. 8C & SFig. 13J), but only ABCG1 was up-regulated by ECM1a in MDA-MB-231 cells (rFigure 1). In addition, overexpression of hnRNPLL in ES-2 cells promoted ECM1a alternative splicing (SFig. 8D-F), whereas overexpression of ABCG1 in ES-2 cells enhanced the expression of c-myc, SOX2 and Oct4A, and CD326+ cell population (4.2% vs 0.1%), (SFig.13K-L), indicating the increased cell stemness of ES-2 cells by ABCG1. The detailed study in breast cancer cells is being conducted by different researchers in the lab. The data will be separately published in near future. Therefore, we only included the data from ES-2 cells in the revised manuscript.

Q3. As serous ovarian cancer probably also may arise from the fallopian tube, please include it as a control.

A3. Good question! We selected an immortalized fallopian tube epithelial cell line that we previously established by introduction of hTERT and p53/pRb shRNAs[2]. This cell line appears with low ECM1a expression at both mRNA and protein levels, and with undetectable ECM1b and ECM1c expression at both mRNA and protein levels (SFig.1 A-C), whereas the tissue of normal fallopian tube epithelium shows low expression of ECM1 staining (Fig.1C). Introduction of ECM1a in FTE cells highly activated pAKT (S473), pFAK (Y397), pPaxillin (Y118), and pRac (S71), and induced tumor growth, compared with in cells transfected with ECM1b or vector (SFig.3B-D).

4. Figure 1A: it is important to show the expression of the different ECM1 splice variants among the cell lines, for example, which one is the most abundant and which is secretory? It is difficult to conclude from Figure 2C and D which one is cytoplasmic. The authors shall then dissect the function of the secretory versus the non-secretory ECM1.

A4. Many thanks. Among the tested cell lines, the expression of ECM1 splicing variants at mRNA and protein levels is shown in SFig.1A-C. ECM1a is the most abundant and secretory, and that ECM1b and ECM1c are much less abundant (SFig.1A-B), and are undetectable in both cell lysates and cultured media (SFig.1C). ECM1a is detected to

be secretory and interacting with integrin $\alpha X\beta 2$ in HEYA8 (A8) cells (SFig. 6A-B). Thus, we mainly investigated the biological function of ECM1a. However, we identified that non-secretory ECM1b interacts with myosin 9 within cytoplasma to inhibit the phosphorylation of myosin 9 at Ser1943 in Hey A8 cells (Fig. 4B-D).

Q5. Figure 1C, the silencing of specific transcripts should be shown. Figure 1E, please indicate which shRNA is used.

A5. Thanks a lot! Three shRNAs were all designed to target ECM1a, ECM1b, and ECM1c (Fig. 1E). Based on the silencing efficiency, we selected the cell line expressing both shRNA1 and shRNA2 (Fig.1 E and SFig. 1G-I).

Q6. The finding on a possible novel function of ABCG1 in phosphorylating downstream signaling molecules is interesting. The authors should provide more evidence on the direct phosphorylation. What is the probable significance for ECM1 to mediate cytoskeletal signaling via ABCG1?

A6. Great comments! To address this comment, we selected AKT2 as a substrate to perform the phosphorylation assay by ABCG1. The preliminary data showed that AKT2 might be phosphorylated at Ser473 by ABCG1 exogenously expressed in and purified from eukaryotic cells (HEK293T) (SFig. 11A-B). However, whether and how ABCG1, as a potential serine/threonine kinase, directly phosphorylates other molecules such as Rac and Myosin will require more investigations, whereas the full identification of ABCG1 as a kinase will also need more experiments. On the other hand, based on the RNA-seq data, we found that the serine/threonine protein phosphatase 2A catalytic subunit beta (PPP2CB) and the serine/threonine protein phosphatase 1 regulatory inhibitor subunit 14C (PP1R14C) are down-regulated and up-regulated, respectively, in parental and ECM1a overexpressing cells, but are reversely regulated in ECM1 silencing and ECM1b overexpressing cells (SFig. 11C-E). Furthermore, treatment of HeyA8i cells with okadaic acid, a specific inhibitor of serine/threonine protein phosphatases 1 and 2A, stimulated the phosphorylation of AKT/Rac/Myosin, whereas

management of HeyA8i-A (ECM1a overexpression) cells with ceramide, an activator of serine/threonine protein phosphatases 1 and 2A, blocked the phosphorylation of AKT/Rac/Myosin proteins (SFig.11F). These results suggest the phosphorylation of AKT/Rac/Myosin is regulated by ABCG1, and protein phosphatases or their inhibitors, whereas the tyrosine phosphorylation regulation of FAK and Paxillin in the ECM1-associated signaling requires additional investigations.

The probable significance for ECM1 to mediate cytoskeletal signaling via ABCG1 is that the activated signaling transduction triggered by binding of ECM1a to integrin $\alpha X\beta 2$ largely depends on the ABCG1-mediated phosphorylation of cytoskeletal molecules, which may mainly regulate the drug or cholesterol efflux to confer chemoresistance. Please also refer A9 to Reviewer 3.

Reviewer #2 (Remarks to the Author); expert in ECM and cytoskeleton:

This manuscript attempts to demonstrate how hnRNPLL mediates differential splicing of Extracellular Matrix Protein-1 (ECM1), binding to integrins, and ultimately signaling through ABCG1 to contractile proteins or to promote drug resistance via CD326-mediated stem-like behaviors. Authors posit that this mechanism could be useful therapeutically. Overall I found this to be a compelling story (especially later figures with drugs) but some of the data requires some clarification as noted below.

Q1. The RNAseq data section starting on line 112 is confusing in how it's written. The authors do not clearly identify which samples they are comparing at the outset, and once the comparison is made, Figure S3 doesn't indicate relative to which sample up/down-regulation occurs. More importantly, the presentation of GO and KEGG data in Figure S3 is not helpful as common outcomes are hard to see. Also the conclusion that Fig. S4A, C, F shows that integrins, ABC transporters, and HNRNP were highly altered by ECM1 silencing and ECM1a overexpression is not clear, at least in panels A and F. There isn't any pattern in the heat maps, so the authors should delete this or show it differently (more quantitatively).

A1. Many thanks. The description of the RNAseq data has been revised. The up/down-

regulated genes are indicated between cell lines of A8 and A8i, A8i-A and A8i, or A8i-A and A8i-B in SFig. 4A, whereas the alteration of genes between the paired cell lines analyzed by GO and KEGG are shown between the same paired cell lines in SFig. 4B and C. We have indicated the potentially altered pathways we are interested in with arrows in the GO and KEGGE diagrams between the paired cell lines, and listed the significant pathways analyzed by KEGG in STables 1-3. We also added more descriptions in the figure legend. Please see the revised SFigure 4, the figure legend, and Stables 1-3.

For the previous SFigure 4A, C, and F, we previously built the heatmaps by alphabetic sequence of genes. Now, in SFigure 5, we rebuilt the heatmaps by listing the genes of A8 cells from down-regulation to up-regulation. We can see that the expression of most integrins, hnRNPs, and ABCs is highly altered, and that at least half of the genes are significantly altered in A8i and A8i-B cells in comparison with those in A8 or A8i-A cells. Although some data are inconsistent between the RNA-seq data and qRT-PCR, the up-regulation of hnRNPLL, ABCG1, and integrins αX and β2 are strongly supported by western blot (WB), please see the revised SFig. 5B, D, F; Fig. 2A, B and D; Fig. 5B; and Fig. 6A.

Q2. The co-IP information in Figure 2 is intriguing but it only shows that they bind, not specifically where. Given how little is known about ECM1, it would be nice to know if this competitively inhibits matrix protein binding, i.e. does it cover the ECM-binding site.

A2. Great questions! The binding of ECM1a (not ECM1b) to integrin αXβ2 is mediated through the GPR motif of ECM1a because the GPR-to-VAQ mutation lost the binding activity of ECM1a to integrin αXβ2, which are shown in Figure 3H-K. However, it seems that the mutant ECM1 (ECM1-MT) could still bind to the plasma membrane (Figure 3H, right panel A8i-A-MT). We presumed that ECM1-MT could bind to additional plasma membrane components other than integrin αXβ2. To validate this hypothesis and to response the reviewer's comment, we prepared three batches of samples by co-IP using HA-tag antibody to pull down the cell surf ace

molecules potentially binding to ECM1a-WT and ECM1a-MT, respectively. Particular protein bands altered between ECM1a-WT and ECM1a-MT samples were analyzed by mass-spectrum (Fig. 6C-E). The results showed that keratins are the most abundant proteins equally binding to both ECM1-WT and ECM1-MT (STable 4), whereas talin, desmoplakin, junction plakoglobin (JUP), filaggrin-2, CD71, calnexin, annexin A1/A2, hornerin, desmoglein-1, plakophilin-1, S100-A7/A8/A9/A11, integrin β1 (CD29), and moesin differentially bound to either proteins (STable 4). By co-IP and western blots, we identified that integrin β1, JUP, Annexin A1, Calnexin, and S100A9 dominantly bind to ECM1-WT, whereas Plakophilin-1 and S100A8 mainly bind to ECM1-MT (SFig. 6F-L). Therefore, the binding of ECM1a-WT with integrin αX/β2 does not inhibit the binding to integrin β1, JUP, Annexin A1, Calnexin, and S100A9, but inhibits the binding to Plakophilin-1 and S100A8, suggesting that the binding between ECM1a and integrin αXβ2 does not cover the whole binding sites to other cell surface molecules, but does competitively inhibit the binding to some other membrane proteins such as Plakophilin-1 and S100A8. □

Q3. Is dimerization of alphaX with beta2 integrin required for any of these effects. To put it another way, is a functioning integrin required or not?? IF alone of each monomer doesn't demonstrate this, just that they are localized appropriately.

A3. Good questions. From the data shown in Fig. 2C-E, ECM1a binds to either αX or β2. Although the monomer integrin αX is supposed to function by binding only to integtrin β2, silencing of either or both of them in cells diminished the ECM1-mediated *in vitro* 3D and *in vivo* tumor growth (Fig. 2F-I), as well as the activation of cytoskeleton signaling (Fig. 2J, left panel). Our data suggest that the balanced expression for dimerization of αX and β2 is functionally required for ECM1-mediated signaling transduction and biological functions. However, to further validate this conclusion, we treated cells overexpressing ECM1a with antibodies to either or both integrin αX and integrin β2. We found that the phosphorylation of the involved cytoskeleton molecules was inhibited similarly to that by silencing of integrin αX/β2

(Fig. 2J, right panel).

Q4. Spectra in Figure 3C are not helpful as they are both difficult to see and interpret. Rather some sort of normalized spectral abundance factor should be provided to describe quantification.

A4. Thanks a lot! The data of spectra in former Fig. 3C are basepeak maps of sample A1 from A8i-A (ECM1a) and sample B1 from A8i-B (ECM1b). Now, we extracted the maps of secondary ion mass spectrometry for the most abundant protein listed from samples A1 and B1. It is clear that human myosin heavy polypeptide 9 was enriched in samples B1 (B1-6333), but not in A1, in which a human uncharacterized protein (A1-6959) was enriched in sample A1 (Fig. 4C). The list of identified peptides in each sample is shown in STables 5-6. The samples analyzed by LCMSMS in this study are selected from gel staining of equally co-immunoprecipitated products, which does not contain a normalized spectral abundance factor for description of quantification.

Minor:

q1. In the title, the authors say "Ruling of..." but I believe that they mean "Regulation of..." I think that the latter is more grammatically and scientifically correct.

a1. Thanks. We have revised the title accordingly.

q2. In Figure 1L, I do not agree with the author's statement that FAK397 is attenuated with inhibition, i.e. A8i and A8i-V. Overexpression is a bit more convincing, but not the reduction. I suggest eliminating this comment.

a2. Thanks. The detection of FAK Y397 in previous Fig. 1L was unstable. We repeated the experiment and the new results are used in the revised Fig.1N.

q3. On line 128, the authors state: "...we further tested by immunofluorescence..." What did you test? I believe that the sentence should say "...we further tested integrin $\alpha X\beta 2$ expression and localization, finding that it was down-regulated when ECM1 was silenced or

ECM1b and ECM1c overexpressed but up-regulated in ECM1a overexpressing cells..."

a3. Yes, thank you very much. We have modified this sentence accordingly in the revised manuscript.

q4. Cisplatin axises in Figure 6 appear to show the wrong scale of concentration (mm)???

a4. We are sorry for this mistake, and have corrected it with the concentration of μM in the revised Figure 7.

Reviewer #3 (Remarks to the Author); expert in ECM and cytoskeleton:

In this manuscript, the authors identify a specific splicing inform of ECM1 as one that promotes tumorigenesis. They go onto show its interaction with a specific integrin and its role in activating and attenuating specific oncogenes. And lastly, they show the presence of ECM-1 correlates with prognosis. The paper is very through in that it explore a numbers of different avenues. However, it is somewhat disjointed for the same reason. At points, it is difficult to understand the transition from experiment to experiment or how the results integrate together. More specific comments below:

Q1. Abstract does not connect well to itself and is difficult to read. The authors need to better connect the ideas and pathways they are pursuing.

A1. Thanks, due to the word limit, we have done our best to carefully modify the abstract. We believe that the ideas and pathways are connected better now.

Q2. Are the results specific to ovarian cancer? This is an important issue to address either experimentally, or to clarify the abstract, title and text by making it more clear this study focuses on ovarian exclusively.

A2. Thanks a lot! In the current study, we only report the identified function of ECM1 in ovarian cancer cells. From the data of parallel experiments, similar functions of ECM1 are being discovered in lung, liver, breast, and other cancers and will be

reported separately in near future. Therefore, we cannot draw the conclusion that the results from this report are specific to ovarian cancer. Some data derived from immortalized fallopian tube epithelial cells, ovarian clear cell carcinoma cells, and breast cancer cells (rFigure.1) fully support this point. The major findings are based on the study of ovarian cancer cells.

Q3. Many cell lines were tested in Figure 1A, and then only A8 cells were used in the other part of Figure 1. Why?

A3. Many thanks. Based on the results in Fig.1A and SFig.1A-C, the highest expression of ECM1 mRNA (ECM1a) and protein was detected in both cell lysates and cultured media of HEY and HEYA8 cell lines, whereas the low expression was detected in other cell lines. Because HEYA8 cells are more aggressive than HEY cells[3], we initially tested HEYA8. To validate the most important findings in this study, we also tested other ovarian cancer cell lines including SKOV3, OVCA429, and OVCA433 (SFig. 2A-I), an immortalized ovarian surface epithelial cell line T29 (SFig. 2J-M), an ovarian clear cell carcinoma cell line ES-2, and an immortalized fallopian tube epithelial cells (FTE) (SFig. 3). The consistent results are obtained from these cell lines.

Q4. The inconsistency between the text labels in the figures and the main text make it difficult to read.

A4. Thanks. We are sorry that the previous manuscript seemed inconsistent between the text labels in the figures and the main text. Due to the word and page limit required by the journal, we had to delete some detailed descriptions. We have carefully modified the figure labels and main text; we believe that the revised manuscript is much clear and consistent.

Q5. Figure 1M- is it is not clear why treatment with the anti-HA antibody would prevent secretion.

A5. Thanks. In A8i-A (HEYA8-ECM1i/ECM1a) cells, the endogenous expression of

ECM1 was silenced, and an exogenous ECM1a with HA tag was introduced into this cell line. While the HA-tagged ECM1a was secreted into medium, treatment of cultured cells with anti-HA antibody could neutralize the secreted ECM1a-HA to prevent the function and signaling of ECM1a-HA, but not to prevent secretion, compared with treatments with PBS or mouse IgG (Fig.1O).

Q6. Did expression of the mutant ECM1 decrease expression of FAK, AKT etc, or did it decrease the phosphorylation (activation) of these signaling molecules?

A6. Good question! Our data revealed that the mutant ECM1a mainly decreases the phosphorylated AKT, FAK, Paxllin, and Rac, compared with the wild type ECM1 (Fig. 3F).

Q7. Line 159 may be referencing the wrong figure?

A7. Thanks. The former line 159 refers to the expression of ABCG1 expression in cells with ECM1a and ECM1b overexpression, and in cells with cross-transfection of ECM1a and ECM1b in ECM1b and ECM1a overexpressing cells (the former Fig. 5D, and the revised Figure 6D). The reference should be correct.

Q8. There is no quantification of western blots

A8. Thanks. We have added the quantification of all western blots by using Image J software.

Q9. How is ECM1 leading to phosphorylation of numerous cancer genes?

A9. Great question! The signaling pathway of ECM1 leading to the phosphorylation of numerous cancer gene proteins including AKT, FAK, Paxillin, Rac, and Myosin is more complicated. First, the binding of ECM1 (ECM1a) to integrin αXβ2 is indispensible for tumorigenesis through the activation of intro-cellular signaling molecules just like traditional ligand-receptor interaction, which may induce the inner membrane molecules like AKT, FAK, Paxillin, and Rac being regulated by ABCG1, or by self-

structural alterations. Although ABCG1 transports ATP, no any available evidence supports the idea that ABCG1 is a kinase. However, our data showed that ABCG1 may interact with these proteins and facilitate their phosphorylation (Fig. 6E). By *in vitro* phosphorylation assay, we found that the eukaryotic ABCG1 can directly phosphorylate AKT2 at serine 473 (SFig. 11A-B), whether the phosphorylation of other molecules including Rac and Myosin is directly regulated by ABCG1 requires more investigations. Moreover, we also found that serine/threonine protein phosphatases and their inhibitors such as the protein phosphatase 2A catalytic subunit beta (PPP2CB) and the protein phosphatase 1 regulatory inhibitor subunit 14C (PP1R14C) may also participate in the phosphorylation regulation of AKT/Rac/Myosin (SFig. 11C-E), because treatment of relative cells with an inhibitor or activator of serine/threonine protein phosphatases 1 and 2A apparently altered the phosphorylation levels of these proteins (SFig. 11F). Please also refer to A6 to Reviewer 1. However, the regulation of the tyrosine-phosphorylated FAK and Paxillin still needs more studies.

Q10. The authors open the manuscript but pointing out the inconsistencies in the literature on ECM-1. It is not clear that they have clarified the literature.

A10. Thanks. Although ECM1 is identified to be oncogenic, but the literature[4] (reference 8 in the former version) is the only report showing that ECM1 might be a tumor suppressor in liver cancer cells. The authors found that silencing of ECM1 by two siRNAs targeting either ECM1a, ECM1b, or ECM1c promotes both cell proliferation and anchorage-independent growth of liver cancer cell lines YY-8103 and QGY7701. Moreover, a very recent study reported that ECM1b localized at ER also inhibits esophagus squamous cell carcinoma (ESCC)[5], which we have cited in our revised manuscript. Therefore, we have clarified the literature for the inconsistence of ECM1 functions, suggesting that the exact function of ECM1 needs a comprehensive investigation.

Reviewer #4 (Remarks to the Author); expert in mRNA alternative splicing:

This is generally a very thorough and well performed study on the role of the ECM1 isoforms 1a, 1b and 1c and their potentially different roles in tumorigenesis. Several previous studies have documented a role for ECM1 in tumorigenesis and ECM1 has been implicated in several regulatory pathways, as for instance TGFB signaling. Knowledge about the potential different roles of the different ECM1 isoforms is however lacking.

The authors initially demonstrate that ECM1 (without distinguishing between the isoforms) is over-expressed in cancers. This is consistent with all previous studies indicating that ECM1 is involved in tumorigenesis.

Using siRNA and overexpression studies the authors convincingly demonstrate that it is in particular the ECM1a isoform which is a tumorigenic driver and that this involves interaction with integrin αXβ2.

One of the major claims in this paper is that the different ECM1 isoforms have different roles in tumorigenesis. Generally, this claim is well supported by the data, but I am a bit worried by the fact that much is based on overexpression (combined with KD) of the three isoforms to point at ECM1a as the tumorigenic isoform.

Q1. Overexpression of the individual isoforms may result in artificially high protein levels and therefore do not mimic the real situation. In fact, there is some effect of overexpressing the ECM1b isoform in KD cells (both on tumor size and AKT activation etc.) - Check fig 2 supplemental.

A1. Many thanks. In SFig.2, all cell lines (433, 429, SKOV3, and T29) were transfected with ECM1a or/and ECM1b without knockdown of the total ECM1 (SFig. 2A, 2D, and 2J). Same experiments were performed in ES-2 (SFig. 3A), FTE cells (SFig. 3B), HeyA8 cells (SFig. 7A), and breast cancer cells (rFigure 1). Please also refer to A2.

Q2. It would be beneficial if the authors had demonstrated the effect of overexpression (in particular the ECM1a and ECM1b isoforms) in cells (and mice?) without shRNA treatment.

A2. Thanks! We overexpressed ECM1a and/or ECM1b in ovarian cancer cell lines OVCA429, OVCA433, and SKOV3, and immortalized ovarian epithelial cell line T29 without shRNA treatment (SFig. 2), overexpressed ECM1b in HeyA8 cells without shRNA treatment (SFig. 7A-C), and overexpressed ECM1a or/and ECM1b in ES-2 and FTE cells (SFig. 3). The results in these figures demonstrated that the cytoskeleton signaling and/or the tumor growth in mice are promoted by ECM1a overexpression, but inhibited by ECM1b introduction without regard to the expression levels of other ECM1 isoforms within the cells.

Q3. I also worry about the expression levels of overexpressed ECM1a, ECM1b and ECM1c. In figure 1D it appears that ECM1a is significantly (2fold? or more?) higher overexpressed than ECM1b.

A3. Many thanks. The previous data in Fig. 1G upper panel (cell lysates-CL, not Fig. 1D**) were generated from the very early batch of cell lines established. We have replaced the previous data with newly tested results in the revised Fig. 1I, showing that the overexpressed protein levels between ECM1a and ECM1b are comparable based on protein band quantification. Moreover, to further address this comment, we introduced ECM1b into ECM1a-overexpressing A8i cells and ECM1a into ECM1b-overexpressing A8i cells. We found that delivery of ECM1b into ECM1a-expressing cells highly inhibited the tumor growth, whereas overexpression of ECM1a in ECM1b-expressing cells moderately increased the tumor growth (SFig. 7I-J). These data suggest that the tumor suppressive function of ECM1b seems more powerful than the tumorigenic role of ECM1a in the tested cell line, although the associated cellular signaling is inversely altered by ECM1b or ECM1a in ECM1a- or ECM1b-expressing cells (Fig. 4I). On the other hand, our data suggest that the tumorigenic discrepancy between ECM1a and ECM1b largely depends on the cellular signaling induced by the ECM1a-integrin αXβ2 interaction, not on the absolute values of the detected ECM1 protein bands.**

Q4. This could have implications for this and subsequent experiments – This tendency of

more ECM1a is also apparent in the co-ip experiments. Therefore the authors need to control better that the level of overexpression is comparable between the three isoforms.

A4. Thanks. We re-examined the expression of ECM1 isoforms in various cell lines and replaced the previous images with new data (Fig. 1I, Fig. 4I, Fig. 5A). The co-IP experiments were performed with overdosed antibodies and total proteins, the results were analyzed qualitatively not quantitatively. We have also quantified the protein expression between ECM1a and ECM1b. We believe that the expression levels between ECM1a and ECM1b are comparable. Please also refer to A3 and A5.

Q5. The authors should demonstrate by shRNA KD of the individual isoforms that ECM1a and not ECM1b and ECM1c is tumorigenic. This should be feasible at least for 1a and 1b isoforms given the fact that exon 7 distinguishes these isoforms.

A5. Good comments! We could not design a shRNA to target both *ECM1b* and *ECM1c*, but we did design the shRNAs specifically targeting exon 7 of *ECM1a* and *ECM1c* because *ECM1b* does not contain exon7. The results showed that silencing of ECM1a and ECM1c enhanced the expression of ECM1b at both mRNA and protein levels, but ECM1b was still not tumorigenic (SFig. 7D-H). On the other hand, introduction of ECM1a into SKOV3, OVCA429, OVCA433, T29, and FTE cells highly induced tumor growth, but introduction of ECM1b into SKOV3, T29, and FTE cells did not induce apparent tumor growth (SFig. 2, SFig. 3C-D). Please also refer to A6.

Q6. Abolishing one isoform at a time by shRNA KD (decreasing the endogenous level) may better reveal their functional difference, since the remaining isoforms will have more realistic protein levels.

A6. Thanks. Because ECM1a or ECM1b cannot be individually silenced in terms of potential ECM1c expression, while ECM1c protein was undetectable by western blot and IHC in ovarian cancer cells, normal ovarian epithelial and fallopian tube epithelial cells and tissues in our study (SFig. 1A-C; SFig. 1F; Fig. 8E). Therefore, we only designed shRNAs targeting the exon 7 of ECM1a and ECM1c mRNAs to test the

function of endogenous ECM1b. The results showed that ECM1b was up-regulated at both mRNA and protein levels in HeyA8 cells, which led to the reduced tumor growth of Hey A8 cells in animals, compared with the tumor growth of control cells (SFig. 7D-H).

Q7. Apart from the potentially questionable comparability between the levels of expression between the three isoforms the experiments very clearly establishes a role for ECM1a in tumorigenesis and that this involves the Akt/Fak/Paxillin/Rho-associated signaling axis in ovarian cancer. The lack of tumorigenicity of ECM1b and ECM1c seems plausible, but is potentially questionable by lower levels of overexpression of in particular ECM1b.

A7. Thanks. We introduced ECM1b into HeyA8, SKOV3, T29, and FTE cells, and silenced ECM1a and ECM1c in HeyA8 cells, but the data firmly suggest that ECM1b is non-tumorigenic and functions as a tumor suppressor. Please refer to A3-A6.

Q8. The authors then use RNA-seq analysis to further detect and delineate the involved pathways. This points them towards integrins αX and β2, ABCG1, and HNRNPLL, which they demonstrate (fig 4) to be dependent on the presence of ECM1a, as knock down leads to a dramatically lower expression that can not be rescued by the ECM1b and ECM1c isoforms (but again: was ECM1b and ECM1c overexpressed at the same level as ECM1a?).

A8. Thanks. We retested the expression of ECM1a and ECM1b after several selections with plasmid-resistant drugs, we found that the expression between ECM1a and ECM1b is comparable based on protein band quantification. Although the introduction of ECM1b into ECM1a-overexpressing cells did not increase the ECM1b expression to the level of ECM1a, the cellular signaling and the tumor growth induced by ECM1a were largely inhibited by the less expressing ECM1b. On the other hand, although the delivery of ECM1a into ECM1b-overexpressing cells increased the ECM1a expression level over ECM1b expression, the tumor growth inhibited by ECM1b was not highly reversed (Fig. 4I; SFig. 7J), indicating that ECM1b may be more powerful than ECM1a. Overall, these data also suggest that the alteration of

cellular signaling and tumorigenesis does not depend on the absolute values of protein expression, and a limited amount of specific protein expression may be enough to reverse the cell fate. p53 may be one of such proteins. Please refer to A3-A7.

Q9. Using HA-tagged proteins and IP they demonstrate that ECM1b and ECM1c interact with non-phosphorylated myosin. This together with other experiments indicate that phosphorylation of myosin is essential for the signaling induced by the ECM1a–integrin αXβ2 interaction.

A9. Thanks, it is true that the phosphorylation of Myosin is essential for the signaling induced by the ECM1a–integrin αXβ2 interaction.

Q10. Next they move on to demonstrate that a positive feedback exist between ECM1a and hnRNPLL, where they both stimulate each others expression. This could have been detailed a bit more, as it is tempting to speculate that hnRNPLL is directly involved in regulating ECM1 splicing, whereas ECM1a stimulation of hnRNPLL expression is likely indirect. It would have been interesting also to know if the expression of other splicing factors than hnRNPLL are changed. The data are, however, consistent with previous studies showing that hnRNPLL expression also promotes colorectal cancer cell proliferation, but it can not be concluded if the observed effect of hnRNPLL is directly on ECM1 splicing or perhaps on mRNA stability.

A10. Great comments! Based on the RNA-seq data (SFig. 5C), we tested other hnRNP splicing factors (functioning as splicing repressors) including hnRNPL, hnRNPM and hnRNPU, and found that these proteins are down-regulated by silencing of ECM1, and up-regulated by ECM1a but not by ECM1b (SFig. 9A-D). However, the direct silencing of these proteins did not alter the mRNA splicing of ECM1 subtypes (SFig. 9E-M). To investigate the counter factors of hnRNPs, we tested SRSFs (Serine/Arginine Splicing Factors functioning as splicing activators) listed in SFig. 10C by qRT-PCR, and found that SRSF1 and SRSF6 were up-regulated by ECM1a and ECM1b, respectively (SFig. 10D-F). We then overexpressed SRSF1 or silenced the expression of SRSF6 in parental

HEYA8 cells (SFig. 10G-H), and found that SRSF1 increased the mRNA splicing of ECM1b, whereas silencing of SRSF6 decreased the ECM1a mRNA splicing (SFig. 10I-L). To test the stability of ECM1 mRNAs, we analyzed the mRNA levels of ECM1 subtypes in hnRNPLL shRNA-expressing cells cultured at 12, 24, 36, and 48 hours, and found that ECM1a mRNA was diminished, whereas ECM1b and ECM1c were slightly raised. However, the mRNA levels of ECM1 subtypes were equally maintained during cell proliferation, indicating that hnRNPLL does not affect the mRNA stability of ECM1 isoforms (SFig. 10A-B).

Q11. Next the authors the authors demonstrate that ABCG1 could mediate the ECM1a-integrin αXβ2 interactive signaling to control tumorigenesis through the regulation of AKT/FAK/Paxillin/Rac/myosin protein phosphorylation and also that ABCG1 overexpression may mediate stemness through upregulation of cd326.

A11. Thanks a lot!

Q12. Finally the authors analyze patient material and demonstrates that the identified proteins may be used as prognostic markers for patients with ovarian cancer.

In conclusion the authors very convincingly demonstrate the role of ECM1a isoform in (ovarian) cancer and they delineate some of the pathways/factors involved. This is new and important knowledge. They also, a little less convincingly show that ECM1b and ECM1c isoforms do not function in same way as ECM1a and that they may even have opposing roles through interaction with different protein partners (for instance phosphorylate Myosin).

A12. Thanks for the great comments. We have provided the convincing data showing that ECM1b and ECM1a inversely regulate cytoskeleton signaling to oppositely control ovarian tumorigenesis,

Minor points:

q1. Generally, I am missing a little more background information about the three isoforms.

Which functional domains are affected etc.

a1. Thanks, we have added more references to describe the more background about the functions of the three isoforms, but we did not find informative literature about the functional domains of ECM1 isoforms.

q2. The claim that hnRNPLL may be targeted for optimal therapy in cancers associated with the ECM1 signaling is a not well documented. hnRNPLL regulates thousands of alternative splicing events, so not only could there be serious side effects from targeting hnRNPLL, but there could potentially be oncogenic effects since most likely hnRNPLL also regulates other protooncogenes and tumor suppressor genes in the opposite direction.

a2. We have modified this description in the revised manuscript.

[Redacted]

References

1. Boylan KL, Andersen JD, Anderson LB, Higgins L, Skubitz AP. Quantitative proteomic analysis by iTRAQ(R) for the identification of candidate biomarkers in ovarian cancer serum. *Proteome science* **8**, 31 (2010).

2. Wen G*, et al.* Immortalization of human fallopian tube epithelial cells *China Oncology* **23**, 241-247 (2013).

3. Yang G*, et al.* The biphasic role of NF-kappaB in progression and chemoresistance of ovarian cancer.

Clinical cancer research : an official journal of the American Association for Cancer Research **17**, 2181-2194 (2011).

4. Gao F*, et al.* Integrated analyses of DNA methylation and hydroxymethylation reveal tumor suppressive roles of ECM1, ATF5, and EOMES in human hepatocellular carcinoma. *Genome biology* **15**, 533 (2014).

5. Yu VZ, Ko JMY, Ning L, Dai W, Law S, Lung ML. Endoplasmic reticulum-localized ECM1b suppresses tumor growth and regulates MYC and MTORC1 through modulating MTORC2 activation in esophageal squamous cell carcinoma. *Cancer letters* **461**, 56-64 (2019).

REVIEWER COMMENTS

Reviewer #2 (Remarks to the Author):

I would like to thank the authors for the thorough attention to my queries. In particular, the clarification the the comments about ECM1a binding and function with dimerization were enlightening with the function blocking antibodies. There are a few minor issues that I believe still remain:

1. In response to my second concern/query, i.e. does ECM1 cover the ECM-binding site on the integrins (?), the authors go through some detail about what ECM1 binds and its downstream effects. However I was wondering if when ECM1 binds to the aXb2 integrins, it renders them non-functional or impairs their ability to bind ligand on the extracellular side, i.e. their typical ligands such as fibrinogen, ICAM-1, and iC3b.

2. Axis for Figure 7A (was 6A) should read micro-M not micro-m. As is, it looks like cisplatin is measured in units of micrometers, not micromoles. The M should be capitalized.

Reviewer #3 (Remarks to the Author):

Overall the manuscript is greatly improved and the story is compelling. I have just two comments:

In the response to reviewers, the authors indicate they identified the presence of ECM1 in other cancers, but I do not see the data in the manuscript. (A1 to Reviewer 1)

There appears to be a type on line 88 of the manuscript with unfinished sentence?

Reviewer #4 (Remarks to the Author):

One of my major concerned overexpression-, siRNA- and IP-experiments. The authors have now supplied new and revised data, which supports their conclusions.

My other main comment concerned a possible cross-regulation/positive feedback between hnRNPLL and ECM1a. The authors have made an effort to shed more light on this, although this in not completely resolved. I am, however, satisfied by the progress made.

I also find that my other questions and concerns have been addressed appropriately.
I therefore recommend publication.

Response to the reviewers' comments

Dear reviewers,

We have addressed all comments properly and we believe that the revised manuscript has met the standard to be accepted by Nature Communications.

Best regards,

The authors

--

Reviewer #1: No comments.

Reviewer #2 (Remarks to the Author):

I would like to thank the authors for the thorough attention to my queries. In particular, the clarification the comments about ECM1a binding and function with dimerization were enlightening with the function blocking antibodies. There are a few minor issues that I believe still remain:

Q1. In response to my second concern/query, i.e. does ECM1 cover the ECM-binding site on the integrins, the authors go through some detail about what ECM1 binds and its downstream effects. However I was wondering if when ECM1 binds to the αXβ2 integrins, it renders them non-functional or impairs their ability to bind ligand on the extracellular side, i.e. their typical ligands such as fibrinogen, ICAM-1, and iC3b.

A1. Thanks! These are good comments. Based on our study, we show that the binding of ECM1 to αXβ2 integrins controls ovarian tumorigenesis and cisplatin resistance through the ABCG1/AKT/FAK/Rho/cytoskeleton-mediated signaling. The interaction between ECM1 and αXβ2 not only promotes the expression of αXβ2, but also facilitates the αXβ2-associated cellular signaling and their biological functions. However, whether the binding of ECM1 to αXβ2 impairs the binding ability of αXβ2 to other ligands may need some investigations.

Following the reviewer's comments, we first reviewed the literature associated with the binding activity of integrin αXβ2 to other ligands. The study by Loike et al reported that CD11c/CD18 (integrin αXβ2) on tumor necrosis factor-stimulated neutrophils may function as a fibrinogen receptor through binding with the sequence Gly-Pro-Arg (GPR) in the alpha chain of fibrinogen (FGA) [1]. However, other studies showed that the gamma chain of fibrinogen (FGG) can be recognized by integrin αXβ2 on leukocytes [2, 3]. Moreover, iC3b was reported to bind with αX subunit of integrin αXβ2 on erythrocytes [4], on neutrophils and monocytes [5] or on alveolar macrophages [6]. Two different studies

revealed that ICAM-1 may be recognized by αX or β2 of αXβ2 [7, 8]. Additional studies identified that the αX I-domain of αXβ2 may also bind to plasminogen (PLG)[9], CD90 (Thy-1)[10], or heparin/PF4 [11].

Thus, to response the reviewer's comments, we first analyzed the expression levels of these ligands in A8 (high ECM1 and αXβ2), A8i (low ECM1 and αXβ2) and A8i-A (high ECM1a and αXβ2) cells by western blot based on the available RNA sequencing data (STable 5). We found that the expression of FGG, ICAM1, PLG, and CD90 appeared lower in A8i cells than in A8 and A8i-A cells, but that of FGA and iC3b in A8i cells was higher than in A8 cells, but lower than in A8i-A cells, whereas heparin/PF4 in A8i cells was higher than in both A8 and A8i-A cells, suggesting that the interaction between ECM1 and αXβ2 may facilitate the expression of FGG, ICAM1, PLG, and CD90, but may not be correspondingly associated with the expression of FGA and iC3b, and that the expression of PF4 seems inversely correlated with the expression of ECM1 and αXβ2 (SFigure 7A).

Next, by co-immunoprecipitation (co-IP) performed with antibodies to αX or β2, we found that αX could bind to all of these ligands, whereas β2 bound to FGG only (SFigure 7B-left panel). The binding abundance of FGA, FGG, PLG, iC3b, and CD90 to αX was correlated with the expression levels of these ligands detected in the corresponding cell lysates, but that of ICAM1 and heparin/PF to αX was not correlated with their expression in cell lines (SFigure 7B-left panel), whereas the binding abundance of FGG to β2 was different from its expression in cell lines (SFigure 7B-right panel). However, the data from the reverse co-IP products using the antibodies to these ligands showed that the binding abundance of αX to FGA, FGG, ICAM1, PLG, and CD90 was higher in A8i cells than in A8 and A8i-A cells, and that the binding abundance of αX to iC3b and PF4 was consistent with that of iC3b and PF4 to αX, the binding abundance of β2 to FGG was inversely correlated with that of FGG to β2 (SFigure 7B-right panel and SFigure 7C). These data suggest that the binding of ECM1a to αXβ2 may differentially regulate the function of FGA, FGG, ICAM1, PLG, iC3b, CD90, and PF4, and their binding abilities to integrin αXβ2, although more detailed investigations are needed for each of these ligands because they may also functionally bind to the molecules other than αXβ2.

Q2. Axis for Figure 7A (was 6A) should read micro-M not micro-m. As is, it looks like cisplatin is measured in units of micrometers, not micromoles. The M should be capitalized.

A2. Thanks a lot! The concentration unit of cisplatin in this study is μM, which has been corrected in all panels of Figure 7.

Reviewer #3 (Remarks to the Author):

Overall the manuscript is greatly improved and the story is compelling. I have just two comments:

Q1. The response to reviewers, the authors indicate they identified the presence of ECM1 in other cancers, but I do not see the data in the manuscript. (A1 to Reviewer 1)

A1. Thanks for the critical comments. Here, we have provided the presence of ECM1 in both cell lines and clinical tissues of cervical cancer, breast cancer, hepatocellular cancer, and lung cancer. However, because these data will be separately published later by different authors, we did not integrate them into the revised manuscript.

As shown in rFigure 1A, in five cervical cancer cell lines, both ECM1a and ECM1b were detected by semi-quantitative RT-PCR and western blot in MS751 and ME-180 cell lines, whereas only was ECM1a detected in Hela, Siha, and Ca-Ki cell lines. ECM1a but not ECM1b and ECM1c were detected in the tested cell lines of breast cancer, liver cancer and lung cancer (rFigure 1B).

Analysis of clinical tissues by IHC in rTable 1 showed that weak and strong staining accounted for 19.4% (6/31) and 38.7% (12/31), respectively, in 31 cervical squamous cell carcinoma tissues. In 60 invasive breast ductal carcinoma tissues, 30% (18/60) or 21.7% (13/60) were weakly or strongly detected, whereas 12.1% (7/58) or 37.9% (22/58) cases were weakly or strongly detected in 58 hepatocellular carcinoma tissues. In 57 lung cancer tissues including subtypes of NSCLC, and SCLC, weak and strong staining were 50.9% (29/57) and 29.8% (17/57), respectively. All adjacent (normal) tissues of the cancer cases were negatively stained. The representative images are shown in rFigure 2.

Q2. There appears to be a type on line 88 of the manuscript with unfinished sentence?

A2. Thanks, the last word is "IHC", which has been added in this sentence.

Reviewer #4 (Remarks to the Author):

One of my major concerned overexpression-, siRNA- and IP-experiments. The authors have now supplied new and revised data, which supports their conclusions.

My other main comment concerned a possible cross-regulation/positive feedback between hnRNPLL and ECM1a. The authors have made an effort to shed more light on this, although this in not completely resolved. I am, however, satisfied by the progress made.

I also find that my other questions and concerns have been addressed appropriately.
I therefore recommend publication.

Many thanks for these favorable comments.

References

1. Loike JD*, et al.* CD11c/CD18 on neutrophils recognizes a domain at the N terminus of the A alpha chain of fibrinogen. *Proceedings of the National Academy of Sciences of the United States of America* **88**, 1044-1048 (1991).

2. Lishko VK, Yakubenko VP, Hertzberg KM, Grieninger G, Ugarova TP. The alternatively spliced alpha(E)C domain of human fibrinogen-420 is a novel ligand for leukocyte

integrins alpha(M)beta(2) and alpha(X)beta(2). *Blood* **98**, 2448-2455 (2001).

3. Ugarova TP, Yakubenko VP. Recognition of fibrinogen by leukocyte integrins. *Annals of the New York Academy of Sciences* **936**, 368-385 (2001).

4. Bilsland CA, Diamond MS, Springer TA. The leukocyte integrin p150,95 (CD11c/CD18) as a receptor for iC3b. Activation by a heterologous beta subunit and localization of a ligand recognition site to the I domain. *Journal of immunology* **152**, 4582-4589 (1994).

5. Myones BL, Dalzell JG, Hogg N, Ross GD. Neutrophil and monocyte cell surface p150,95 has iC3b-receptor (CR4) activity resembling CR3. *The Journal of clinical investigation* **82**, 640-651 (1988).

6. Ross GD, Reed W, Dalzell JG, Becker SE, Hogg N. Macrophage cytoskeleton association with CR3 and CR4 regulates receptor mobility and phagocytosis of iC3b-opsonized erythrocytes. *Journal of leukocyte biology* **51**, 109-117 (1992).

7. Frick C*, et al.* Interaction of ICAM-1 with beta 2-integrin CD11c/CD18: characterization of a peptide ligand that mimics a putative binding site on domain D4 of ICAM-1. *European journal of immunology* **35**, 3610-3621 (2005).

8. Choi J, Choi J, Nham SU. Characterization of the residues of alphaX I-domain and ICAM-1 mediating their interactions. *Molecules and cells* **30**, 227-234 (2010).

9. Gang J, Choi J, Lee JH, Nham SU. Identification of critical residues for plasminogen binding by the alphaX I-domain of the beta2 integrin, alphaXbeta2. *Molecules and cells* **24**, 240-246 (2007).

10. Choi J, Leyton L, Nham SU. Characterization of alphaX I-domain binding to Thy-1. *Biochemical and biophysical research communications* **331**, 557-561 (2005).

11. Vorup-Jensen T*, et al.* Binding between the integrin alphaXbeta2 (CD11c/CD18) and heparin. *The Journal of biological chemistry* **282**, 30869-30877 (2007).

<u>rTables and rFigures are attached below.</u>

[Redacted]

[Redacted]

[Redacted]

[Redacted]

REVIEWERS' COMMENTS

Reviewer #2 (Remarks to the Author):

I would like to thank the authors for their very thorough analysis of ECM1a binding to αXβ2. The differential regulation of function and binding is very interesting and I thank them for this clarification. I think that this will be a nice addition to the literature.

Reviewer #3 (Remarks to the Author):

The authors have adequately addressed my concerns. Congratulations on a terrific paper.

Response to REVIEWERS' COMMENTS

Dear reviewers,

We are highly grateful for your favorable comments.

Reviewer #2 (Remarks to the Author):

Comment:

I would like to thank the authors for their very thorough analysis of ECM1a binding to αXβ2. The differential regulation of function and binding is very interesting and I thank them for this clarification. I think that this will be a nice addition to the literature.

Response: Many thanks for your favorable comments.

Reviewer #3 (Remarks to the Author):

Comment: The authors have adequately addressed my concerns. Congratulations on a terrific paper.

Response: Many thanks for your great comment.

With our best regards,

Gong Yang and the team

2021-05-06
